# Antibiotics change the population growth rate heterogeneity and morphology of bacteria

**DOI:** 10.1371/journal.ppat.1012924

**Published:** 2025-02-05

**Authors:** Morten Kals, Emma Kals, Jurij Kotar, Allen Donald, Leonardo Mancini, Pietro Cicuta

**Affiliations:** 1 Cavendish Laboratory, University of Cambridge, Cambridge, United Kingdom; 2 Synoptics Ltd., Cambridge, United Kingdom; UCSD: University of California San Diego, UNITED STATES OF AMERICA

## Abstract

A better understanding of the system-level effects of antibiotics on bacterial cells is essential to address the growing challenge of antibiotic resistance. Utilising Multipad Agarose Plate (MAP) platforms, we monitor the growth rate and cell morphology of three clinically relevant species (*E.coli*, *S.aureus* and *P.aeruginosa*) following exposure to 14 antibiotics across 11 concentrations (31 microbe-antibiotic combinations in total). Our results reveal a consistent increase in population growth rate heterogeneity (PGRH) as drug concentrations approach the minimum inhibitory concentration (MIC). Strikingly, the magnitude of this heterogeneity correlates with the functional distance between the ribosome and the specific cellular processes targeted by the antibiotics. Among the seven antibiotic classes studied, protein synthesis inhibitors and disruptors cause the lowest PGRH, while heterogeneity progressively increases with RNA synthesis inhibitors, DNA replication inhibitors, cell membrane disruptors and cell wall synthesis inhibitors. Because the ribosome is central to growth rate control, we hypothesize that heterogeneity might arise at the system level as a result of the propagation of damage to protein synthesis. Low heterogeneity is desirable from a clinical perspective, as high heterogeneity is often associated with persistence and treatment survival. Additionally, we observed a strong correlation between morphological alterations and growth inhibition across all antibiotics and species tested. This led to the development of a novel morphological parameter, MOR_50_, which enables rapid estimation of MIC for antibiotic susceptibility testing (AST) with a single snapshot after just 2.5 hours of incubation. In addition to introducing a novel, resource-efficient and rapid AST method, our findings shed new light on the system-level effects of antibiotic perturbations on bacteria, which might inform treatment design.

## Author summary

Antibiotic drugs affect bacterial cells well beyond their specific molecular targets, and understanding their system-level effects could be key to combating antibiotic resistance. Using a recently developed high throughput imaging platform, Multipad Agarose Plate (MAP), we tracked the growth rates and cell morphology of three species -*E. coli, S. aureus, and P. aeruginosa*- under exposure to 14 antibiotics at 11 different concentrations, resulting in 31 microbe-antibiotic combinations. The findings reveal a consistent increase in population growth rate heterogeneity (PGRH) as antibiotic concentrations near the minimum inhibitory concentration (MIC), and a correlation between the level of heterogeneity and the functional distance from the ribosome to the antibiotics’ target processes. Among the seven antibiotic classes tested, protein synthesis inhibitors lead to the lowest PGRH, while heterogeneity escalates with inhibitors of RNA synthesis, DNA replication, cell membrane disruptors, and cell wall synthesis inhibitors. This suggests that heterogeneity may stem from damage to protein synthesis due to the ribosome’s role in growth control. Low heterogeneity is favourable from a healthcare perspective, as it relates to reduced bacterial persistence. A new morphological parameter, MOR50, is developed allowing very quick MIC estimation in antibiotic susceptibility testing (AST), potentially enhancing the speed and efficiency of treatment.

## 1 Introduction

Antibiotics interact with specific molecular targets within bacterial cells to inhibit essential processes. These disruptions initiate cascades of events that eventually lead to two phenotypes: growth arrest and death [1]. Although the specific molecular targets of most antibiotics are well characterised [2], the indirect processes that ultimately impair cellular growth are complex and not fully understood. A comprehensive understanding of the system effects of antibiotics at the single-cell level would shed light on the inner workings of bacteria and could improve the rational design of treatment.

Cell size is one of the properties that bacteria regulate at the system level. It is the complex, intensively studied, and still not fully understood result of the coordination of the rates of synthesis of the cell’s envelope, its DNA and its many other cytoplasmic contents. These rates, in turn, depend on ribosome activity and metabolism [3]. For *E.coli* in balanced growth, cell size has a strong positive correlation with growth rate and ribosomal content [3, 4]. Perturbations, such as those caused by antibiotics, can decouple such relations. For example, decreased DNA replication rates can lead to increased cell sizes [3], while partial inhibition of the ribosomal pool enlarges cells in nutrient-poor medium and shrinks them in rich medium [4]. However, the field does not have a holistic picture of how antibiotics impact cell morphology, especially where such changes would be linked to broader effects at the cell system level, rather than as a direct consequence of the antibiotic target being inactivated.

In bacteria, growth rate is also controlled at the system level. When measuring antibiotic efficacy, a significantly altered growth rate is typically the easiest phenotype to observe: the minimum concentration of antibiotic that leads to growth inhibition, known as the minimum inhibitory concentration (MIC), has guided clinical research and diagnostics for decades [5]. Several other aspects of growth can be studied to gain insight into the action of antibiotics. Growth rate changes due to antibiotics at sub-inhibitory concentrations, for example, have helped shed light on important questions concerning how cells grow and regulate their macromolecular composition [6], and how they respond to perturbations [7]. The relationship goes both ways, and the cell’s growth rate can in turn alter antibiotic efficacy [8, 9]. Important bactericidal antibiotic classes, such as aminoglycosides and beta-lactams, are less effective against slow and non-growing cells [10, 11]. Complex systems such as bacteria are intrinsically heterogenous [12] and bacterial populations simultaneously harbour fast, slow, and non-growing cells [13]. Heterogeneity in a genetically identical cell population is an important evolutionary trait, and it is the basis of the observed persistence to many antibiotic treatments [14]. Persister cells with reduced growth rates have been extensively studied in vitro [15] and are thought to drive infection relapses *in vivo* [16]. Interestingly, some types of persistence are thought to be triggered by exposure to antibiotics, an unwanted effect from the point of view of therapy [14].

Some of these system-level effects have now begun to be revealed. For example, our lab has demonstrated that an antibiotic such as rifampicin, which targets RNA polymerase [17], can reduce macromolecular crowding [18, 19]. This reduction is a severe, system-level perturbation that impacts the rates of nearly all cellular biochemical processes. We observed similar effects with ciprofloxacin, a fluoroquinolone that interacts with DNA gyrases [19]. Beta-lactam antibiotics, that compromise cell wall integrity, are also known to cause toxic system effects by promoting a futile cycle of cell wall precursors that depletes cellular resources [20]. Aminoglycosides, which interact with the ribosome to cause translation errors [21], are also thought to disrupt cell integrity [22], impacting the cell’s energy production mechanisms [23]. Universal system-level bactericidal strategies based on the production of reactive oxygen species (ROS) have been proposed [24], spurring debate [25–28], and finding a certain degree of experimental support [29–31]. Whether by means that are ultimately universal or not, it is clear that antibiotics interact with the complex system that is the cell, introducing perturbations that can be observed in molecular pathways far beyond where they started.

**Fig 1:**
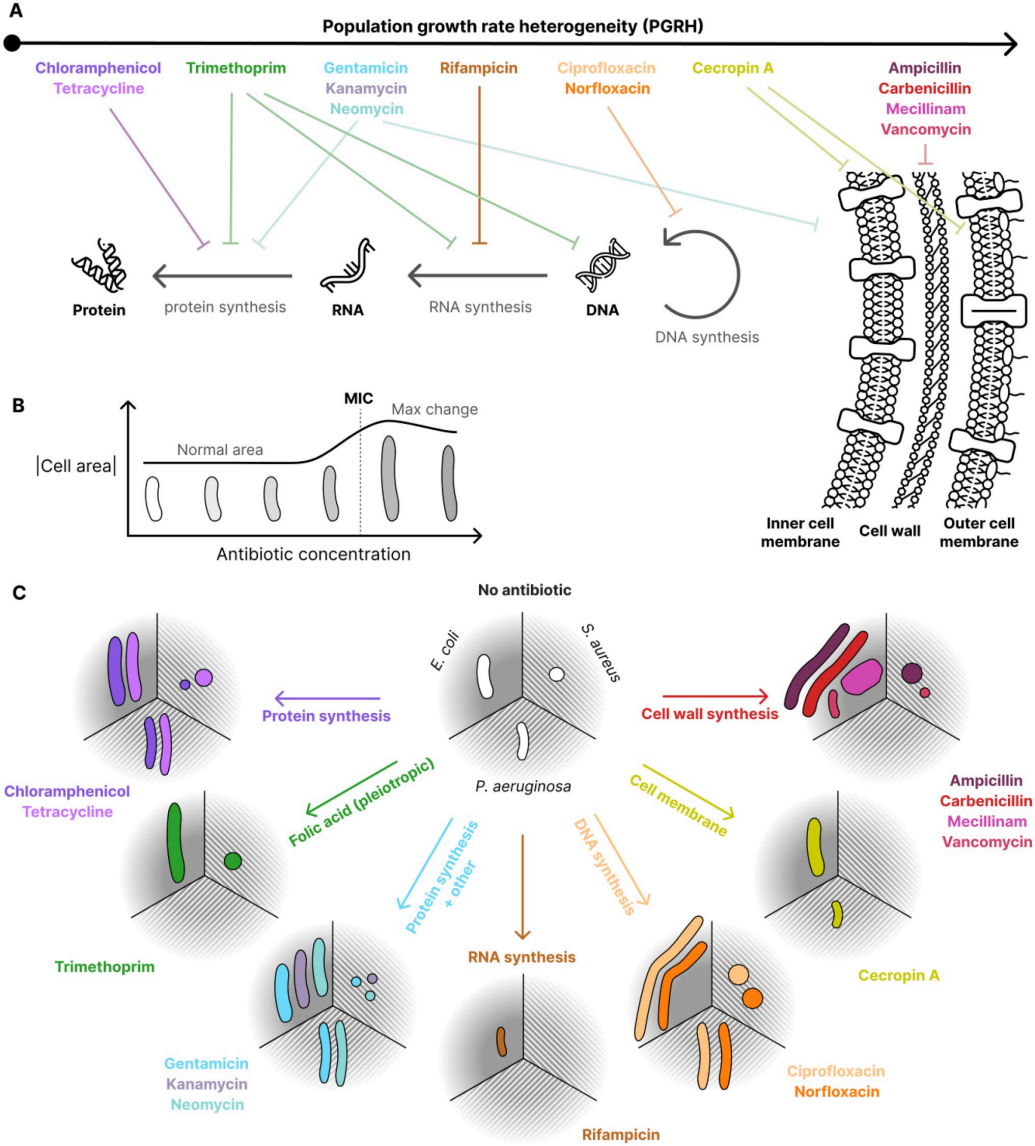
This study examines a range of drugs targeting different cellular functions in three bacterial species, using various system-level readouts to analyze their effects. **A** Diagram of the antibiotics used in this study, ranked by the population growth rate heterogeneity (PGRH) they induce, alongside their cellular targets. Protein synthesis inhibitors, which directly limit protein production, induce the lowest PGRH, while cell wall synthesis inhibitors induce the highest. This suggests a potential link between PGRH and the functional distance from protein production. **B** Schematic plot illustrating that morphological changes occur only at antibiotic concentrations that impact growth. While the magnitude of these changes varies greatly (as shown in **C**), normalization reveals a consistent general pattern across antibiotics, irrespective of their mechanism of action. This novel link between morphology change and growth inhibition led us to develop a new heuristic metric, MOR_50_, which can be used for rapid and high throughput MIC estimation. **C** Schematic showing the morphological impacts of different antibiotics for the three tested bacterial species. The white cell masks in the centre represent the typical morphologies of each bacteria species without an antibiotic present, while the coloured cells represent changes induced by the antibiotics. Some antibiotics increase cell size, while others decrease it. Data for this plot is provided in Section 2.3.

We recently presented a new experimental platform that enables the high-throughput optical imaging of live microbes across different environmental conditions, the Multipad Agarose Plate (MAP) [32]. In this work, we leverage and extend the MAP to perform a systematic investigation of the growth dynamics and morphological responses of three clinically relevant bacterial species (*E.coli*, *P.aeruginosa* and *S.aureus*). We probe these bacteria out of balanced growth, subjecting them to a panel of 14 antibiotics at a range of concentrations and capturing sub-MIC, MIC and post-MIC behaviour. The platform and the accompanying analysis pipeline allow the extraction of single cell and colony parameters directly from images taken with brightfield illumination, so that samples can be completely label-free. Our results reveal that, approaching the MIC, antibiotics systematically increase the heterogeneity of growth rate across microcolonies. Although the role of this heterogeneity in bacterial survival is not yet clear, it is intriguing to study it in light of its intrinsical link to persister formation. Notably, we observe a striking correlation between population growth rate heterogeneity (PGRH), and functional distance from ribosome to the cellular processes targeted by the antibiotic. This pattern holds true regardless of whether the antibiotic is bacteriostatic or bactericidal. For instance, the bacteriostatic rifampicin causes greater heterogeneity than the primarily translation-targeting bactericidal aminoglycosides (Fig 1A). The latter, in turn, leads to heterogeneity levels that are closer to those caused by the ribosome-targeting chloramphenicol.

PGRH is not the only factor that shows a striking correlation with the MIC. Our investigation of single-cell morphologies across the 14 antibiotics and three microbial species shows that all antibiotics cause morphological alterations (Fig 1C), and unveils a strong correlation between the degree of morphological alteration and the MIC (Fig 1B). Using dyes, several works have previously linked antibiotic efficacy [33] and even mechanism of action [34] with morphology using single-cell imaging. Using our insight on label-free morphology alone, we introduce a simple heuristic metric that we call *morphological change 50* (MOR_50_). MOR_50_ is the lowest antibiotic concentration that, after incubation with exponentially growing cells, induces a single-cell morphological change which exceeds 50% of the largest morphological change induced at any concentration of that antibiotic. We show that MOR_50_ can be used to reliably determine the MIC from a single snapshot captured after 2.5 hours of antibiotic exposure.

Taken together, our results identify an extensive and un-appreciated tendency towards heterogeneous growth among pathogens when exposed to sub-inhibitory concentrations of antibiotics that do not directly inhibit protein synthesis. We expect that this will motivate further research aimed at understanding whether such phenomena may lead to the formation of persisters, and whether their formation relies on mechanisms that require protein synthesis [35]. We further argue that our new metric, MOR_50_, could pave the way for a new generation of rapid phenotypic AST methods, potentially transforming the landscape of microbial diagnostics and antibiotic therapy.

## 2 Results

### 2.1 The MAP platform accurately measures the MIC of *E.coli*, *P.aeruginosa* and *S.aureus* based on growth rate

**Fig 2:**
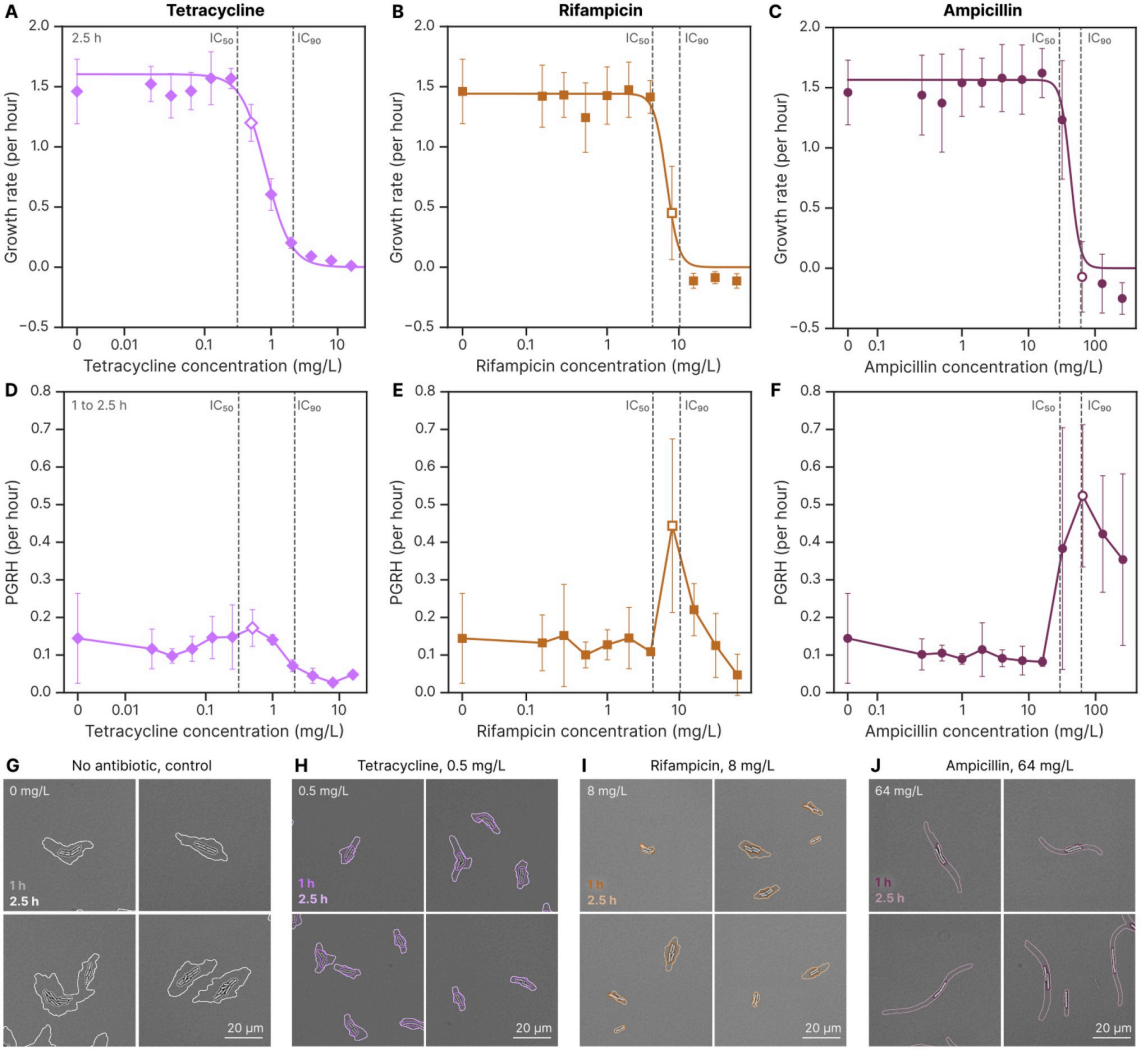
Different antibiotics affect bacterial growth rate and population growth rate heterogeneity (PGRH) differently, as illustrated by *E.coli* exposed to three antibiotics from our test set. The concentration selected to represent PGRH for subsequent analysis has a white-centred marker. **A** Effect of tetracycline (a protein synthesis inhibitor) on *E.coli* colony growth rates after 2.5 hours of growth on the MAP. A Hill equation was used to fit the data and determine the concentrations corresponding to 10% (IC_10_) and 90% (IC_90_) growth inhibition. Each point represents the mean and standard deviation from four biological repeats. **B** Effect of rifampicin (an RNA synthesis inhibitor) on *E.coli* growth rates. Data represent five biological repeats. **C** Effect of ampicillin (a cell wall synthesis inhibitor) on *E.coli* growth rates. Data represent six biological repeats. **D** Effect of tetracycline on the PGRH of *E.coli* during 1 to 2.5 hours of growth on the MAP. PGRH is defined as the standard deviation of colony growth rates at a given time point, averaged across multiple time points and biological repeats. **E** Effect of rifampicin on *E.coli* PGRH. **F** Effect of ampicillin on *E.coli* PGRH. **G** Control showing how growth rate typically varies between microcolonies when there is no antibiotic present. Colony images after 1 hour of growth on the MAP are shown alongside their segmentation masks. The mask from an additional 1.5 hours of growth is overlaid in a lighter hue, highlighting differences in colony growth. **H** Example showing the heterogeneous effects of the selected tetracycline concentration on colony growth. **I** Example of heterogeneous effects of rifampicin. **J** Example of heterogeneous effects of ampicillin.

Leveraging the recently developed MAP platform [32], we first set out to assay the MIC of a panel of 14 antibiotics against *E.coli*, *P.aeruginosa* and *S.aureus*, three human commensals that can turn pathogenic. This was done based on the tracking of growth rate over the first three hours after exposure to antibiotics (S1 Fig). Each dataset consists of data from biological quadruplicates, each consisting of approximately 30 technical replicates. As previously [32], we used Hill curves to model growth rate as a function of antibiotic concentration with examples shown for *E.coli* exposed to tetracycline, rifampicin and ampicillin in Fig 2A–2C respectively (see S2 Fig for all antibiotics and species). The fit is highly time-dependent for some antibiotics because death or growth halt occurs only after a significant amount of damage has accumulated in the cell or after the action of a significant amount of molecular targets of the antibiotic has been blocked. It is, therefore, crucial to pick the time window used for analysis carefully. We chose to use the 2.5-hour time-point for the MIC determination, as by then, all of the different antibiotics had performed their action (bacteriostatic or bactericidal). This delay is required for cell wall synthesis inhibitors especially, as they have little impact on colony growth rate within the first hour (S1 Fig). See S3 Fig for fits performed on each repeat for all combinations of species and antibiotics. The Hill exponent *n* measures the steepness of the drop between growing and non-growing conditions across the various antibiotic concentrations, with larger values indicating a steeper drop. As MIC, we use the 90% inhibitory concentration (IC_90_) where growth rate is reduced by 90%. The correspondence with the broth microdilution assay (generally accepted as a gold standard method for phenotypic AST) and EUCAST values confirmed the validity of the platform for the assay of antimicrobial susceptibility beyond *E.coli*, see S4 and S5 Figs respectively. The fold difference between MIC as measured by growth rate on the MAP and the MIC obtained from broth microdilution is 0.9 ± 0.7 (mean ± SD, base 2), meaning the two methods, on average, produce a MIC concentration within a factor of 2 of each other.

### 2.2 Antibiotics affect population growth rate heterogeneity (PGRH) depending on the cell function they target

Among the antibiotics considered, we observed an interesting pattern in a metric we call population growth rate heterogeneity (PGRH). PGRH is defined as the standard deviation of colony growth rates at a given time point, averaged across multiple time points. The metric reflects the variability in growth rates between colonies under otherwise identical conditions. PGRH is computed for each pad and then averaged across repeats in the 1 to 2.5-hour time period. Fig 2D–2F shows how tetracycline produces only a minor increase in PGRH, occurring at a concentration where growth is partially inhibited. In contrast, rifampicin causes a substantial increase in PGRH, and ampicillin results in an even greater increase (see S6 Fig for all antibiotics and species). We pick the local maxima of PGRH for concentrations with inhibited growth as the representative PGRH of that antibiotic/species combination. To visualize this heterogeneity, Fig 2G–2J shows example colonies and their growth after 1 and 2.5 hours on the MAP. Colonies treated with tetracycline exhibit similar area changes, whereas those treated with rifampicin and ampicillin display pronounced variability. Some colonies show little or no growth, while others grow significantly. Among the antibiotics considered, we observed an interesting pattern in a metric we call population growth rate heterogeneity (PGRH). PGRH is defined as the standard deviation of colony growth rates at a given time point, averaged across multiple time points. The metric reflects the variability in growth rates between colonies under otherwise identical conditions. PGRH is computed for each pad and then averaged across repeats in the 1 to 2.5-hour time period. Fig 2D–2F shows how tetracycline produces only a minor increase in PGRH, occurring at a concentration where growth is partially inhibited. In contrast, rifampicin causes a substantial increase in PGRH, and ampicillin results in an even greater increase (see S6 Fig for all antibiotics and species). We pick the local maxima of PGRH for concentrations with inhibited growth as the representative PGRH of that antibiotic/species combination. To visualize this heterogeneity, Fig 2G–2J shows example colonies and their growth after 1 and 2.5 hours on the MAP. Colonies treated with tetracycline exhibit similar area changes, whereas those treated with rifampicin and ampicillin display pronounced variability. Some colonies show little or no growth, while others grow significantly.

**Fig 3:**
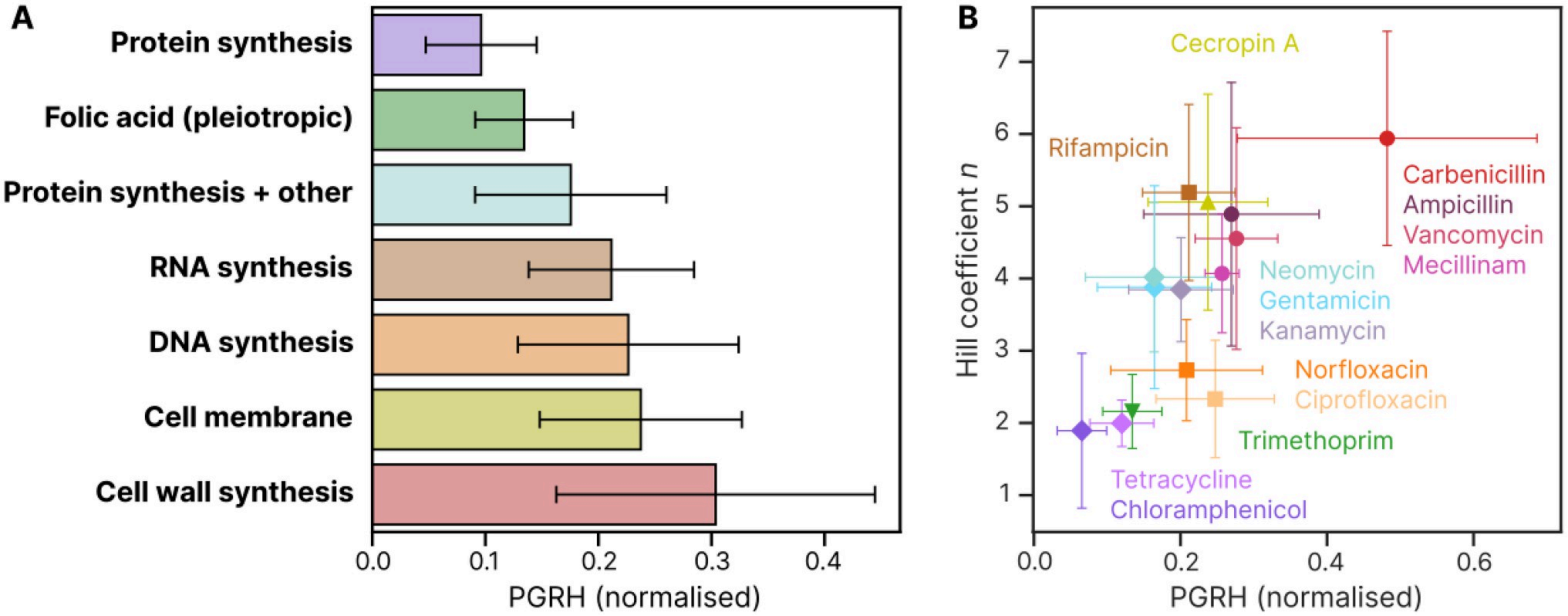
Protein synthesis inhibitor antibiotics cause the smallest population growth rate heterogeneity (PGRH), while cell wall synthesis inhibitors cause the largest PGRH. **A** PGRH, normalized to the growth rate in the absence of antibiotics, averaged across *E.coli*, *S.aureus* and *P.aeruginosa* and grouped by antibiotic target. The antibiotic classes are ordered by PGRH from least to greatest. Bars represent the mean and standard deviation across antibiotic/species combinations. A detailed version of this plot, showing individual antibiotics and species, is provided in S8 Fig. **B** Correlation between PGRH and the Hill coefficient for each antibiotic. Data points represent the mean and standard deviation across species for each antibiotic.

There is a consistent change in PGRH around concentrations of partial growth inhibition for all antibiotic/species combinations (S6 Fig). Fig 3A shows how PGRH is changed the least for the protein synthesis inhibitors (sometimes causing a decrease, sometimes a small increase), whereas the other classes cause increases of varying magnitude starting with folic acid synthesis inhibitors, RNA and DNA synthesis inhibitors, before ending with cell membrane and cell wall synthesis inhibitors producing the largest PGRH (see S7 Fig for the full breakdown). This trend largely holds true for the individual species as well, as shown in S8 Fig, with some notable exceptions being that antibiotics that target protein synthesis in addition to other targets have much larger heterogeneity in *S.aureus* and *P.aeruginosa* than in *E.coli*. Also, the PGRH induced by DNA synthesis inhibitors in *P.aeruginosa* is significantly larger than in the other species. These changes might reflect species-specific characteristics of antibiotic actions.

We also observed a correlation between PGRH and the Hill fitting parameter *n*, which describes the steepness of the growth rate vs antibiotic concentration curve as it transitions from growth to non-growth (Fig 3B). Antibiotic classes exhibited clustering: protein synthesis inhibitors had both low PGRH and *n*, while cell wall synthesis inhibitors showed high PGRH and *n*. Other classes fell between these extremes. Notably, protein synthesis inhibitors with additional targets show higher *n* values while maintaining low PGRH, whereas DNA synthesis inhibitors showed a larger increase in PGRH while maintaining low *n*. This pattern of clustering largely holds true for individual species as well (S9 Fig).

Heterogeneity appears to depend on the cell function targeted by the specific antibiotic class. This would seem to exclude major contributions by mechanisms that are known to cause heterogeneity in antibiotic response and that are class-independent or cross-class. One example of such a mechanism is efflux pumping. To test whether and in what measure heterogeneity depends on these phenomena, we conducted experiments using an *E.coli* mutant lacking the TolC transporter, a key component of several efflux pumps, including the multidrug efflux pump AcrAB-TolC. Antibiotics like ciprofloxacin (which interferes with DNA replication) and tetracycline (a protein synthesis inhibitor that produces one of the lowest PGRH) are among its substrates, making this mutant a useful model for assessing the impact of efflux activity on PGRH. The mutant exhibited a significantly lower MIC compared to the corresponding *E.coli* wild-type strain, consistent with reduced efflux capacity (S10 Fig). However, there was no significant difference in PGRH between the wild type and the mutant when exposed to either ciprofloxacin or tetracycline. This suggests that while the TolC transporter contributes to overall antibiotic resistance, its absence does not appear to influence the variability in growth rates between colonies under the tested conditions.

### 2.3 All antibiotics induce changes in cell morphology

**Fig 4:**
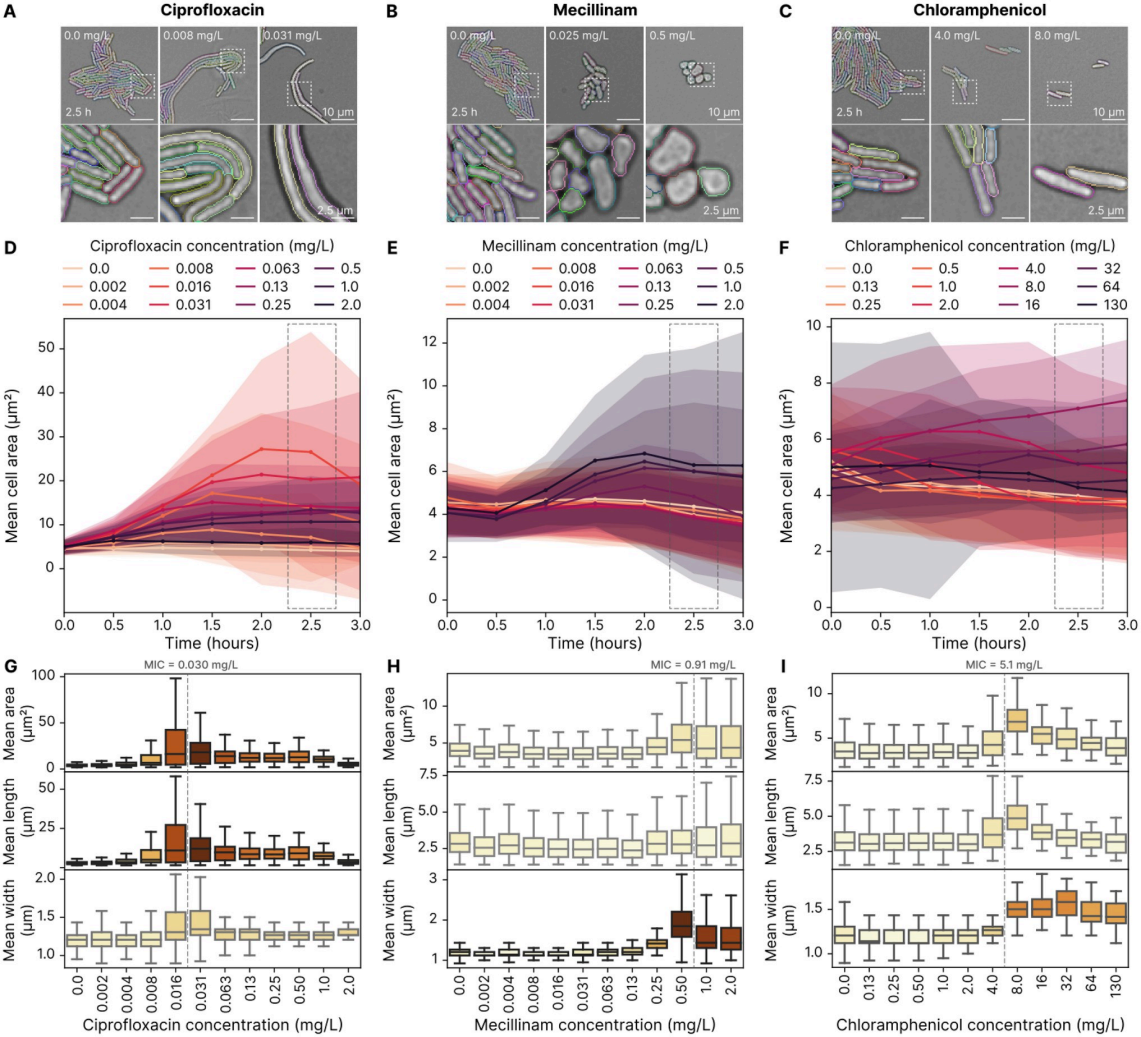
The morphology of *E.coli* changes significantly when exposed to antibiotics. **A** Representative colonies from pads with varying ciprofloxacin (DNA synthesis inhibitor) concentrations demonstrate the antibiotic’s impact on cell morphology after 2.5 hours of incubation. Single-cell segmentation masks are overlaid, with each cell shown in a different colour. Ciprofloxacin induces clear filamentous growth. The bottom row provides a 4× zoom of the corresponding frames from the top row. **B** Mecillinam (cell wall synthesis inhibitor) causes the cells to grow into a more spherical shape. **C** Chloramphenicol (protein synthesis inhibitor) causes the cells to grow larger. **D** Filamentous growth becomes evident over time, with the most pronounced differences observed around 2 hours of growth for ciprofloxacin. Each line represents the mean cell area at a given antibiotic concentration, with shaded areas indicating the standard deviation from four repeats. Darker colours represent higher antibiotic concentrations, and data points are binned at 30-minute intervals. **E** Changes induced by mecillinam occur with a similar time delay. Data is based on four repeats. **F** Changes induced by chloramphenicol occur more quickly, becoming evident within the first hour of growth. Data is based on four repeats. **G** At 2.5 hours of growth, morphology data show that antibiotic concentrations near the MIC induce the most substantial changes in cell morphology, with significant area increases primarily driven by cell elongation. The MIC, averaged across four repeats, is indicated by the vertical line. The boxplot displays the median (line) and interquartile range (box), with whiskers extending to 1.5 times the interquartile range. Data is based on four repeats. **H** For mecillinam, morphology data at 2.5 hours reveal that the most pronounced change is an increase in cell width, with minimal impact on length. These changes are most prominent at concentrations close to the MIC. Data is based on four repeats. **I** Chloramphenicol induces moderate increases in both cell length and width, with the largest magnitude close to the MIC. Data is based on four repeats.

Next, we turned our attention to single-cell morphology. Using the same datasets as for the growth rate analysis, we performed single-cell segmentation on the micrographs to compute cell areas, lengths and widths. To do so, we expanded our analysis pipeline to include a classical cell segmentation step, informed by the colony segmentation. Fig 4A shows the *E.coli* cell segmentation masks atop brightfield microscopy data, highlighting that our analysis pipeline can appropriately segment *E.coli* cells that grow filamentous in response to ciprofloxacin. For comprehensive benchmarking of this algorithm on the three species, see S2 Table. In Fig 4D, we show how the morphology change is time-dependent. All cells start out with the same morphology when seeded on the pads, and only after some time do changes caused by the antibiotic begin to appear. It is, therefore, important to be consistent with the experimental protocol and time-point used for analysis. We show that morphological changes are concentration-dependent by taking data from the 2.5-hour period again and plotting morphology parameters against antibiotic concentration. Cells treated with ciprofloxacin only elongate in a subset of the concentrations tested, presumably because low doses are insufficient to cause changes and high doses kill the cells quickly, not giving them time to grow at all (Fig 4G).

The cell wall synthesis inhibitor mecillinam also causes morphological alterations in *E.coli*. While these manifested mainly in length for ciprofloxacin, mecillinam causes the cells to grow into spheroids (Fig 4B). These are also well-segmented by our pipeline. Similar to ciprofloxacin, the change in width is time and concentration-dependent, with the largest shapes occurring after 2 hours of growth (Fig 4E and 4H).

The protein synthesis inhibitor chloramphenicol also produces an increase in cell size (Fig 4C). The increase becomes apparent only after some delay as a consequence of growth and division (Fig 4F). Like in the other cases, the change in size is concentration-dependent and caused by both increased width and length (Fig 4I).

A final example we will mention is vancomycin, a cell wall synthesis inhibitor that is the only antibiotic tested that causes *E.coli* cells to shrink in size. Again, the shrinkage becomes apparent only after some delay as a consequence of growth and division (S11 Fig). Like in the other cases, the change in size is concentration-dependent, and it is due to a reduction in length that dominates the small increase in width (S12 Fig).

Taken together, these results suggested that, for cells growing exponentially at the time of seeding, the largest morphological changes generally occur after about 2.5 hours of growth on the MAP. This corresponds to approximately 6 doublings for *E.coli* (whose doubling time is 24 min on the MAP), and four doublings for *S.aureus* (37 min) and *P.aeruginosa* (36 min). See S11 Fig for plots highlighting morphology over time for all the tested antibiotics with *E.coli*, *P.aeruginosa* and *S.aureus*, and S12 Fig for all plots of morphology vs antibiotic concentration.

**Fig 5:**
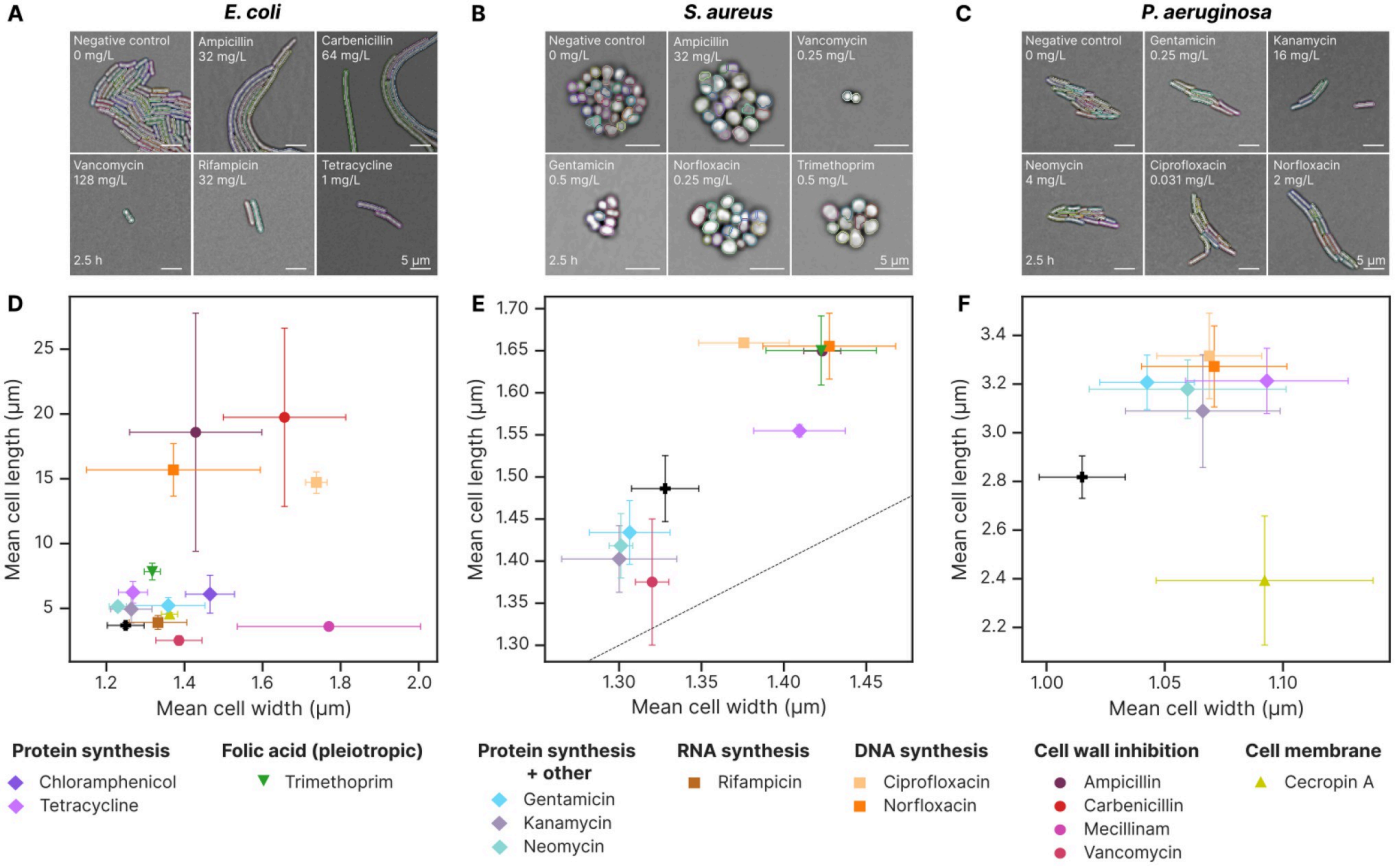
All antibiotics induce changes in morphology, though to varying degrees. These plots show data at the antibiotic concentration closest to IC_50_ after 2.5 hours of imaging. **A** Images illustrating the response of *E.coli* to antibiotics. All antibiotics, except vancomycin, produce an increase in cell size. See Fig 4 for images from ciprofloxacin, mecillinam and chloramphenicol. **B** Morphological responses of *S.aureus* to a sample of antibiotics. Ampicillin, gentamicin, norfloxacin, and trimethoprim increase cell area compared to the control, while vancomycin reduces cell size. **C** Morphological responses of *P.aeruginosa* to a selection of antibiotics. All antibiotics induce subtle increases in cell area. **D** Scatterplot showing how the test set of antibiotics affects the mean cell width and length for *E.coli*. Markers represent the mean and standard deviation across three or more replicates per antibiotic. Cell wall and DNA synthesis inhibitors induce the largest morphological changes. **E** Scatterplot showing the impact of cell wall and nucleic acid synthesis inhibitors on *S.aureus* morphology, with significant effects observed for all tested antibiotics. Protein synthesis inhibitors induce smaller changes, with kanamycin, neomycin, and gentamicin approaching the noise floor of the measurements. The dashed line represents *x* = *y*, where perfect spheres would fall. **F** Scatterplot showing the morphological effects of antibiotics on *P.aeruginosa*. With the exception of cecropin A, all tested antibiotics induce small but significant increases in both cell length and width.

Having established the 2.5-hour time point as the time of choice for assessing morphology, we investigated all of the remaining antibiotic-microbe combinations in Fig 5. Representative images of microcolonies for *E.coli*, *S.aureus*, and *P.aeruginosa* treated with various antibiotics are shown in Fig 5A–5C, respectively. Remarkably, all of the antibiotics tested caused noticeable morphological changes on all of the species tested, albeit with different magnitudes (Fig 5D–5F). For *E.coli*, all treatments except vancomycin resulted in cell enlargement, with the largest sizes observed for beta-lactams and ciprofloxacin (Fig 5D). In *S.aureus*, about half the antibiotics induced an increase in size and half induced a reduction in size. Except for tetracycline, protein synthesis inhibitors and vancomycin tended to produce smaller cells, while ampicillin, as well as DNA and folic acid synthesis inhibitors, caused cell enlargement (Fig 5E). The antimicrobial peptide cecropin A caused *P.aeruginosa* to reduce in length and increase in width, whereas the remaining treatments caused an increase in cell size (Fig 5F).

**Fig 6:**
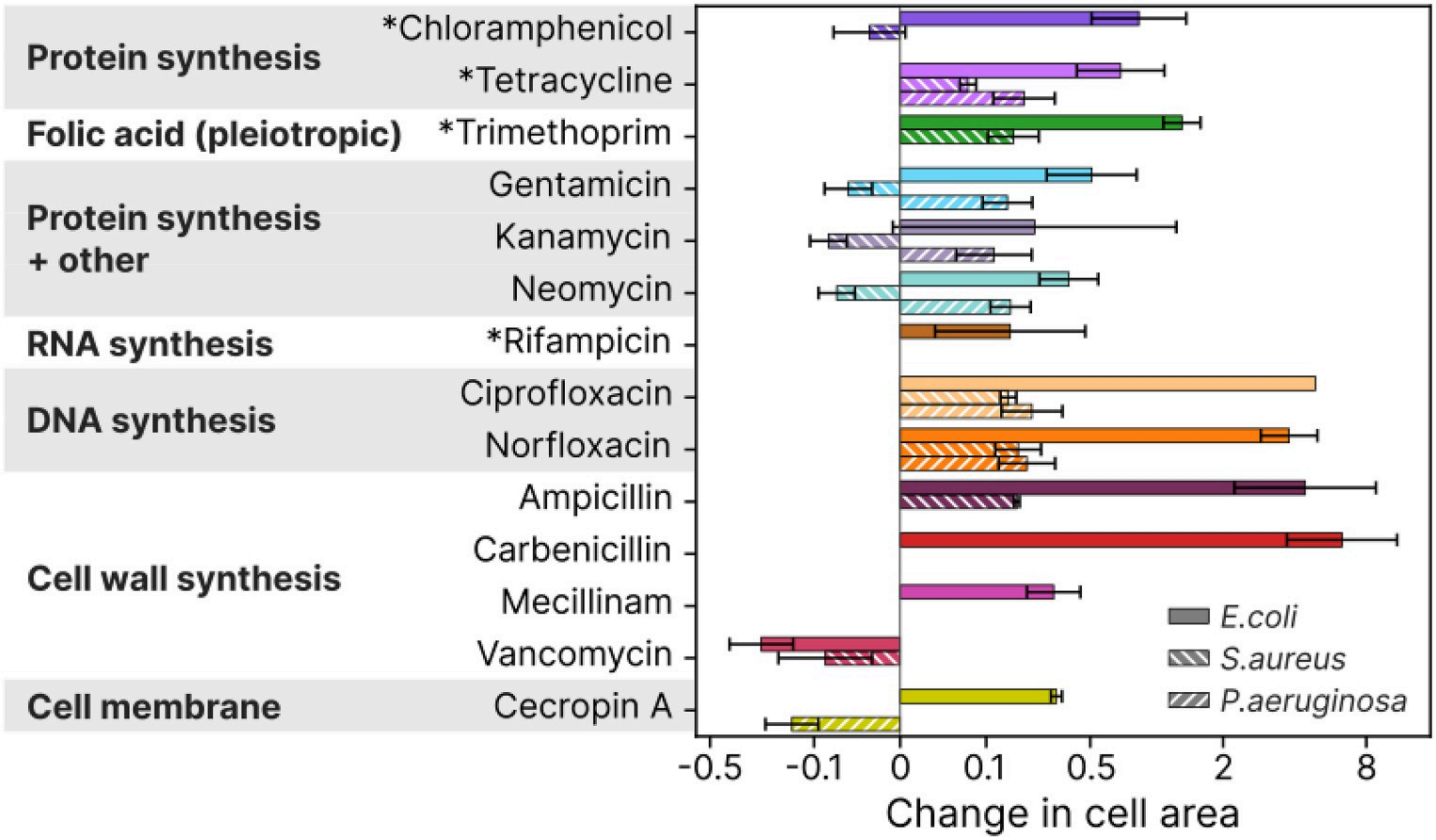
There are trends in morphology and growth characteristics between the antibiotic classes, but there are also exceptions. This plot shows how the mean cross-sectional area at IC_50_ is affected for the three species and for different antibiotics, with a symlog x-scale. The antibiotics are grouped by functional target. The bacteriostatic antibiotics are marked with *. Data is shown for *E.coli*, *S.aureus*, and *P.aeruginosa*. The error bars indicate a 95% confidence interval.

Overall, as seen in Fig 6, cell wall synthesis inhibitors generally produced the largest changes in single cell volume, whereas the protein synthesis inhibitors produced the smallest. Some antibiotics caused consistent changes across the different species (like ampicillin, norfloxacin and ciprofloxacin), while others showed significant differences. For example, several of the protein synthesis inhibitors produced smaller sizes in *S.aureus* and larger in *E.coli* and *P.aeruginosa*.

### 2.4 MOR_50_ accurately estimates the MIC using just cell morphology and drastically reduces imaging time

As shown in Fig 4, the largest morphological changes occur in specific concentration ranges, beyond which they tend to subside at least to a certain degree. This is likely because when drug concentrations are very high, the transition between growth and growth halt/death is quick and does not allow enough time for morphological changes that depend on growth (S11 Fig). For all of the antibiotics and species considered, morphological changes were closely correlated with growth inhibition (S13 Fig).

**Fig 7:**
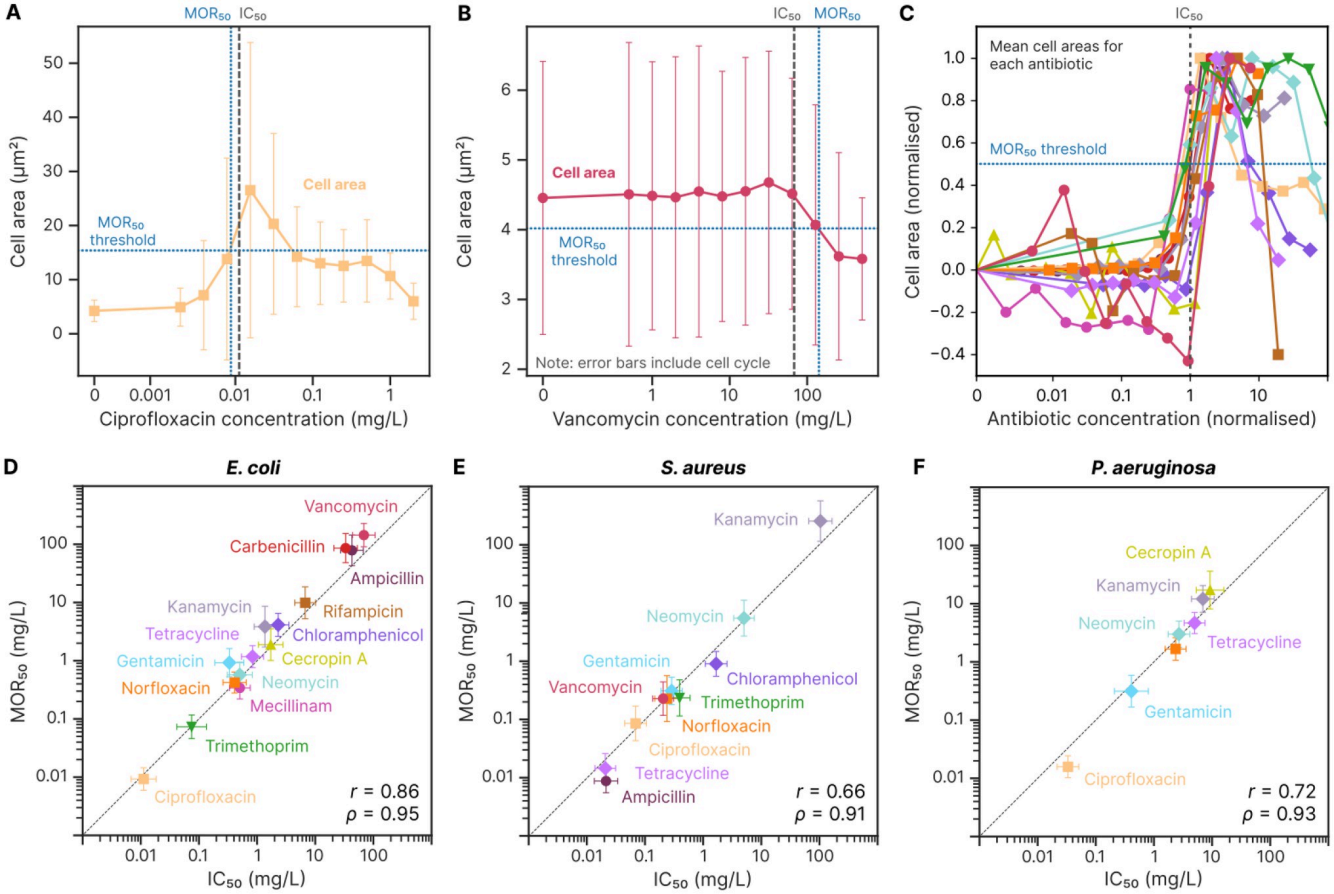
The magnitude of morphological change correlates closely with growth inhibition. *Morphological change 50* (MOR_50_) metric estimates the 50% inhibitory concentration (IC_50_) of an antibiotic from single-cell morphology data after 2.5 hours of antibiotic exposure. **A** Ciprofloxacin concentration vs cross-sectional cell area for *E.coli*. Markers show mean ± SD across repeats, capturing natural cell size variation due to the cell cycle. The MOR_50_ threshold (horizontal line) is halfway between mean areas at no antibiotic and maximum change. The MOR_50_ concentration (blue line) closely aligns with IC_50_ (grey line). Data from four biological repeats. **B** Vancomycin concentration vs cell area for *E.coli*. Markers show mean ± SD across repeats. The MOR_50_ concentration is where the curve crosses the threshold from above, aligning with IC_50_. Data from six biological repeats. **C** Antibiotic concentration (normalized to IC_50_) vs cell area (normalized so that mean area without antibiotics is zero, and mean area at the concentration of max area change is one) for all antibiotics in our test set applied to *E.coli*. The MOR_50_ threshold is shown at 50% change, and the MOR_50_ concentrations are where the area curves first cross this threshold. Morphology change varies between antibiotics, with noise evident in antibiotics inducing small changes. Each line consists of data from three or more biological repeats. **D-F** Scatterplots of IC_50_ vs MOR_50_ for *E.coli*, *S.aureus*, and *P.aeruginosa* show strong correlations (Pearson *r*, Spearman *ρ*). Dashed line: *y* = *x*. Markers: mean ± SD between repeats, with 3 or more repeats per condition.

Motivated by the correlation between morphology change and MIC, we developed a simple heuristic metric to estimate the IC_50_ (where the growth rate is inhibited by 50%) exclusively from the morphological information acquired at a single time point. *Morphological change 50* (MOR_50_) is defined as the lowest antibiotic concentration that induces a single-cell morphological change which exceeds 50% of the largest morphological change induced at any concentration of that antibiotic. Overall, cell area has proven to be the most reliable metric for assessing morphology, and it is the only parameter we use in this study for MOR_50_ determination. However, for some antibiotics, cell width or length may provide a higher signal-to-noise ratio, depending on the predominant morphological changes they induce. As an example of how to compute MOR_50_, Fig 7A shows ciprofloxacin concentration vs cell area after 2.5 hours of incubation with the antibiotic. The largest magnitude change is positive, so the MOR_50_ threshold is placed halfway between the control area at 4.2 mg L^−1^ and max area at 26 mg L^−1^, and the MOR_50_ concentration is determined as the concentration where the mean cell area curve intercepts this line. For vancomycin in Fig 7B, there is an overall larger reduction in area. Again, we place a threshold halfway between the control area and the maximum area change (which is now below the control area), and the MOR_50_ is determined as the concentration where the mean cell area curve intercepts this line. This process is shown for all combinations of tested antibiotics and species in S14 Fig. By normalizing antibiotic concentration to the IC_50_ from the growth rate Hill fit and normalizing cell area to range between zero and one, Fig 7C shows how well this works across the different antibiotics for *E.coli*. Similar plots for *S.aureus* and *P.aeruginosa* are presented in S15 Fig, along with non-normalized area plots for all three species.

We found that using cell area data from the 2.5-hour time point produces the best correlation between MOR_50_ and IC_50_ for all species (S16 Fig). At this time, we observed a precise correspondence across the three microbial species (Fig 7D–7F). A tabulated summary of the data is shown in Table 1. The geometric fold difference between the two methods is 0.6±0.5 (mean ± SD, base 2) across all tested antibiotics/species combinations. This indicates that, on average, MOR50_50_ values are within a factor of 2^0.6^ ≈ 1.5 from IC_50_.

**Table 1:** Showing average MIC values obtained through growth rate as outlined in [32] (IC_90_), and single-cell morphology using the MOR_50_ metric. All MIC values are reported in mg L^−1^. Values are reported as mean and standard deviation between biological repeats. The combinations of species and antibiotics marked with “-” were not tested. We note that our strain of *S.aureus* carries a plasmid encoding for Kanamcycin resistance.

Antibiotic	*E.coli*		*P.aeruginosa*		*S.aureus*	
	IC_50_	MOR_50_	IC_50_	MOR_50_	IC_50_	MOR_50_
Ampicillin	40 ± 10	80 ± 60	-	-	0.021 ± 0.008	0.009 ± 0.003
Carbenicillin	30 ± 10	90 ± 60	-	-	-	-
Cecropin A	1.7 ± 0.8	2 ± 2	8 ± 5	6 ± 6	-	-
Chloramphenicol	2.3 ± 0.4	4 ± 1	-	-	1.7 ± 0.4	0.9 ± 0.4
Ciprofloxacin	0.011 ± 0.005	0.009 ± 0.003	0.033 ± 0.007	0.016 ± 0.004	0.07 ± 0.02	0.09 ± 0.09
Gentamicin	0.3 ± 0.2	0.9 ± 0.6	0.4 ± 0.4	0.3 ± 0.2	0.29 ± 0.06	0.3 ± 0.2
Kanamycin	1.4 ± 0.3	4 ± 5	7 ± 2	12 ± 6	100 ± 30	300 ± 300
Mecillinam	0.51 ± 0.05	0.34 ± 0.09	-	-	-	-
Neomycin	0.5 ± 0.2	0.6 ± 0.3	2.7 ± 0.6	3 ± 1	5.0 ± 0.1	5 ± 6
Norfloxacin	0.4 ± 0.1	0.42 ± 0.06	2.4 ± 0.3	1.7 ± 0.5	0.2 ± 0.1	0.2 ± 0.4
Rifampicin	6.7 ± 0.6	10 ± 8	-	-	-	-
Tetracycline	0.8 ± 0.2	1.2 ± 0.3	5.0 ± 0.6	4.7 ± 0.7	0.021 ± 0.002	0.022 ± 0.008
Trimethoprim	0.09 ± 0.03	0.07 ± 0.03	-	-	0.39 ± 0.05	0.2 ± 0.2
Vancomycin	70 ± 20	140 ± 50	-	-	0.21 ± 0.03	0.2 ± 0.2

To compare our MOR_50_ metric with MIC from broth microdilution (MIC_BM_), we found the mean difference between IC_50_ and IC_90_ based on the MAP data is 2.4±1.2 (mean ± SD). This can then be used as a modification factor to relate MOR_50_ to MIC, which produces a fold difference between MIC_BM_ and MIC_MOR50_ of 1.3±0.9 (S17 Fig). The small amount of information required for the MOR_50_ makes it a powerful tool for MIC estimation as it minimizes imaging time. In addition, because it relies on changes rather than on absolute morphologies, the measure is completely agnostic to species identity and can be deployed without knowing the species tested beforehand. Using MOR_50_ and the MAP platform, we are able to determine the MIC of 8 antibiotics for a given biological sample in as little as 8 minutes of imaging after 2.5 hours of incubation when seeding from bacteria in the exponential growth phase.

We also attempted to correlate minimum bactericidal concentration (MBC) with the different ways of measuring MIC on the MAP platform (S18 Fig). As expected, where at all achievable, cell death of the full population required higher antibiotic doses, and we observed a closer correlation with MIC values obtained from the broth microdilution control experiments (MIC_BM_).

## 3 Discussion

The effects of antibiotics on the cell at a system level are only partly understood. Here, we used MAP, a high-throughput imaging platform we recently developed, to investigate the system-level effects of 14 antibiotics on the growth and morphology of three clinically relevant bacterial species. Specifically, we demonstrated that bacterial microcolonies grow exponentially at a certain, consistently heterogeneous rate. Upon antibiotic treatment, as the dose approaches the MIC, things change. Cell wall synthesis inhibitors cause the largest increase in PGRH, followed by cell membrane synthesis inhibitors, DNA synthesis inhibitors, RNA synthesis inhibitors, protein synthesis inhibitors with other additional targets, and folic acid synthesis inhibitors. Finally, protein synthesis inhibitors affect PRGH very little. Based on these observations, we speculate that bacteria’s growth rate heterogeneity in response to an antibiotic depends on the functional distance between antibiotic targets and cellular growth rate control. The active ribosomal pool has been recognised to modulate growth rate in a number of studies [36–40], and antibiotics that influence it directly benefit from a fast track towards growth impairment. Antibiotics that do not use this mechanism of action are functionally further away from growth rate control. Thus, their impact on growth rate depends on the inherent robustness to perturbations of each cell as a system. While at this stage the repercussions of such phenomenon are unclear, heterogeneity has frequently been linked to persistence and treatment survival. Further studies will show whether heterogeneity-boosting antibiotics may enhance persister production, for example, when drug cocktails are administered. Lastly, the validity of these observations for *S.aureus* and *P.aeruginosa* further to *E.coli* suggests that the same growth laws may apply universally, even in species that have been less extensively studied from a systems-level physiological perspective.

Morphology is the other variable controlled at the system level that we were able to assay in this work. As demonstrated by others (primarily using data from balanced growth of *E.coli*), many antibiotics alter bacterial morphology. Here, we have shown that this is true for all of the 31 antibiotic-bacterium combinations, regardless of whether the cells have a rod or coccoid shape or the antibiotic’s mechanism of action, although these factors do influence the magnitude of the effect. We have shown that to emerge, defects require growth in all cases, although we did not investigate antimicrobials such as Gramicidin S, that directly pierce the cell envelope [41]. We could confirm the well-established mechanism that leads to morphological alterations for some of the antibiotics tested, such as ciprofloxacin or beta-lactams. In other cases, the results were surprising. For example, in a rich medium such as the one in our pads, *E.coli* cells in balanced growth that are treated with chloramphenicol are expected to shrink [4] while we observed them swell. While we do not know the reason for this discrepancy, we assume that this emerges from the differences in growth conditions.

We have also demonstrated that single-cell morphology alone can accurately estimate the minimum inhibitory concentration (MIC) of antibiotics, providing a rapid and cost-effective method for single-cell imaging research and antibiotic susceptibility testing (AST). The morphology-based metric we introduced, *morphological change 50* (MOR_50_), correlates strongly with growth inhibition IC_50_. Furthermore, MIC estimation based on MOR_50_ (MIC_MOR50_) aligns closely with MIC values obtained using traditional broth microdilution (MIC_BM_) and growth-rate-based MIC determination on the MAP platform (IC_90_) across all tested antibiotic classes. Notably, MIC_MOR50_ enables MIC estimation within 2.5 hours of incubation with only a few minutes of imaging time, significantly reducing the time and cost of AST. Testing with clinical isolates will confirm if MOR_50_ can be applied generally in a clinical context. Clinical samples will need to be understood with particular attention to added layers of biological complexity, for example one can expect phenomena such as heterogeneity in the length of the lag phase, which might condition the degree of morphological changes. There may of course also be technical challenges in segmenting the cells, depending on the nature of the clinical isolates and the degree of their pre-processing. This advancement has the potential to transform AST by facilitating the quick selection of effective antibiotics against the backdrop of rising antimicrobial resistance.

While genotypic methods have advanced rapidly, phenotype-based assays remain the gold standard for AST due to their direct link to functional outcomes, although these methods often face challenges related to cost and time. Recent efforts, such as those by Choi *et al*. [42] and Baltekin *et al*. [43], have laid important groundwork in rapid AST methods. However, our MAP platform, coupled with the MOR_50_ metric, surpasses previous approaches in both throughput and affordability, as well as robustness across species and antibiotic classes. Unlike micro-injection mould-dependent systems, MAPs are inexpensive to fabricate and deliver exceptionally high-quality microscopy images. Similar to earlier fast AST methods, our approach can produce MIC results based on growth rate within 2.5 hours, but by leveraging morphology, results can be obtained after only 8 minutes of imaging per MAP. Freeing up instrument time gives the potential to process up to 100 MAPs on a single microscope per day, equating to testing 800 antibiotic-bacteria combinations or approximately 10, 000 individual pads daily. Such throughput drastically reduces the effective cost per sample. Future integration with automated incubation and loading platforms could further enhance throughput and ease of use, making MAP a practical and scalable tool for clinical implementation.

## 4 Materials and methods

### 4.1 Sample preparation

Experiments were conducted with strains of *E.coli*, *P.aeruginosa* and *S.aureus* (Table 2) in Luria-Bertani (LB) Broth (ThermoFisher, 10855001, with 10 g peptone, 5 g yeast extract, 5 g sodium chloride per 1 L media) and Mueller Hinton (MH) Broth (Sigma-Aldrich, 70192, with 2.0 g beef infusion solids, 17.5 g casein hydrolysate and 1.5 g starch per 1 L of media). Our *S.aureus* SH1000-pMV158GFP carries a KanR cassette S2 Fig. All of the bacteria morphology data presented in this work was collected using the Multipad Agarose Platform (MAP) as described in [32]. Some of the *E.coli* growth rate datasets used in this study (namely those concerning the antibiotics ampicillin, carbenicillin, chloramphenicol, ciprofloxacin, mecillinam, rifampicin, tetracycline and vancomycin) were also used in our previous work [32]. This data is used for the plots in Figs 2 and 3, as well as the relevant subfigures in S2–S9 Figs. The MAP platforms were prepared in batches of three, with antibiotic dilution series as outlined in S19 Fig. Pre-cultures were grown overnight, diluted 500x into fresh media, and allowed to resume exponential growth for at least three generations. These seed cultures were then diluted to a 600 nm optical density (OD_600_) of 0.03, and aliquots of 1.5 μL were placed on the surface of each MAP pad. To avoid damaging the pads, the sample was ejected to form a droplet at the end of the pipette tips (Fisherbrand SureOne Aerosol Barrier Pipette Tips 10 μL, 02–707-442), which was then gently touched to the surface of each pad. The pads were dried for 5 to 10 minutes before peeling the protective film on the upper adhesive sheet and sealing the pads with a single glass slide that covers all the pads (UQG Optics, GPD-1577, dimensions 110 × 74 × 0.17 mm).

**Table 2:** Outlining the strain, growth media and analysis statistics for the different bacteria species used for these experiments. The growth media was Luria-Bertani (LB) Broth and Mueller Hinton (MH) Broth. We started using LB Broth with *E.coli*, but switched to MH Broth for the later experiments with *P.aeruginosa* and *S.aureus* as we wanted to test with this media as well, given it is recommended for use with AST by EUCAST [44]. The same growth media was used for precultures and preparing pads on the MAP platform. Pad count, microcolony count and bacteria count are reported from a single timepoint around 2.5 hours after imaging was started, meaning each microcolony and cell counted are unique. For the analysis, data from a few consecutive time steps is typically used.

Species	Strain	Growth media	Pad count	Microcolony count	Bacteria count
*E.coli*	K-12, MG1655	LB Broth	707	6 332	541 978
*P.aeruginosa*	PA01-CFP [45]	MH Broth	503	7 297	333 314
*S.aureus*	SH1000-pMV158GFP [46]	MH Broth	491	6 581	186 662

### 4.2 Timelapse microscopy

The MAP platform is imaged at 37°C on a custom-built, open-frame inverted microscope for 5 hours. One field of view (FOV) is imaged per pad, using a looping script to capture all the images automatically. An LED focusing system is used to keep the sample roughly in focus automatically. To account for possible misalignment between the camera’s focal plane and the imaging plane of individual pads (which can exhibit significant tilts in relation to each other on the same MAP), a z-stack of images is captured for each FOV. We typically capture about ten frames per stack with 0.4 μm step size. This approach ensures that each microcolony is captured at its optimal focus in one of the frames, despite the spatial variations in the imaging planes across different pads. For these experiments, we chose the FOVs manually at the start of the time-lapse. However, selecting FOVs could be done in an automated fashion to give a fully automated imaging workflow. Our workflow enables the rapid high-throughput imaging of a large number of microcolonies and cells; their numbers for this study are given in Table 2.

Imaging was performed with brightfield illumination using a Nikon 40x CFI Plain Flour air objective with a numerical aperture of 0.75. The camera was a Teledyne FLIR BFS-U3–70S7M-C with a 7.1 MP Sony IMX428 monochrome image sensor. All images were captured at 3208 × 2200 pixels, resulting in an effective resolution of 0.112 μm/pixel. The temporal resolution of the datasets is about 8 minutes, limited by the time it takes for the microscope to image all 96 pads of the MAP.

### 4.3 Antibiotics

The antibiotics were mixed into separate dilution series before being transferred to the pads of the MAP. They were sourced as follows: ampicillin (Sigma-Aldrich 10835242001), carbenicillin (Sigma-Aldrich C1389), cecropin A (Bachem AG, 4030488.1000), ciprofloxacin (Sigma-Aldrich 17850), chloramphenicol (Sigma-Aldrich C0378), gentamycin (Sigma-Aldrich G1397), kanamycin (Sigma-Aldrich K4000), mecillinam (Sigma-Aldrich 33447), neomycin (Sigma-Aldrich N1142), norfloxacin (Sigma-Aldrich N1142), tetracycline (Sigma-Aldrich T3258), trimethoprim (Sigma-Aldrich T7883), rifampicin (Sigma-Aldrich 557303) and vancomycin (Sigma-Aldrich V2002).

Stock solutions were prepared by dissolving the antibiotics in 10 mL of milliQ water (ampicillin, carbenicillin, cecropin A, ciprofloxacin, gentamycin, kanamycin, mecillinam, neomycin, tetracycline, vancomycin), 100% methanol (rifampicin, trimethoprim), or 95% ethanol (chloramphenicol, norfloxacin). These were stored at −20°C until use. Some antibiotics were subjected to ultrasonication to aid in their dissolution process.

Antibiotics were homogeneously mixed with the agarose and growth medium at 60°C prior to preparing the dilution series. Subsequently, the mixture was dispensed onto the MAP platform using an Opentrons OT2 pipetting robot [47], where it solidified to form the small assay pads.

### 4.4 Image processing

The images are analysed using the processing pipeline available in our open-source Python package *PadAnalyser*. The processing is outlined here:

**Z-stack projection** is performed to reduce the z-stack into a single in-focus frame. We first try plane-based projection, which models the global tilt in focus using a plane and performs projection based on this plane. Steps are: 1) compute the Laplacian for each frame, 2) divide the image into regions and calculate preferred focus indices for each region based on intensity peaks, 3) fit a plane to the preferred focus indices using least squares 4) create a 3D mask based on the plane equation for linear interpolation between z-stack frames, and 5) combine frames according to the mask to generate the final projected image. The main advantage of this approach is that it provides a continuous projection, preserving focus consistency across the field of view. However, sometimes this fails. Common reasons include backlash in the z-stage of the microscope, leading the in-focus peak to spread across frames. In these cases, a fallback tile-based projection algorithm is used, which divides the image into smaller tiles (regions) and analyzes each tile independently. The steps are: 1) compute the Laplacian for each frame to emphasize focus areas, 2) square the Laplacian values to amplify differences in focus quality, 3) calculate a focus score for each tile across the stack, 4) use a weighted convolution kernel to enhance spatial continuity in scoring, and 5) for each tile, select the frame with the highest focus score and add its contribution to the output using a weighted window kernel.
**Pre-processing** is performed on each projected frame by clipping the brightest and darkest pixels, applying a Gaussian blur with 3x3 kernel, and normalizing the result to integers in the range 0 to 255. These frames are stored as 8-bit grayscale images.
**Colony segmentation** is done using the Scikit Image Canny Edge Detector [48] with *σ* = 1 to identify edges in the input image. Morphological closing is then applied with a circular kernel of size 7 × 7 for two iterations, filling small gaps in the detected edges and creating a binary mask representing potential colonies. Masks touching the frame boundary (within 20 pixels) are removed using border-clearing techniques, and regions smaller than a minimum area (species-dependent) are excluded to eliminate debris. Finally, masks are analyzed for focus and shape consistency by evaluating their Laplacian intensity profiles as a function of distance from the mask edge. Valid colonies exhibit a positive peak within 5 pixels and a negative peak within 10 pixels from the edge. Masks failing this criterion are discarded. This approach is identical to that described in [32].
**Single-cell segmentation** uses the Laplacian of the Gaussian (LoG) of the brightfield image, before applying a simple threshold to binarize the image. The binary image is filtered using the colony masks, and converted to contours with OpenCV [49]. Very small contours are removed as they correspond to debris and optical artefacts, and the remaining contours are split based on an outline curvature metric and point separation to make sure neighbouring bacteria get individual masks S20 Fig. Finally, the contours are dilated to represent the true cell areas better. See S21 Fig for details.
**Frame alignment** is done based on colony segmentation masks. The centroids of segmented colonies are then used to align consecutive frames, compensating for thermal expansion and stage drift.
**Linking colonies over time** is done to track colony growth rates. Colonies from the different time steps of a FOV are linked and assigned an identity. Colony growth rates are computed based on the rate of change in colony area over time using the *gaussianprocessderivatives* python package [50].
**Extracting statistics** from cell and colony masks is key to processing and visualising these large datasets. Colony and cell areas are computed using the OpenCV *cv2.contourArea* function [49]. See Section 4.6 for more information on how cell lengths and widths were computed. The colony and single-cell statistics are bundled into two data frames per experiment: one for the colony statistics (with single-cell statistics linked to each relevant colony) and one for the single-cell statistics.
**Debug output** is important to verify accuracy. Two output video files are produced for each field of view with segmentation masks for colony outlines and single cells drawn in clear colours so the user can validate that the algorithms are working correctly, see [51].

For the single-cell segmentation, we could not find one set of parameters that would accurately segment the three different species, so we determined four key segmentation parameters we could alter to tune the algorithm on a species-by-species basis. These are outlined in S1 Table along with the values used. These parameters are chosen to ensure the segmentation pipeline is robust to the antibiotics-induced changes in cell morphology observed for each species.

### 4.5 Single cell segmentation benchmarking

The single-cell segmentation code was compared to manually annotated images to ensure accuracy. We manually labelled frames from a range of different time points and antibiotic concentrations for all species to ensure the approach is robust to different cell morphologies, colony sizes and imaging conditions. The comparison results are outlined in S2 Table, highlighting three main metrics. *Intersection over union* (IoU) outlining the overall area overlap, *mean cell area error* as a ratio between the mean cell areas, and *mean errors per cell* report the number of segmentation errors per cell in the labelled image [52].

### 4.6 Extracting single cell statistics

In *PadAnalyser*, we compute the area (*A*) of rod-shaped bacteria using the *cv2.contourArea* function from the OpenCV library [49], which accurately calculates the area of the mask representing the bacteria. For width estimation, we utilized the Euclidean distance transform (EDT), taking twice the maximum value of the EDT as the representative width (*w*) of the bacteria. This approach effectively captures the diameter of the widest part of the bacteria. The length (*l*) was then computed using the formula $l=\frac{A}{w}+w(1-\frac{\pi }{4})$. This formula derives from the relationship between the area and width of a spherocylinder. The term $w(1-\frac{\pi }{4})$ serves as an adjustment to the basic area-to-width ratio, compensating for the fact that the bacteria have round caps and providing a more accurate measure of length for rod-shaped and potentially curved bacteria.

## Supporting information

S1 Table An overview of the parameters used to tailor the segmentation pipeline to the different species of bacteria. Sigma sets the strength of the Gaussian blur used to smooth the initial z-stack projected image before computing the Laplacian. The min mask size filter sets the minimum size of a mask to be considered a cell. Threshold sets the threshold value used to binarise the image. The split factor controls how aggressively the masks are split.
(PDF)

S2 Table Comparison of how accurate the PadAnalyser segmentation code is based on the metrics *intersection over union* (IoU), *mean cell area error*, and *mean errors per cell* across different species. The data includes total frame count and cell count to indicate the extent of the comparison. The row *manually annotated* compares two frames that both have been manually annotated to assess best-case metrics.
(PDF)

S3 Table EUCAST MIC concentrations are based on tabulated confidence intervals from *mic.eucast.org*. Where data is available but the confidence interval is not reported, the mean and standard deviation of available data are used to estimate the confidence interval.
(PDF)

S1 Fig Showing how the colony area growth rate changes over time as the bacteria area is exposed to various concentrations of the antibiotics. Higher antibiotic concentrations lead to growth inhibition after varying time delays. A lighter hue corresponds with a lower antibiotic concentration. The lines show mean values with the shaded region indicating standard deviation between repeats. Each plot shows data from three or more repeats. The growth rates are binned to the nearest 30-minute interval.
(PDF)

S2 Fig Showing how growth varies with antibiotic concentration between 2 and 3 hours of incubation on the MAP. The markers show mean and standard deviation between repeats. A Hill fit is performed for each antibiotic, with the vertical lines showing IC_10_ and IC_90_ concentrations, where the growth rate is inhibited by 10% and 90%, respectively. The plots for *E.coli* with tetracycline, rifampicin, and ampicillin are also presented in Fig 2 and are included here for completeness.
(PDF)

S3 Fig Growth rate dependence on antibiotic concentration for *E.coli*. Each repeat is plotted individually, and Hill fits performed for each repeat. Data used is for the time between 2 and 3 hours of growth. The vertical lines show the IC_90_ concentrations where growth is inhibited by 90%, which we define as MIC.
(PDF)

S4 FigBenchmarking MIC measurements using growth rate after 2.5 hours on the MAP (IC_90_) against broth microdilution (MIC_BM_).The OD600 vs time curves from the broth microdilution experiment are shown in S22 Fig. The antibiotic type is indicated by hue. Each marker shows the mean and standard deviation between repeats, and the line *x* = *y* is shown. The fold difference plot shows how well MIC from growth rate (IC_90_) compares with MIC from broth microdilution (MIC_BM_). The fold difference is computed as ${\text{log}}_{2}\frac{{\text{IC}}_{90}}{{\text{MIC}}_{BM}}$, such that a value of for example 1 means IC_90_ is 2 times higher than MIC_BM_, and -2 means 4 times lower. The bars show the mean and standard deviation between repeats.(PDF)

S5 Fig Showing how MIC measurements compare between EUCAST tabulated data from S3 Fig, measurements based on growth rate measured on MAP platform, and MOR_50_ metric on MAP platform for *E.coli*, *S.aureus* and *P.aeruginosa*. (PDF)

S6 Fig Showing the growth rate heterogeneity (PGRH) at different antibiotic concentrations. PGRH shows the variation in growth rate between colonies in the same growth environment, which we define as the standard deviation of colony growth rate for one pad at one point in time. This value is averaged between 1 to 2.5 hours of incubation on the MAP. The markers show mean and standard deviation between repeats. There are at least three repeats per antibiotic/species combination. The white marker in each plot shows the concentrations selected for subsequent PGRH analysis. The vertical lines correspond to the IC_10_ and IC_90_ concentrations where the growth rate is inhibited by 10% and 90%, respectively. We see that for some antibiotics, high concentrations produce a much higher PGRH and that this change is linked to the MIC. For others, like chloramphenicol, there is a drop in PGRH linked to the use of the antibiotic, also around the MIC. The plots for *E.coli* with tetracycline, rifampicin, and ampicillin are also presented in Fig 2 and are included here for completeness.
(PDF)

S7 Fig Summarising growth rate heterogeneity (PGRH) and Hill fit exponent (n) for all the tested antibiotic/species combinations. PGRH has been normalised to the antibiotic-free growth rate for each species in the same time window. Each bar represents the mean and standard deviation between repeats, based on a least three repeats per condition. Each antibiotic has a dedicated hue, and each species has a separate shading.
(PDF)

S8 Fig Summarising population growth rate heterogeneity (PGRH) for the different antibiotic categories. The bars show the mean and standard deviation between repeats and antibiotics belonging to the relevant categories. PGRH has been normalised to the antibiotic-free growth rate for each species in the same time window.
(PDF)

S9 Fig Scatterplots show how population growth rate heterogeneity (PGRH) correlates with Hill fit exponent (n) for the three species separately. Data is shown for PGRH at the peak in the vicinity of the MIC (left) as well as at the concentration closest to the MIC (right). These two correspond well generally, but notable exceptions exist, including for the DNA synthesis inhibitors where PGRH spikes at concentrations significantly higher than the MIC. Each marker shows the mean and standard deviation between repeats for a given antibiotic/species combination (three or more repeats per condition). Each antibiotic is allocated a separate hue.
(PDF)

S10 Fig Showing how growth rate and population growth rate heterogeneity (PGRH) varies between *E.coli* wildtype WT and Δ*tolC* strains for two antibiotics, ciprofloxacin and tetracycline. The mutant has a lower MIC for both ciprofloxacin and tetracycline, but there is no significant difference in PGRH between the two. **AB** Growth rate vs antibiotic concentration plots for ciprofloxacin and tetracycline. The points show the mean and standard deviation between repeats. There are three repeats per condition. **CD** Normalizing the antibtioic concentration to IC_50_ shows how the susecptibility curve differes between the strains. A normalized concentration of 0 corresponds to the IC_50_, while 1 corresponds to IC_90_. The vertical lines show the IC_10_ and IC_90_ concentrations, respectively. **EF** shows population growth rate heterogeneity (PGRH) with antibiotic concentration normalised as in CD.
(PDF)

S11 Fig Showing how cell area changes over time when exposed to various concentrations of the antibiotics. Initially, all bacteria have the same morphology as they are introduced to the pads with antibiotics. As time progresses, many antibiotics cause a significant change in morphology for some concentrations. Generally, the largest differences are apparent after 2 hours of growth. Each line represents the mean area per cell for a given antibiotic concentration, and the shaded area represents the standard deviation with data originating from three or more repeats. Darker colours correspond to higher antibiotic concentration, and data points are binned to the nearest 30 minutes. The plots for *E.coli* with ciprofloxacin, mecillinam and chloramphenicol are also presented in Fig 4 and are included here for completeness.
(PDF)

S12 Fig Boxplots showing how cell area, length and width appear after 2.5 hours of incubation on the MAP. The boxplots show the median, quartiles, and whiskers show the 1.5 times the interquartile range. The plots for *E.coli* with ciprofloxacin, mecillinam and chloramphenicol are also presented in Fig 4 and are included here for completeness.
(PDF)

S13 Fig Showing how the mean cell areas change with antibiotic concentration based on data from 2.5 hours of incubation on the MAP. The points represent the mean and standard deviation of cell areas between repeats. The vertical lines represent IC_10_ and IC_90_, respectively.
(PDF)

S14 Fig Showing how MOR_50_ is calculated for all antibiotic/species combinations. The points represent the mean and standard deviation in cell area across repeats. The size of the error bars is large, as they capture the variation in cell size caused by the cell cycle. The MOR_50_ threshold is halfway between the area with no antibiotic present and the maximum area change and is shown as a horizontal line. The cell MIC is where the area curve first crosses this threshold. This corresponds very closely with the IC_50_ point as determined by the growth rate, represented by the vertical grey line. The plots for *E.coli* with ciprofloxacin and vancomycin are also presented in Fig 7 and are included here for completeness.
(PDF)

S15 Fig Showing how cell area changes depend on antibiotic concentration for *E.coli*, *S.aureus* and *P.aeruginosa*. The plot for *E.coli* is also shown in the main text but repeated here for completeness. For each species, curves are shown for all the tested antibiotics after 2.5 hours of incubation with the antibiotics. The antibiotic concentrations are normalised to growth MIC, and the cell area is normalised so that the no-antibiotic area is 0 and the maximum change in cell area is 1 (which for vancomycin entails inverting the area). The MOR_50_ threshold is shown as a horizontal line at 50% change, and the cell MIC is where the area curve first crosses this threshold. All the antibiotics produce a morphology change around the growth MIC. The degree of change varies strongly between antibiotics, which is here made apparent through the noise present in the area signal for antibiotics that induces a small change in morphology. The antibiotics are categorised by action mechanisms as inhibiting cell wall synthesis, protein synthesis, and nucleic acid synthesis, in addition to a negative control where no antibiotic was used. The plot for *E.coli* with normalized cell area is also presented in Fig 7 and is included here for completeness.
(PDF)

S16 Fig Computing Pearson’s and Spearman’s rank correlation coefficients between MOR_50_ and IC_50_ for the three species. All species show the best correlation at 2.5 hours of incubation on the MAP platform.
(PDF)

S17 FigScatterplots showing how MOR_50_ compares with MIC as computed using broth microdilution control experiments.Points represent the mean and standard deviation between repeats for each antibiotic/species combination. The fold difference plot shows how well MIC from MOR_50_ (MIC_MOR50_) compares with MIC from broth microdilution (MIC_BM_). The fold difference is computed as ${\text{log}}_{2}\frac{{\text{MIC}}_{MOR50}}{{\text{MIC}}_{BM}}$, such that a value of for example 1 means MIC_MOR50_ is 2 times higher than MIC_BM_, and -2 means 4 times lower. The bars show the mean and standard deviation between repeats.(PDF)

S18 FigFold difference plots showing comparisons between MIC from broth microdilution (MIC_BM_), MIC from growth rate (IC_90_) and MIC from MOR_50_ (MIC_MOR50_), with the minimum bactericidal concentration (MBC), respectively.The fold difference is computed as ${\text{log}}_{2}\frac{\text{MIC}}{\text{MBC}}$, such that a value of for example 1 means MIC is 2 times higher than MBC, and -2 means 4 times lower. The bars show the mean and standard deviation between repeats.(PDF)

S19 Fig MAP setup used for AST where each row has a concentration gradient of a different antibiotic, making it possible to test eleven concentrations + control for eight combinations of antibiotic and bacteria at a time. (PDF)

S20 Fig An overview of how the contours are split into separate cells based on outline curvature and separation. **A** shows an example with *S.aureus*. A spline is fitted to the outline of the cells (shown in red), and its curvature is computed at each point. Positive curvature maps to where the surface curves outwards. Discarding all negative curvatures, the maximum curvature locations are found. If more than 10 points separate their position along the curve, and they are closer than the max width of the contour times the split factor, the split is performed. Using these points of maximum curvature as seed points, a local search is conducted around these points to find the pair of points that are closest to each other. **B** shows an example with *E.coli* where the recursive nature of the algorithm is highlighted.
(PDF)

S21 Fig Step-by-step view of the cell segmentation process. A sample colony is used to illustrate each step, with a zoomed-in view for each step to highlight the cell details. The colony was captured after two hours of imaging. **A** First, the z-stack projection of the brightfield image is processed to generate a binary image containing the cell masks. **B** Then, the masks are converted to contours and manipulated to produce accurate segmentation for the bacteria.
(PDF)

S22 Fig Showing broth microdilution control experiment results. Each curve shows the mean and standard deviation in OD_600_ over time for each antibiotic concentration over time. The broth microdilution MIC (MIC_BM_) is selected as the lowest concentration of antibiotic that leads to a growth rate that does not exceed the red OD_600_ threshold before the black time threshold of 15 hours. For some antibiotic/species combinations, this never happens. For this antibiotic, we have marked the species as resistant.
(PDF)
