# Supplementary material for: Antibiotics change the population growth rate heterogeneity and morphology of bacteria
Source: PLoS Pathog. 2025 Feb 5;21(2):e1012924. doi: 10.1371/journal.ppat.1012924 (PMC11835381; doi:10.1371/journal.ppat.1012924)
Supplement: S1 Table — Sigma sets the strength of the Gaussian blur used to smooth the initial z-stack projected image before computing the Laplacian. The min mask size filter sets the minimum size of a mask to be considered a cell. Threshold sets the threshold value used to binarise the image. The split factor controls how aggressively the masks are split. (PDF) [file ppat.1012924.s001.pdf]

S1 Table

Parameter	*E. coli*	*S. aureus*	*P. aeruginosa*
sigma	1.5	2.5	1
threshold	-1000	-2000	-3000
min mask size filter (px)	60	15	30
split factor	0.3	0.75	0.65
