# Supplementary material for: Antibiotics change the population growth rate heterogeneity and morphology of bacteria
Source: PLoS Pathog. 2025 Feb 5;21(2):e1012924. doi: 10.1371/journal.ppat.1012924 (PMC11835381; doi:10.1371/journal.ppat.1012924)
Supplement: S2 Table — The data includes total frame count and cell count to indicate the extent of the comparison. The row manually annotated compares two frames that both have been manually annotated to assess best-case metrics. (PDF) [file ppat.1012924.s002.pdf]

S2 Table

Species	Frame count	Cell count	IoU	Mean cell area error	Mean errors per cell
E. coli	10	2421	0.76	0.11	0.39
S. aureus	7	642	0.77	0.19	0.21
P. aeruginosa	7	2010	0.75	0.039	0.44
Manually annotated	3	369	0.77	0.0099	0.13
