# Supplementary material for: Antibiotics change the population growth rate heterogeneity and morphology of bacteria
Source: PLoS Pathog. 2025 Feb 5;21(2):e1012924. doi: 10.1371/journal.ppat.1012924 (PMC11835381; doi:10.1371/journal.ppat.1012924)
Supplement: S3 Table — Where data is available but the confidence interval is not reported, the mean and standard deviation of available data are used to estimate the confidence interval. (PDF) [file ppat.1012924.s003.pdf]

S3 Table

Antibiotic	*E. coli*	*S. aureus*	*P. aeruginosa*
Ampicillin	10 6	0.3 0.2	-
Carbenicillin	-	-	-
Chloramphenicol	12 4	12 4	-
Ciprofloxacin	0.05 0.02	1.5 0.5	0.8 0.3
Gentamycin	-	-	-
Kanamycin	20 10	30 30	500 500
Mecillinam	0.4 0.1	-	-
Neomycin	5 4	2 2	100 100
Norfloxacin	0.3 0.2	8 8	4 4
Rifampicin	-	0.012 0.004	-
Tetracycline	3 1	0.6 0.4	80 50
Trimethoprim	3 2	1.3 0.8	-
Vancomycin	-	1.5 0.5	-
