# Supplementary figures and images for: Antibiotics change the population growth rate heterogeneity and morphology of bacteria
Source: PLoS Pathog. 2025 Feb 5;21(2):e1012924. doi: 10.1371/journal.ppat.1012924 (PMC11835381; doi:10.1371/journal.ppat.1012924)

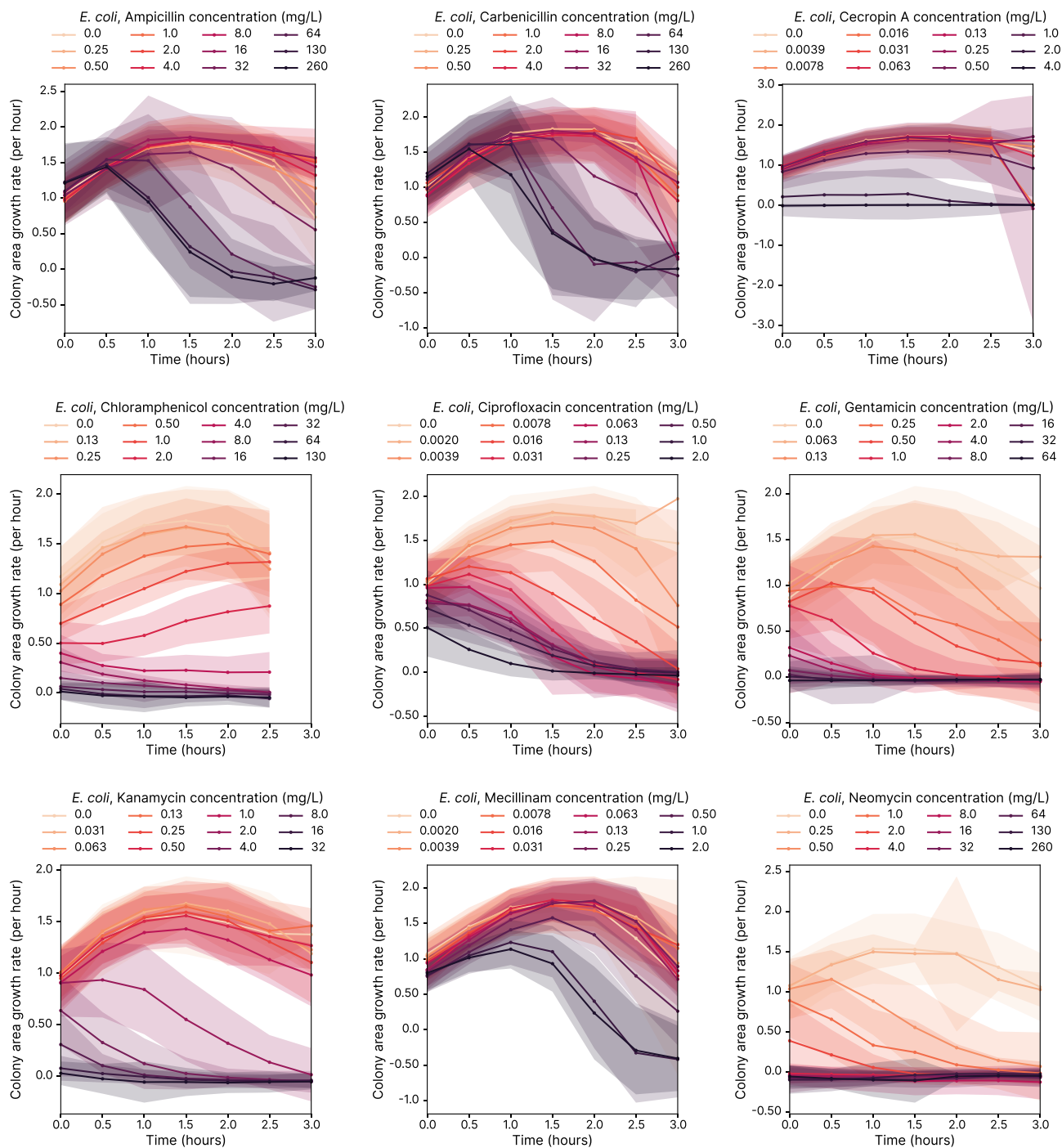

S1A Fig

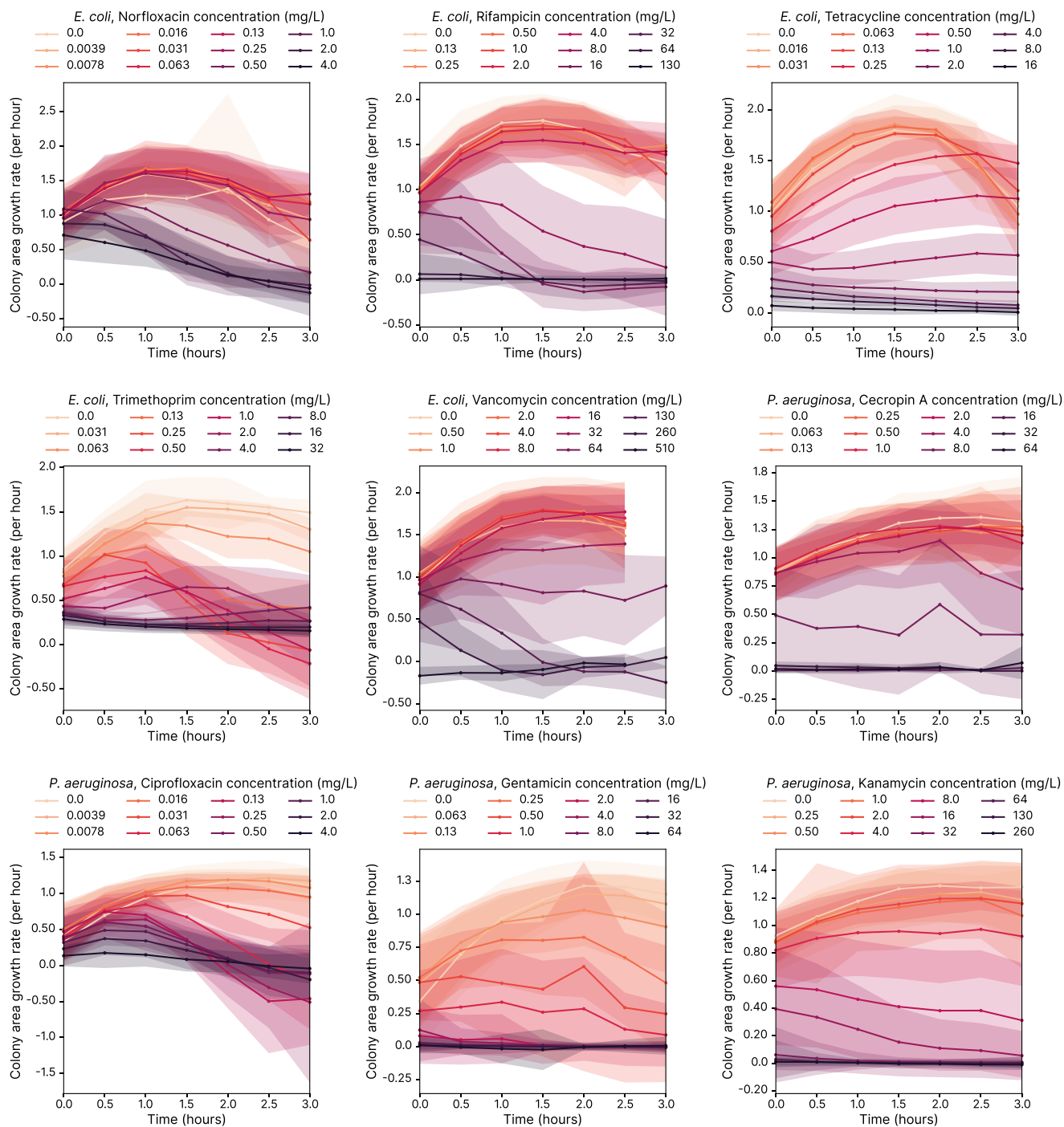

S1B Fig

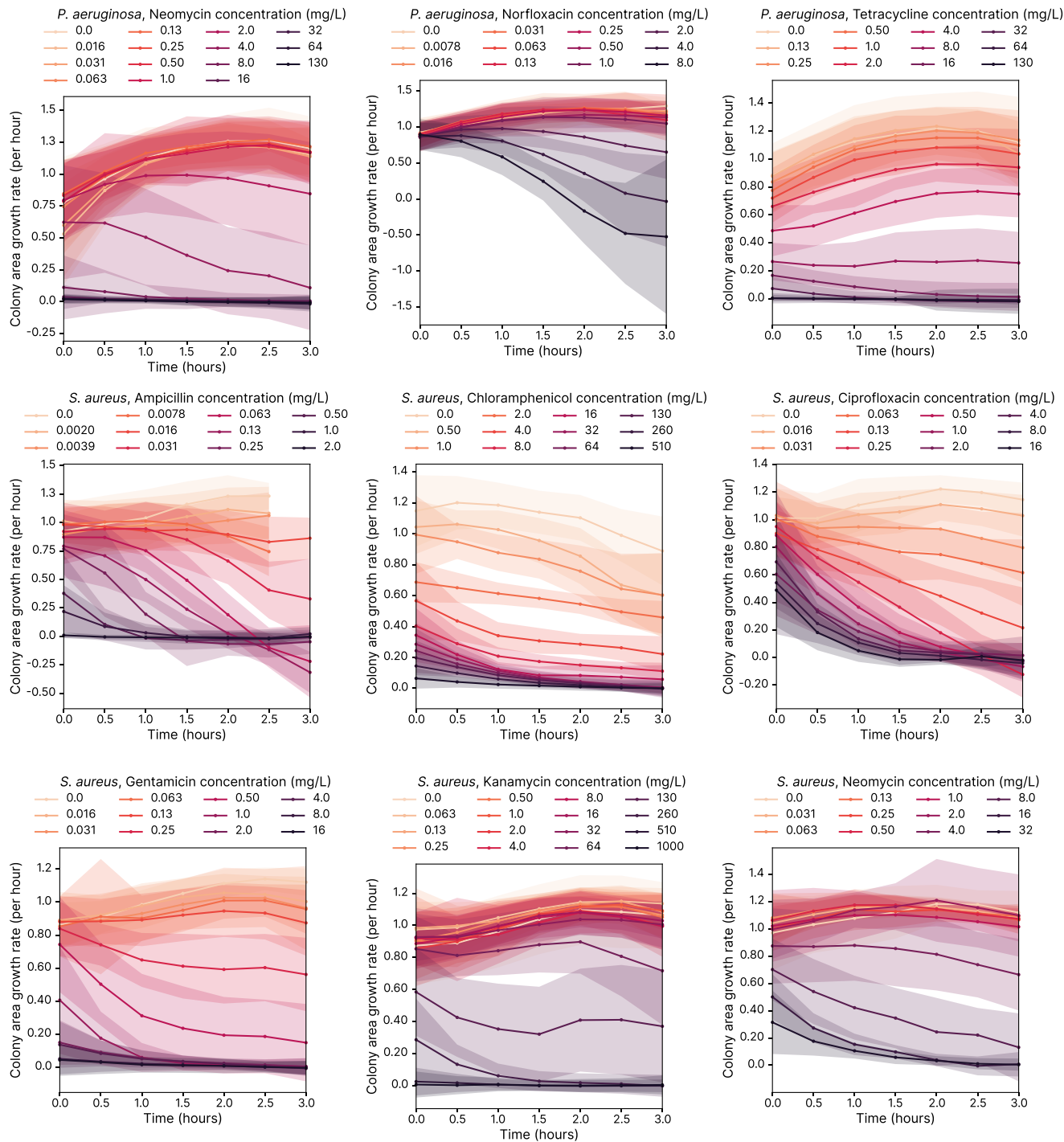

S1C Fig

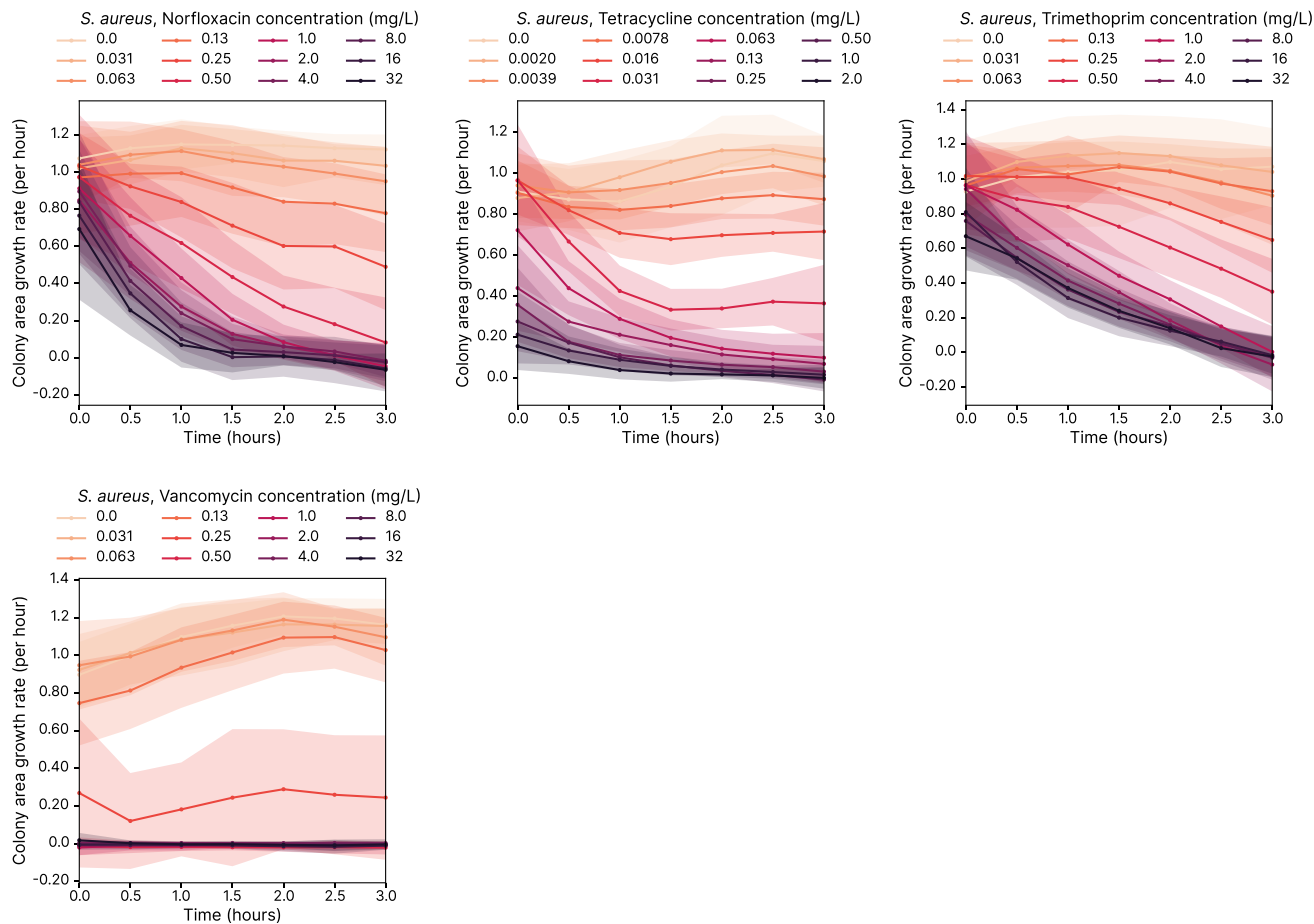

S1D Fig

Supplement: S1 Fig — Higher antibiotic concentrations lead to growth inhibition after varying time delays. A lighter hue corresponds with a lower antibiotic concentration. The lines show mean values with the shaded region indicating standard deviation between repeats. Each plot shows data from three or more repeats. The growth rates are binned to the nearest 30-minute interval. (PDF) [file ppat.1012924.s004.pdf]

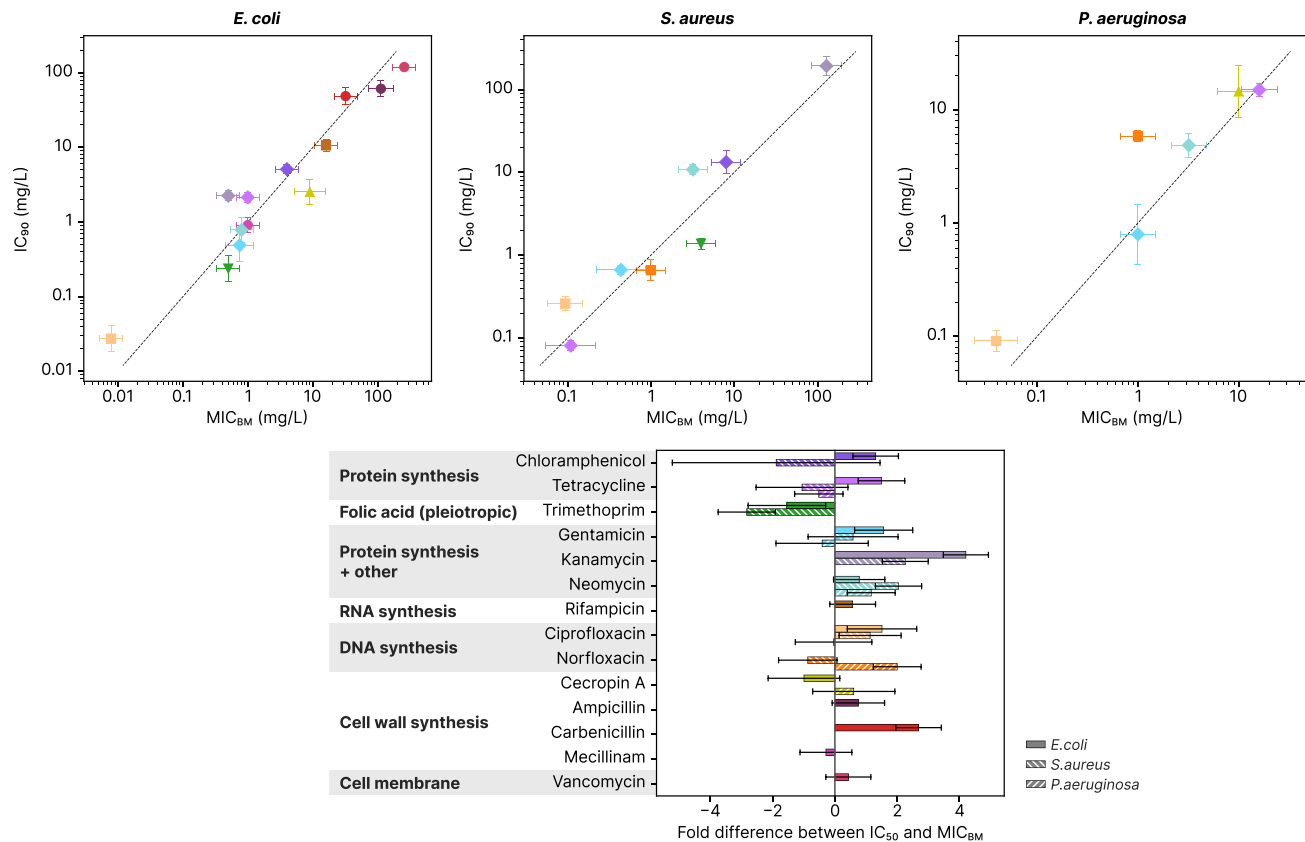

**S4 Fig**

Supplement: S4 Fig — The OD600 vs time curves from the broth microdilution experiment are shown in S22 Fig. The antibiotic type is indicated by hue. Each marker shows the mean and standard deviation between repeats, and the line x = y is shown. The fold difference plot shows how well MIC from growth rate (IC90) compares with MIC from broth microdilution (MICBM). The fold difference is computed as log2IC90MICBM, such that a value of for example 1 means IC90 is 2 times higher than MICBM, and -2 means 4 times lower. The bars show the mean and standard deviation between repeats. (PDF) [file ppat.1012924.s007.pdf]

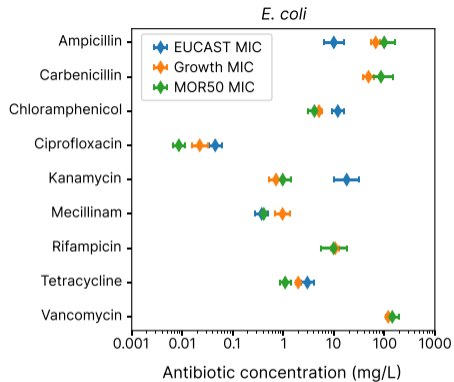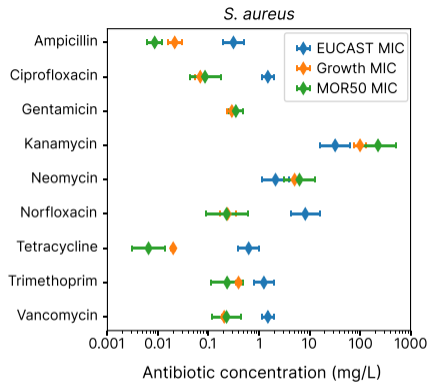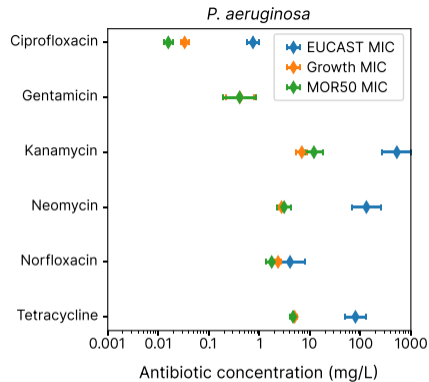

S5 Fig

Supplement: S5 Fig — (PDF) [file ppat.1012924.s008.pdf]

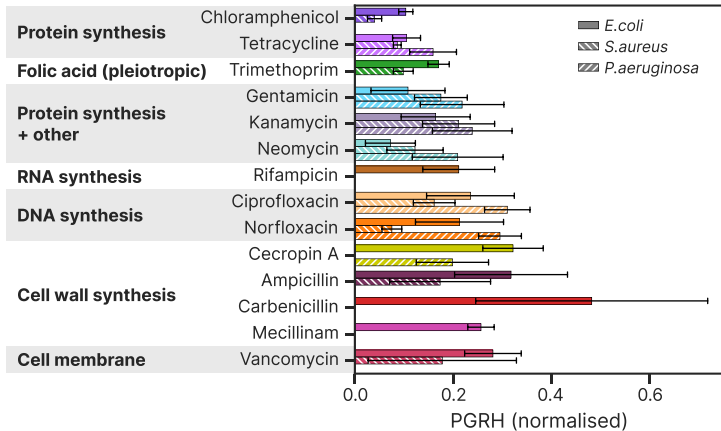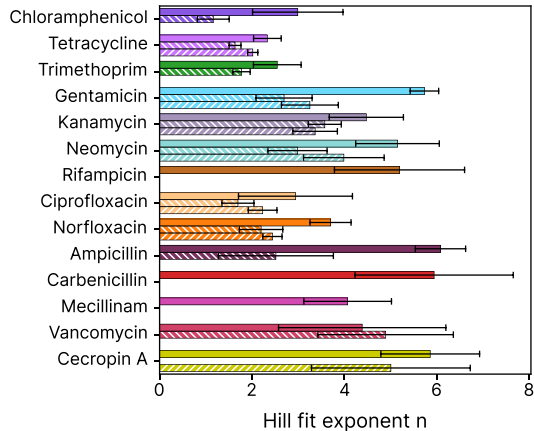

S7 Fig

Supplement: S7 Fig — PGRH has been normalised to the antibiotic-free growth rate for each species in the same time window. Each bar represents the mean and standard deviation between repeats, based on a least three repeats per condition. Each antibiotic has a dedicated hue, and each species has a separate shading. (PDF) [file ppat.1012924.s010.pdf]

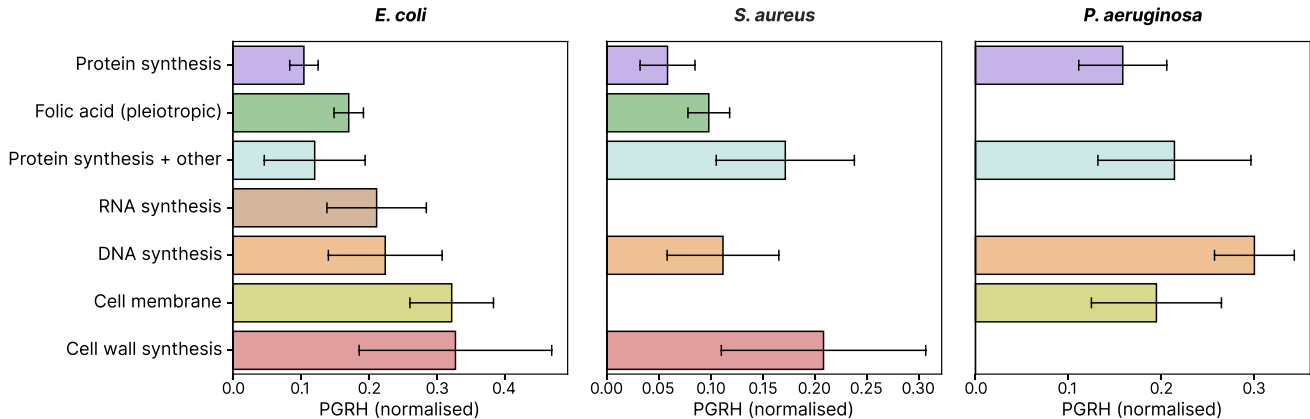

S8 Fig

Supplement: S8 Fig — The bars show the mean and standard deviation between repeats and antibiotics belonging to the relevant categories. PGRH has been normalised to the antibiotic-free growth rate for each species in the same time window. (PDF) [file ppat.1012924.s011.pdf]

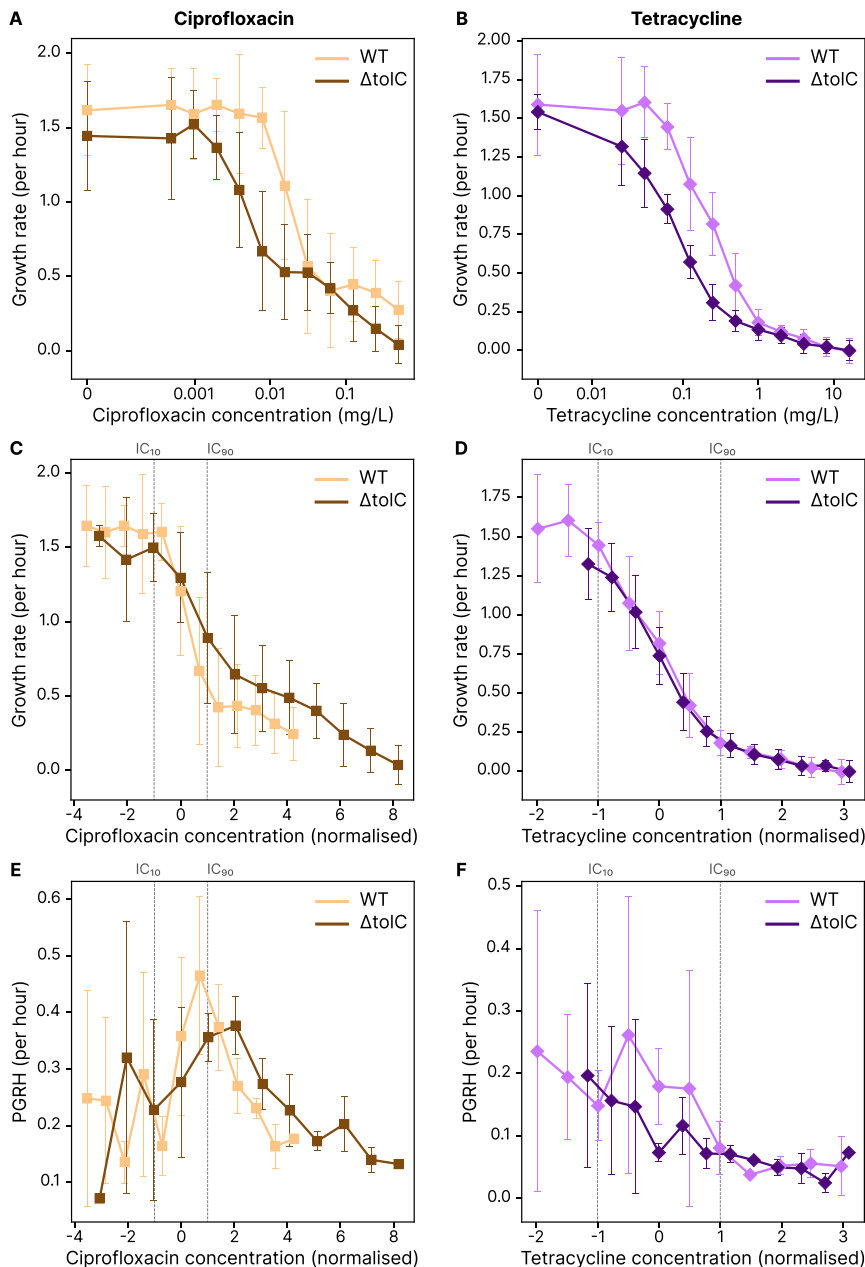

S10 Fig

Supplement: S10 Fig — The mutant has a lower MIC for both ciprofloxacin and tetracycline, but there is no significant difference in PGRH between the two. AB Growth rate vs antibiotic concentration plots for ciprofloxacin and tetracycline. The points show the mean and standard deviation between repeats. There are three repeats per condition. CD Normalizing the antibtioic concentration to IC50 shows how the susecptibility curve differes between the strains. A normalized concentration of 0 corresponds to the IC50, while 1 corresponds to IC90. The vertical lines show the IC10 and IC90 concentrations, respectively. EF shows population growth rate heterogeneity (PGRH) with antibiotic concentration normalised as in CD. (PDF) [file ppat.1012924.s013.pdf]

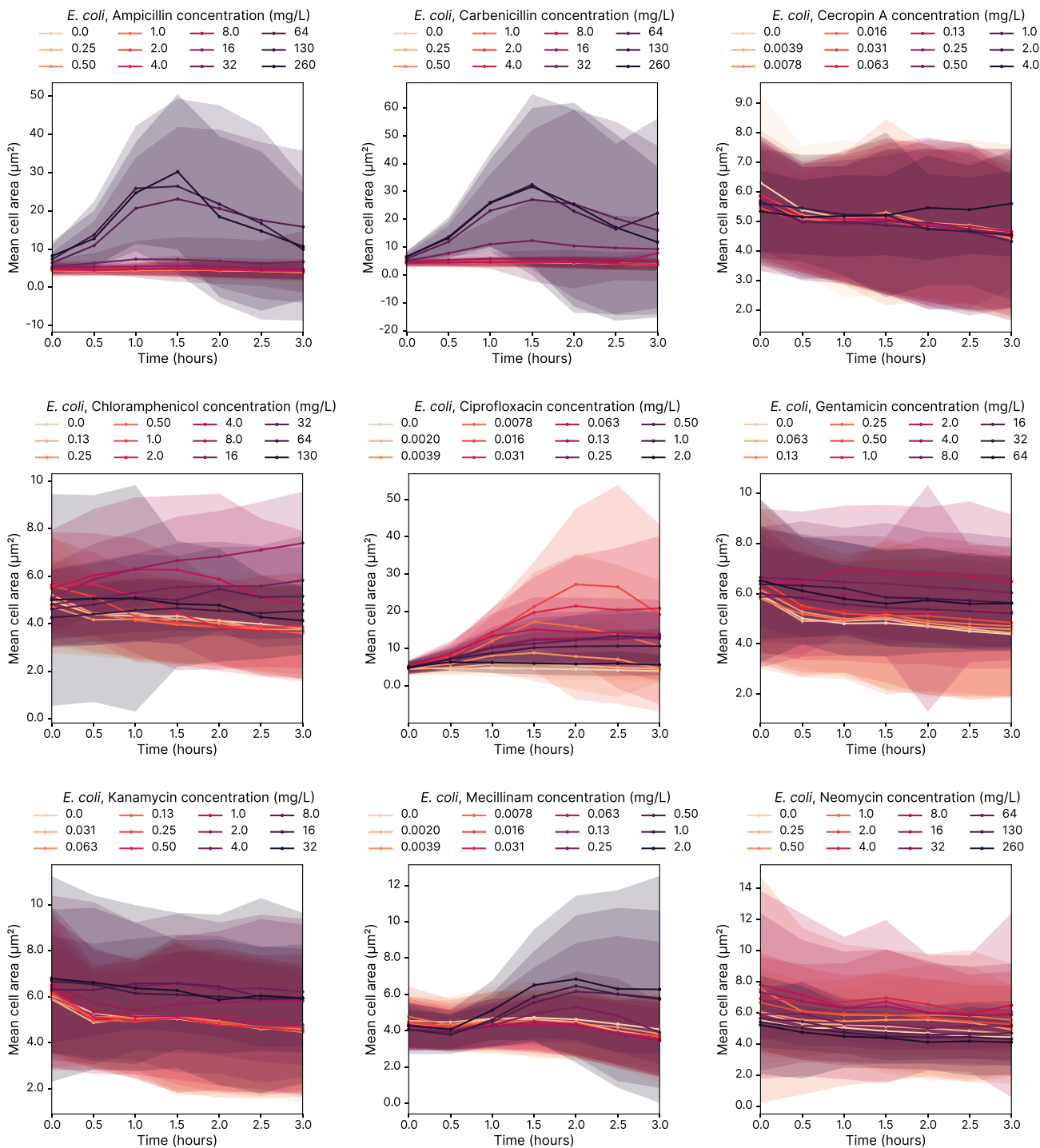

S11A Fig

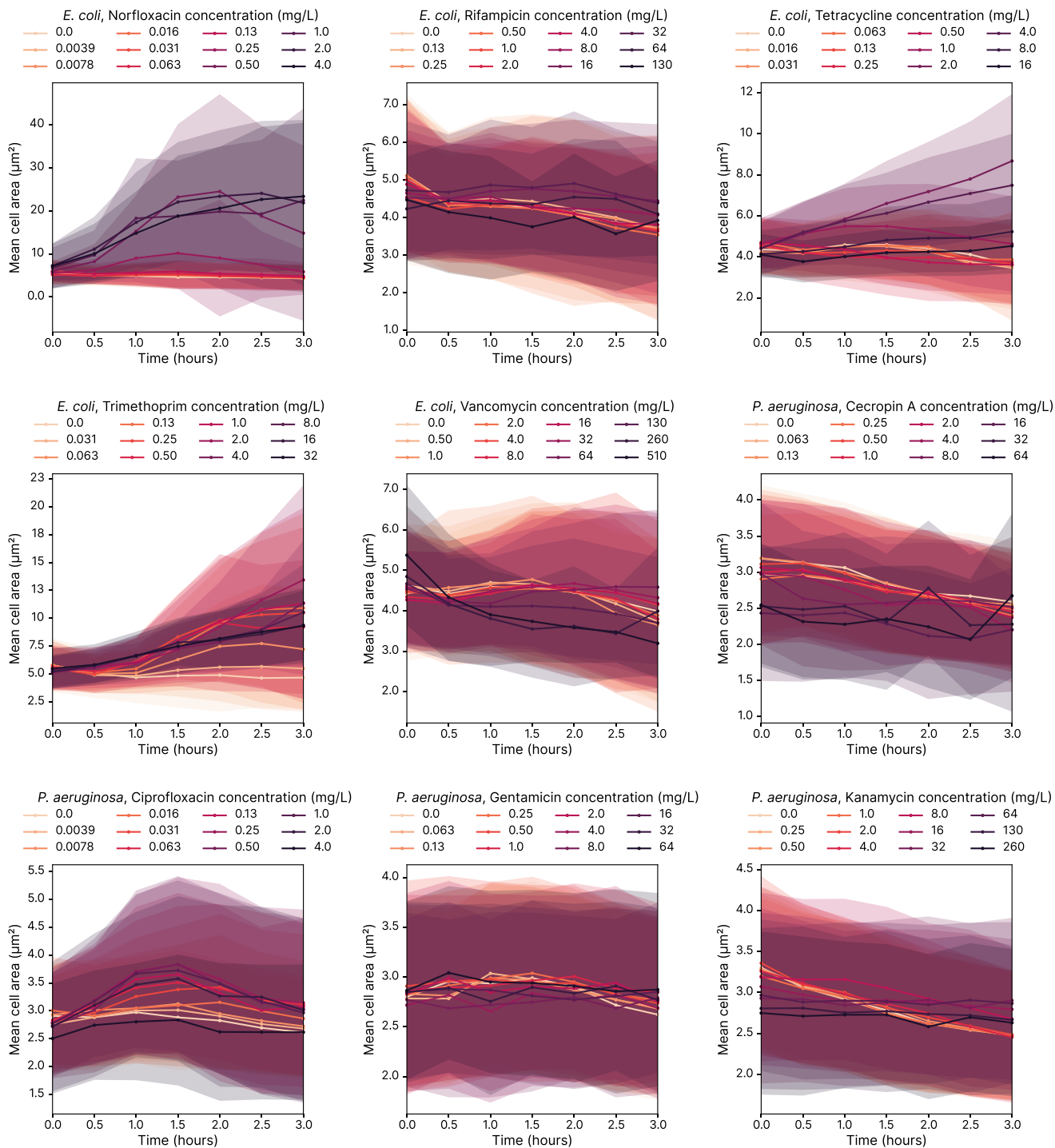

S11B Fig

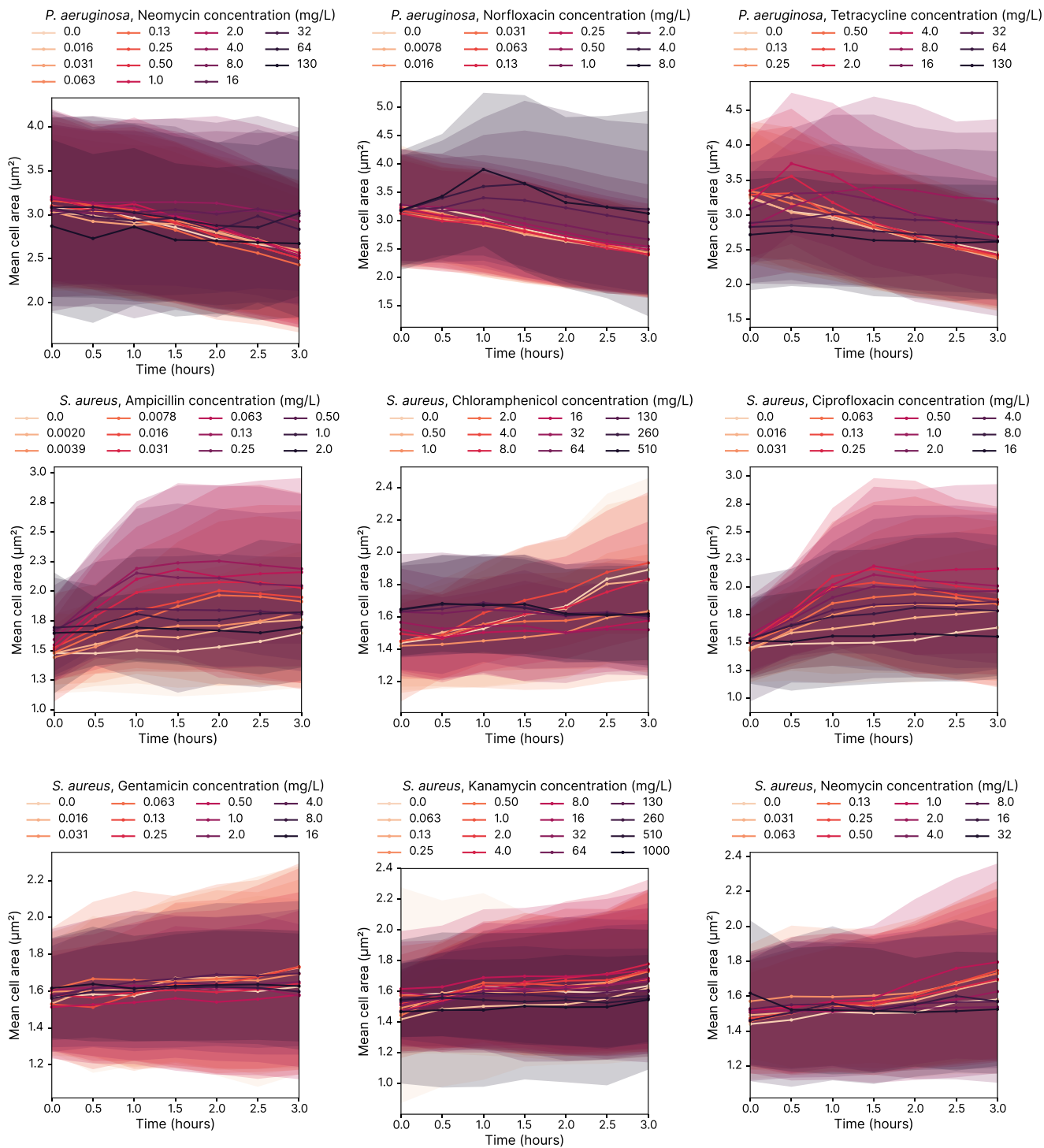

S11C Fig

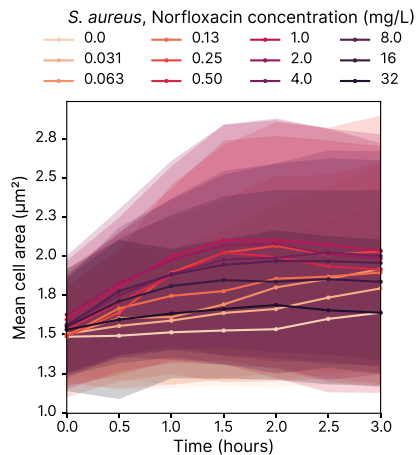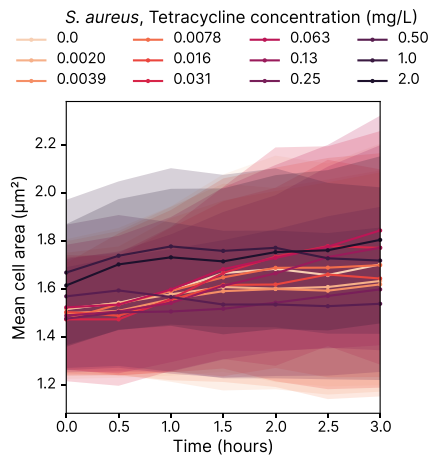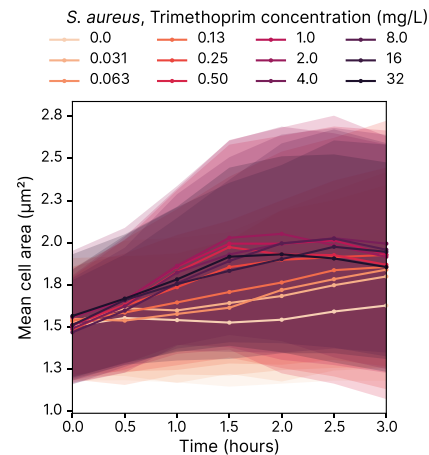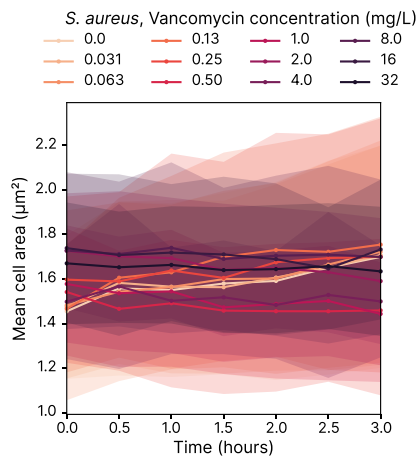

S11D Fig

Supplement: S11 Fig — Initially, all bacteria have the same morphology as they are introduced to the pads with antibiotics. As time progresses, many antibiotics cause a significant change in morphology for some concentrations. Generally, the largest differences are apparent after 2 hours of growth. Each line represents the mean area per cell for a given antibiotic concentration, and the shaded area represents the standard deviation with data originating from three or more repeats. Darker colours correspond to higher antibiotic concentration, and data points are binned to the nearest 30 minutes. The plots for E.coli with ciprofloxacin, mecillinam and chloramphenicol are also presented in Fig 4 and are included here for completeness. (PDF) [file ppat.1012924.s014.pdf]

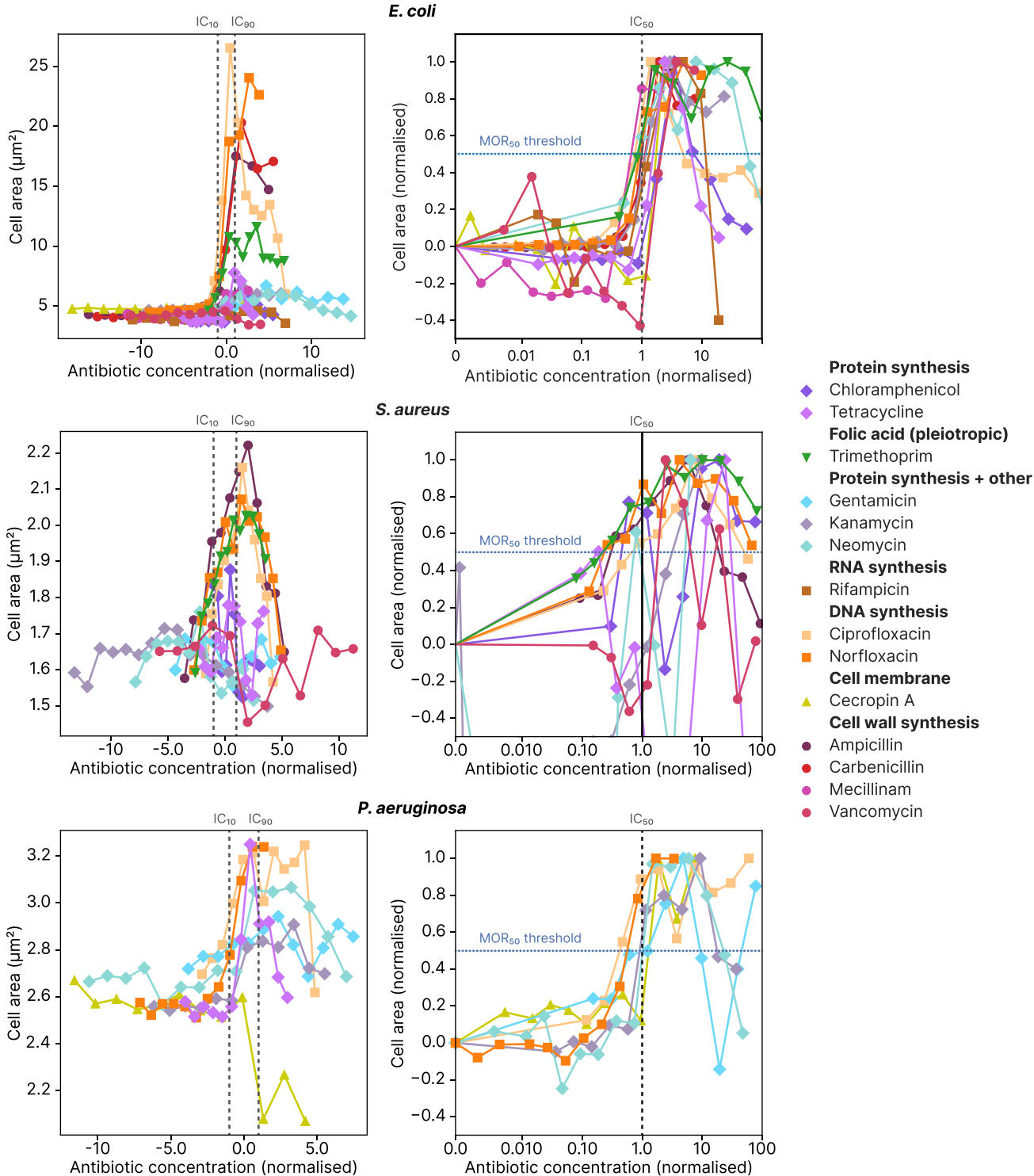

S15 Fig

Supplement: S15 Fig — The plot for E.coli is also shown in the main text but repeated here for completeness. For each species, curves are shown for all the tested antibiotics after 2.5 hours of incubation with the antibiotics. The antibiotic concentrations are normalised to growth MIC, and the cell area is normalised so that the no-antibiotic area is 0 and the maximum change in cell area is 1 (which for vancomycin entails inverting the area). The MOR50 threshold is shown as a horizontal line at 50% change, and the cell MIC is where the area curve first crosses this threshold. All the antibiotics produce a morphology change around the growth MIC. The degree of change varies strongly between antibiotics, which is here made apparent through the noise present in the area signal for antibiotics that induces a small change in morphology. The antibiotics are categorised by action mechanisms as inhibiting cell wall synthesis, protein synthesis, and nucleic acid synthesis, in addition to a negative control where no antibiotic was used. The plot for E.coli with normalized cell area is also presented in Fig 7 and is included here for completeness. (PDF) [file ppat.1012924.s018.pdf]

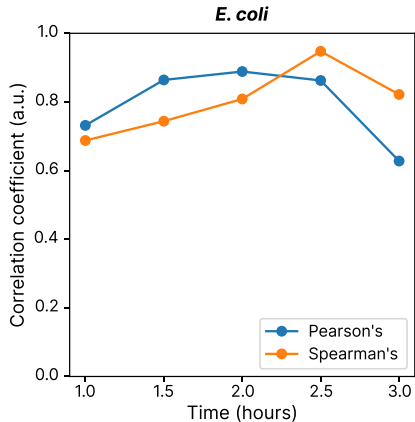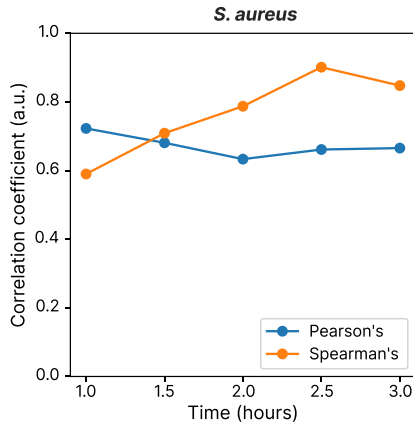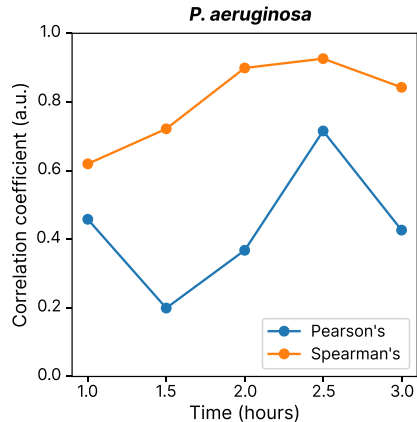

**S16 Fig**

Supplement: S16 Fig — All species show the best correlation at 2.5 hours of incubation on the MAP platform. (PDF) [file ppat.1012924.s019.pdf]

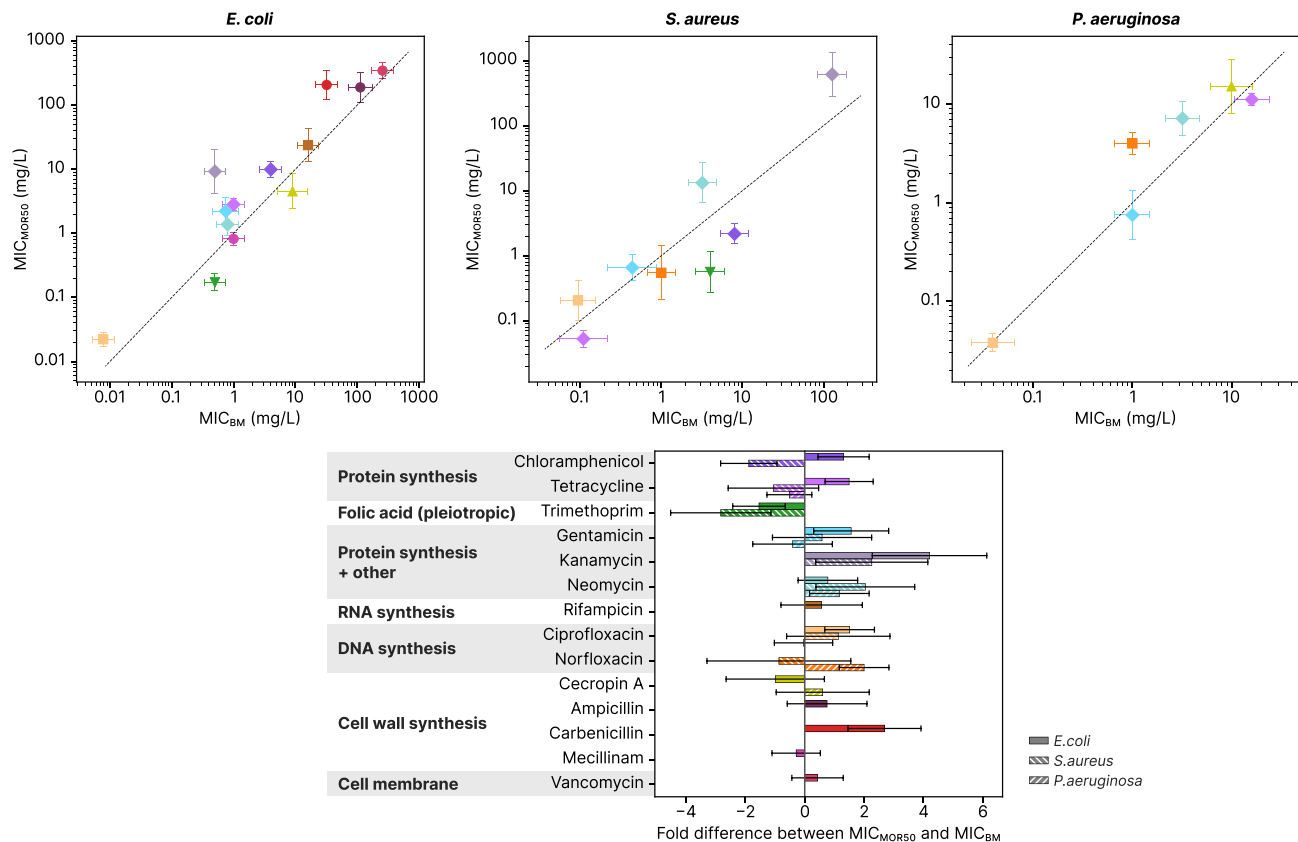

**S17 Fig**

Supplement: S17 Fig — Points represent the mean and standard deviation between repeats for each antibiotic/species combination. The fold difference plot shows how well MIC from MOR50 (MICMOR50) compares with MIC from broth microdilution (MICBM). The fold difference is computed as log2MICMOR50MICBM, such that a value of for example 1 means MICMOR50 is 2 times higher than MICBM, and -2 means 4 times lower. The bars show the mean and standard deviation between repeats. (PDF) [file ppat.1012924.s020.pdf]

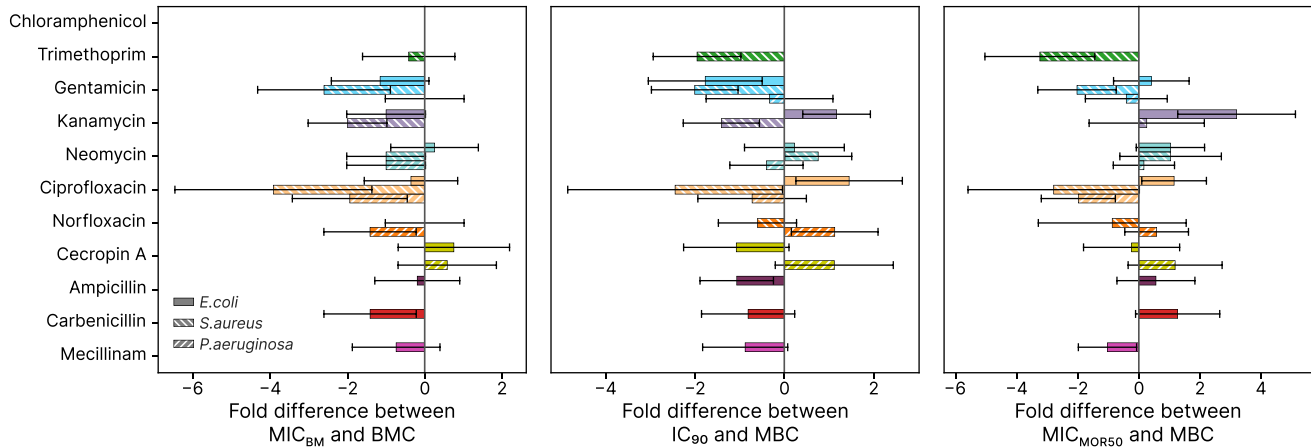

S18 Fig

Supplement: S18 Fig — The fold difference is computed as log2MICMBC, such that a value of for example 1 means MIC is 2 times higher than MBC, and -2 means 4 times lower. The bars show the mean and standard deviation between repeats. (PDF) [file ppat.1012924.s021.pdf]

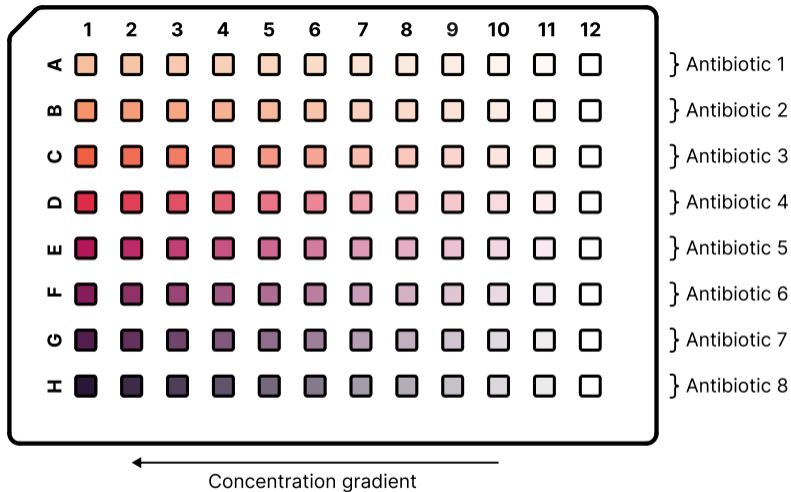

**S19 Fig**

Supplement: S19 Fig — (PDF) [file ppat.1012924.s022.pdf]

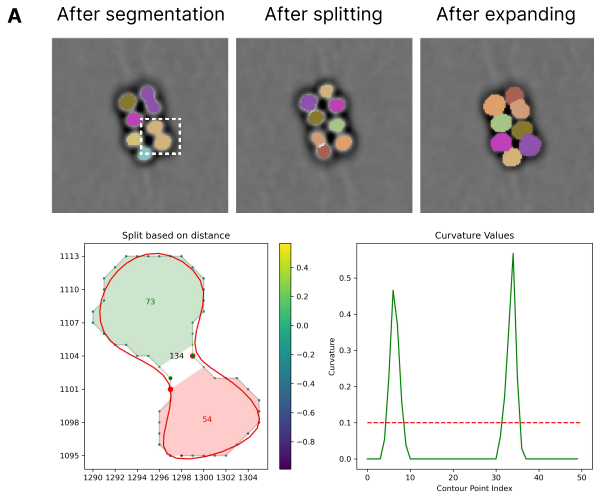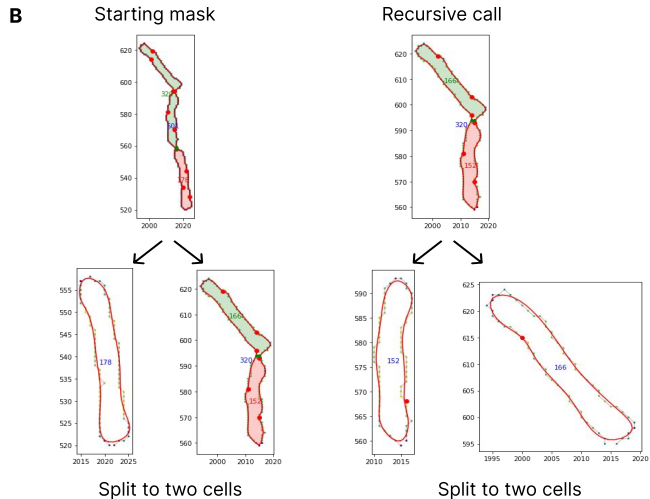

**S20 Fig**

Supplement: S20 Fig — A shows an example with S.aureus. A spline is fitted to the outline of the cells (shown in red), and its curvature is computed at each point. Positive curvature maps to where the surface curves outwards. Discarding all negative curvatures, the maximum curvature locations are found. If more than 10 points separate their position along the curve, and they are closer than the max width of the contour times the split factor, the split is performed. Using these points of maximum curvature as seed points, a local search is conducted around these points to find the pair of points that are closest to each other. B shows an example with E.coli where the recursive nature of the algorithm is highlighted. (PDF) [file ppat.1012924.s023.pdf]
