# Supplementary material for: Antibiotics change the population growth rate heterogeneity and morphology of bacteria
Source: PLoS Pathog. 2025 Feb 5;21(2):e1012924. doi: 10.1371/journal.ppat.1012924 (PMC11835381; doi:10.1371/journal.ppat.1012924)
Supplement: S2 Fig — The markers show mean and standard deviation between repeats. A Hill fit is performed for each antibiotic, with the vertical lines showing IC10 and IC90 concentrations, where the growth rate is inhibited by 10% and 90%, respectively. The plots for E.coli with tetracycline, rifampicin, and ampicillin are also presented in Fig 2 and are included here for completeness. (PDF) [file ppat.1012924.s005.pdf]

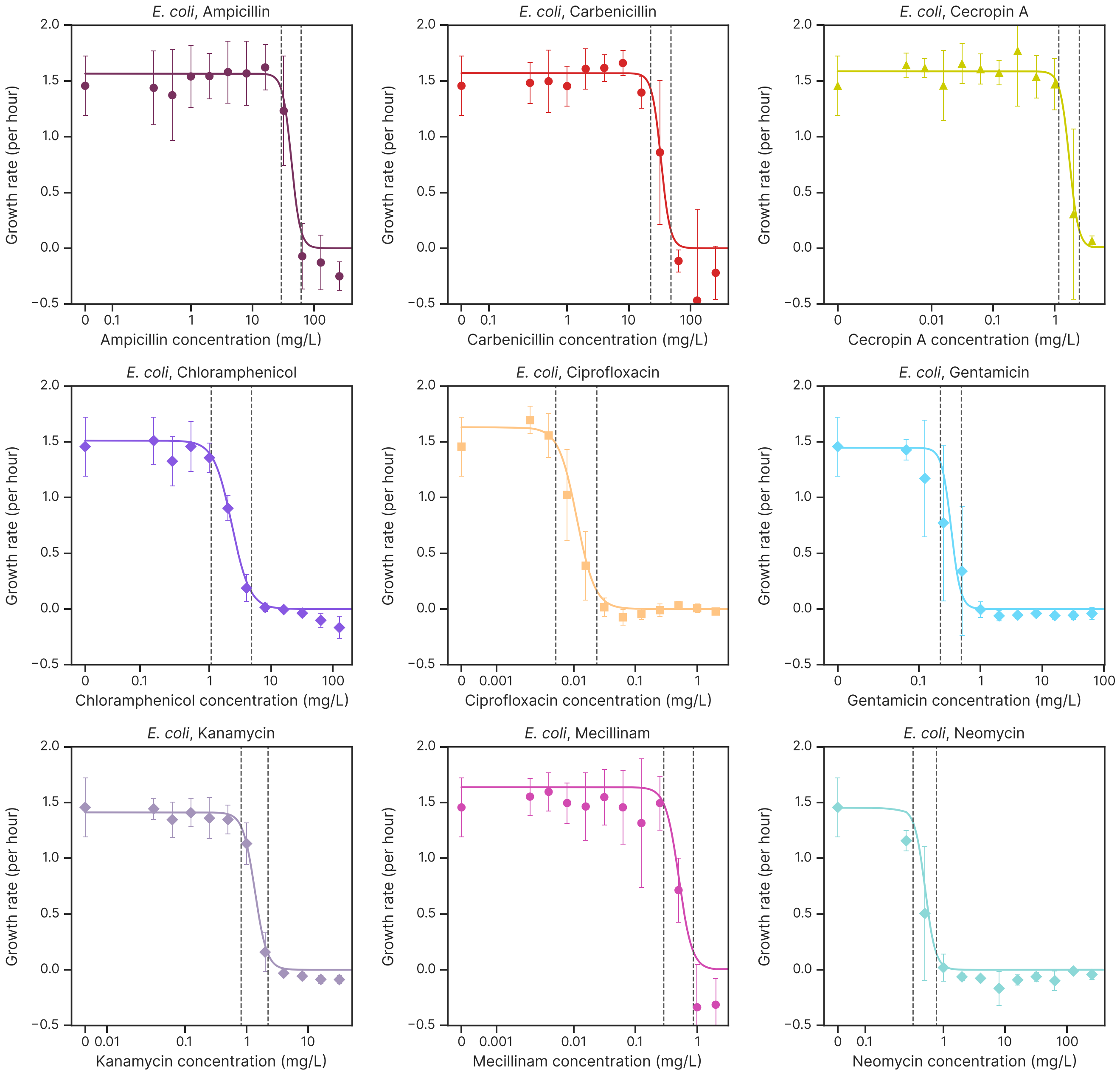

E. coli, Ampicillin
Ampicillin concentration (mg/L)
E. coli, Carbenicillin
Carbenicillin concentration (mg/L)
E. coli, Cecropin A
Cecropin A concentration (mg/L)
E. coli, Chloramphenicol
Chloramphenicol concentration (mg/L)
E. coli, Ciprofloxacin
Ciprofloxacin concentration (mg/L)
E. coli, Gentamicin
Gentamicin concentration (mg/L)
E. coli, Kanamycin
Kanamycin concentration (mg/L)
E. coli, Mecillinam
Mecillinam concentration (mg/L)
E. coli, Neomycin
Neomycin concentration (mg/L)
Growth rate (per hour)

S2A Fig

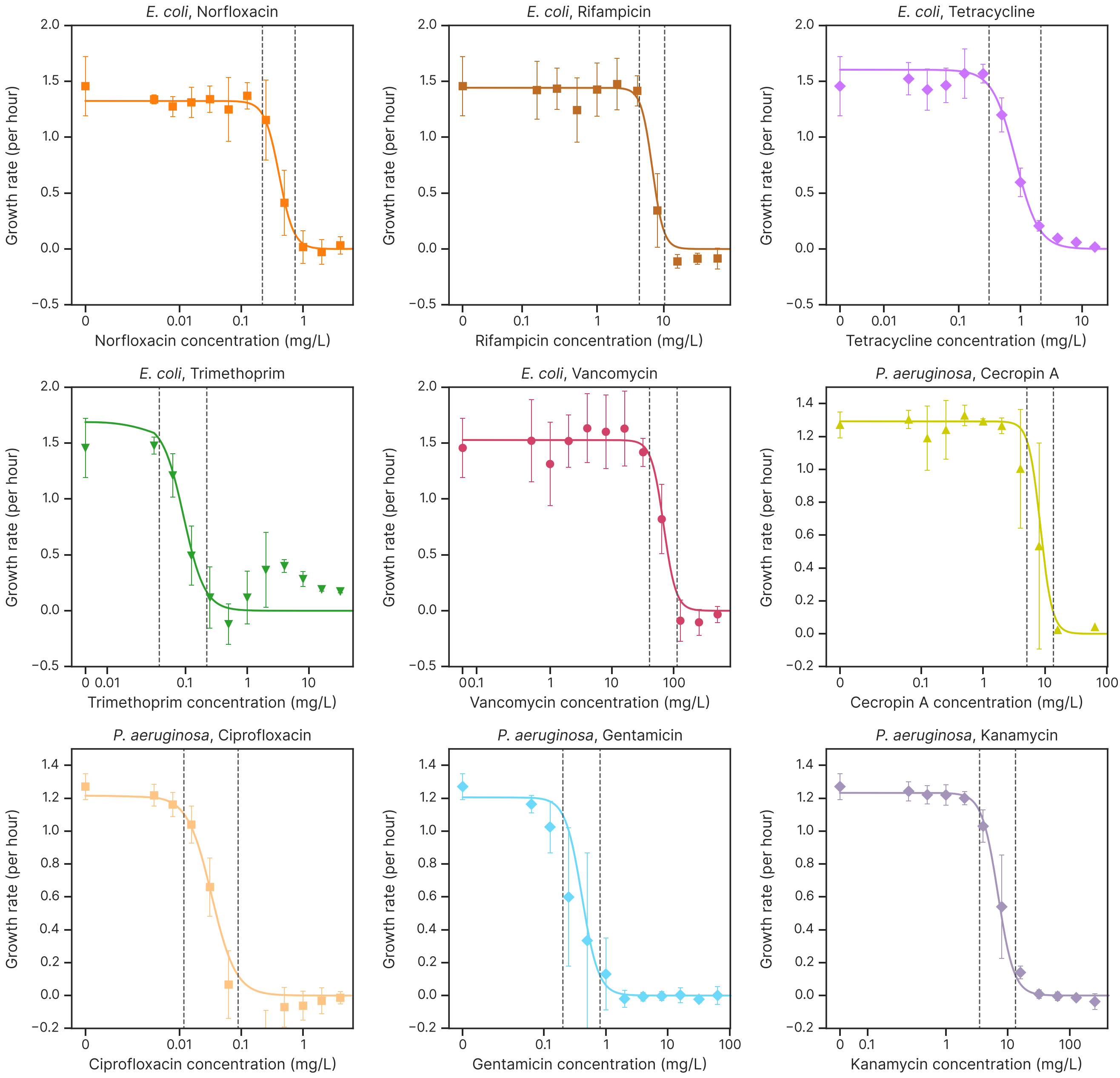

E. coli, Norfloxacin
E. coli, Rifampicin
E. coli, Tetracycline
E. coli, Trimethoprim
E. coli, Vancomycin
P. aeruginosa, Cecropin A
P. aeruginosa, Ciprofloxacin
P. aeruginosa, Gentamicin
P. aeruginosa, Kanamycin
Growth rate (per hour)
Norfloxacin concentration (mg/L)
Rifampicin concentration (mg/L)
Tetracycline concentration (mg/L)
Trimethoprim concentration (mg/L)
Vancomycin concentration (mg/L)
Cecropin A concentration (mg/L)
Ciprofloxacin concentration (mg/L)
Gentamicin concentration (mg/L)
Kanamycin concentration (mg/L)

S2B Fig

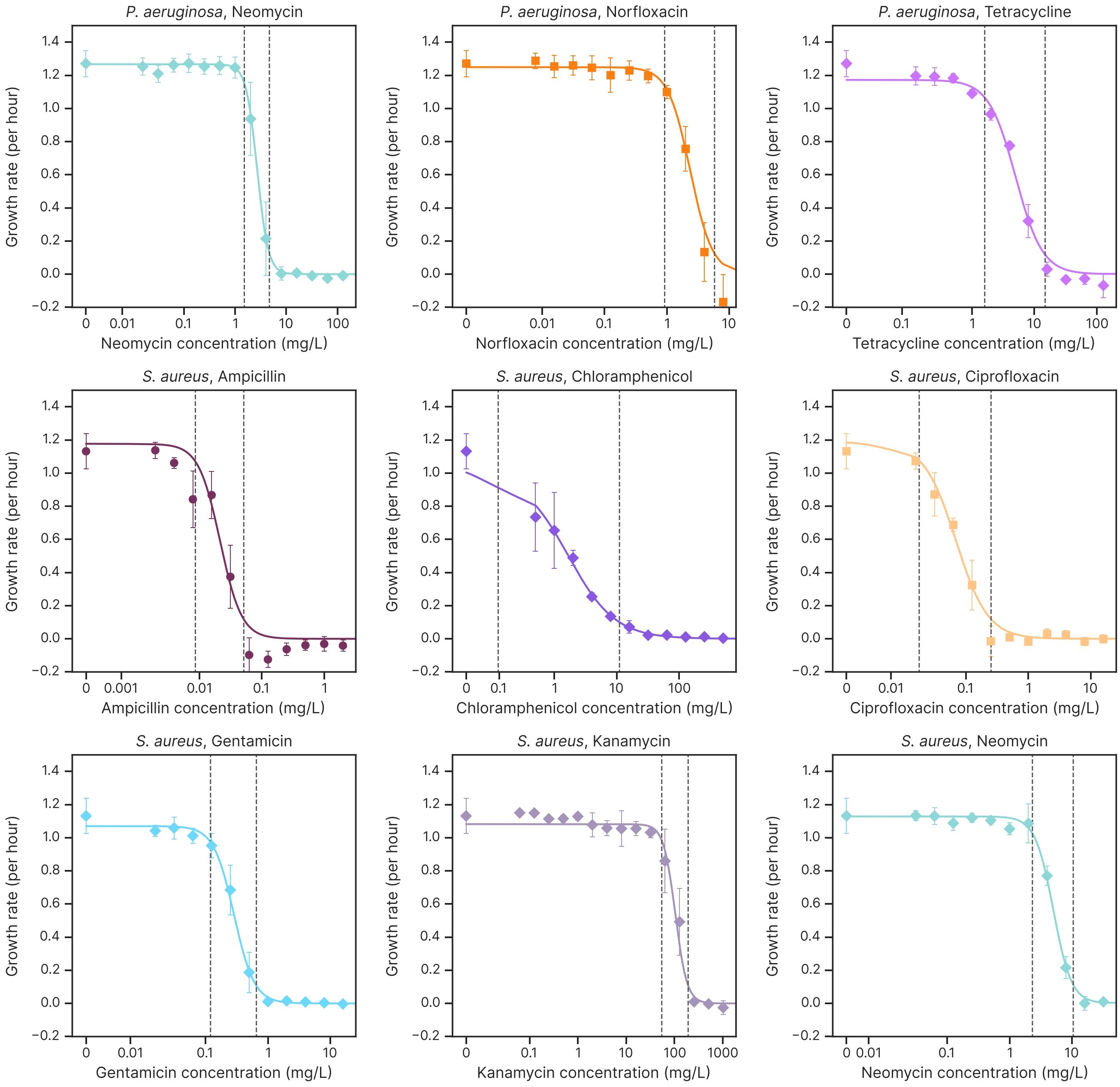

P. aeruginosa, Neomycin
P. aeruginosa, Norfloxacin
P. aeruginosa, Tetracycline
S. aureus, Ampicillin
S. aureus, Chloramphenicol
S. aureus, Ciprofloxacin
S. aureus, Gentamicin
S. aureus, Kanamycin
S. aureus, Neomycin
Growth rate (per hour)
Neomycin concentration (mg/L)
Norfloxacin concentration (mg/L)
Tetracycline concentration (mg/L)
Ampicillin concentration (mg/L)
Chloramphenicol concentration (mg/L)
Ciprofloxacin concentration (mg/L)
Gentamicin concentration (mg/L)
Kanamycin concentration (mg/L)

S2C Fig

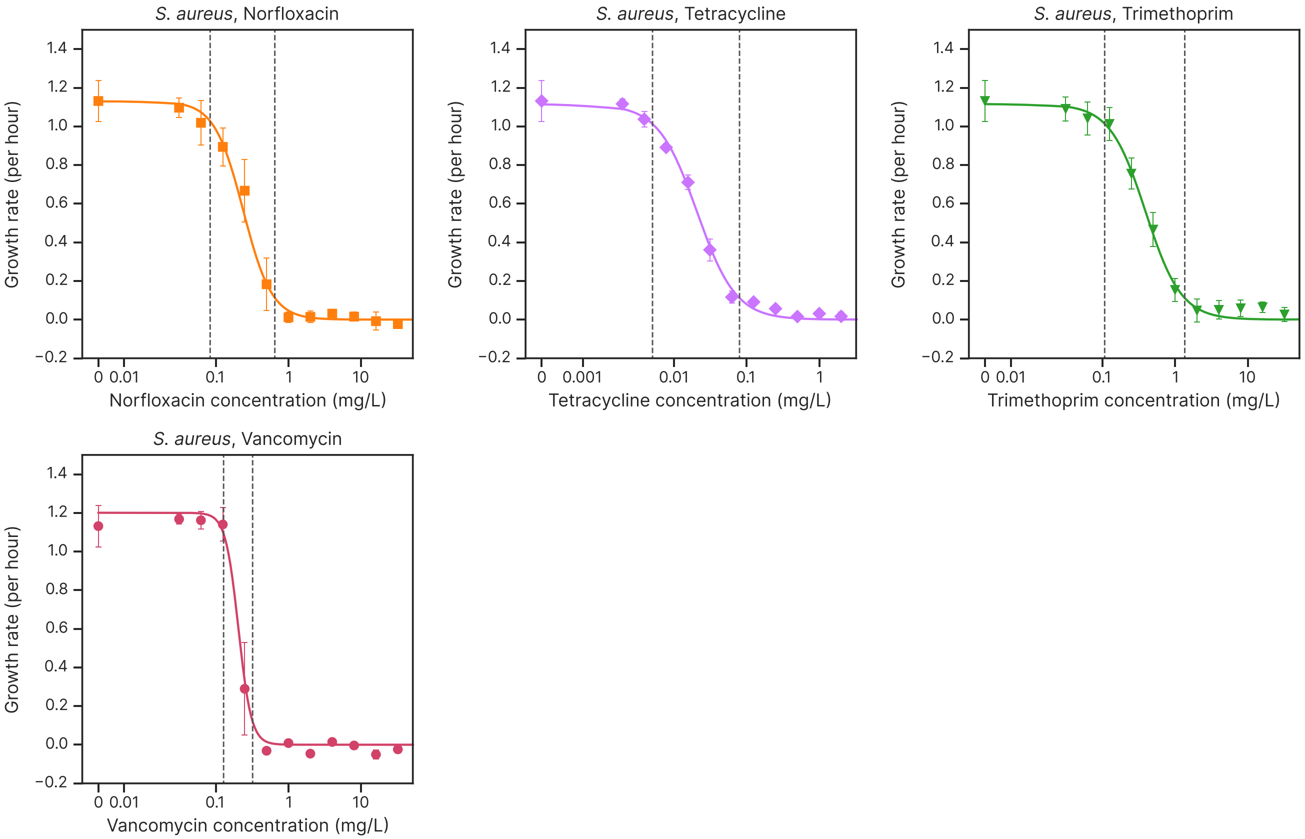

S. aureus, Norfloxacin
Growth rate (per hour)
Norfloxacin concentration (mg/L)
S. aureus, Tetracycline
Tetracycline concentration (mg/L)
S. aureus, Trimethoprim
Trimethoprim concentration (mg/L)
S. aureus, Vancomycin
Vancomycin concentration (mg/L)

S2D Fig
