# Supplementary material for: Antibiotics change the population growth rate heterogeneity and morphology of bacteria
Source: PLoS Pathog. 2025 Feb 5;21(2):e1012924. doi: 10.1371/journal.ppat.1012924 (PMC11835381; doi:10.1371/journal.ppat.1012924)
Supplement: S3 Fig — Each repeat is plotted individually, and Hill fits performed for each repeat. Data used is for the time between 2 and 3 hours of growth. The vertical lines show the IC90 concentrations where growth is inhibited by 90%, which we define as MIC. (PDF) [file ppat.1012924.s006.pdf]

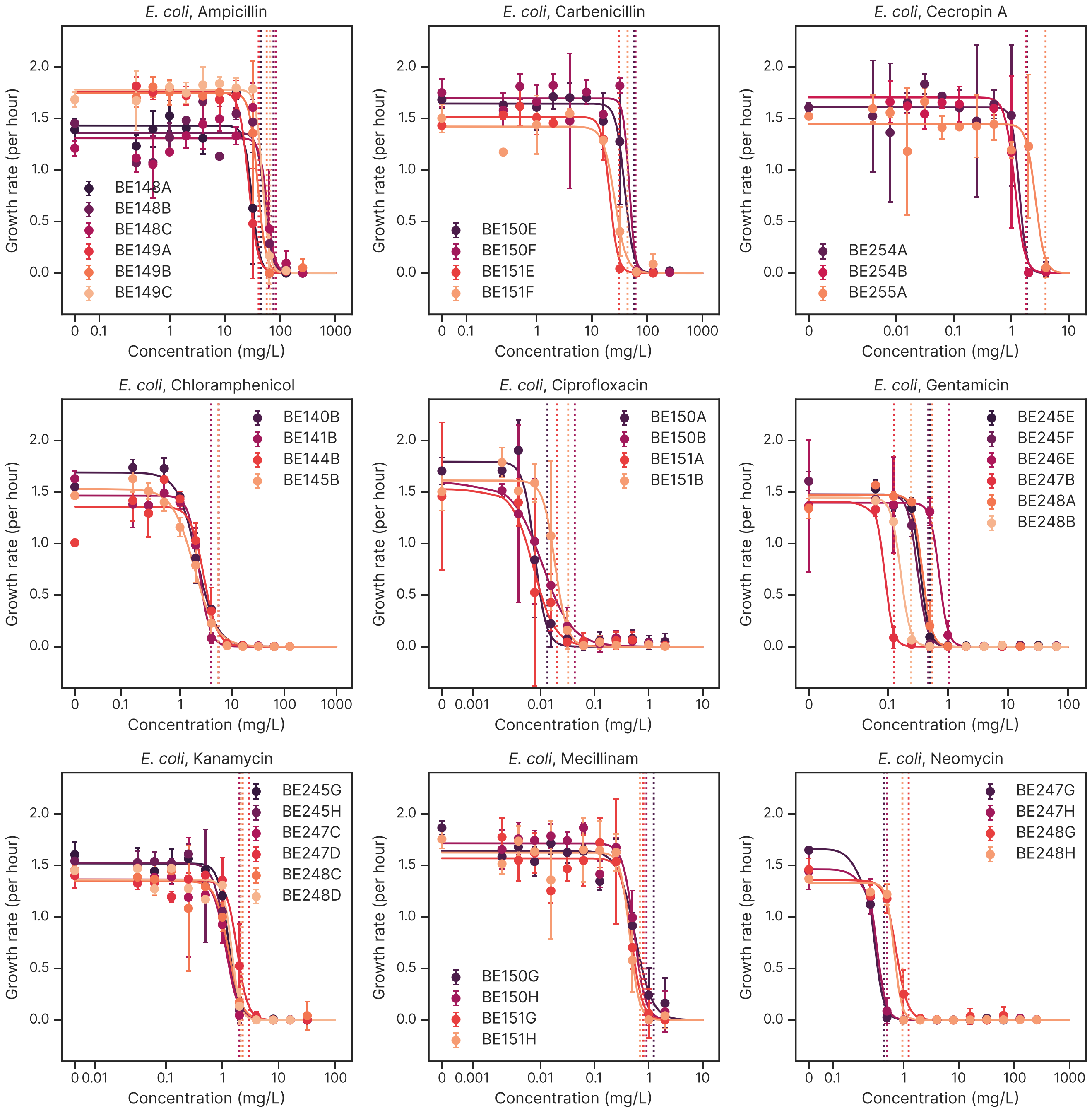
E. coli, Ampicillin
BE148A
BE148B
BE148C
BE149A
BE149B
BE149C
E. coli, Carbenicillin
BE150E
BE150F
BE151E
BE151F
E. coli, Cecropin A
BE254A
BE254B
BE255A
E. coli, Chloramphenicol
BE140B
BE141B
BE144B
BE145B
E. coli, Ciprofloxacin
BE150A
BE150B
BE151A
BE151B
E. coli, Gentamicin
BE245E
BE245F
BE246E
BE247B
BE248A
BE248B
E. coli, Kanamycin
BE245G
BE245H
BE247C
BE247D
BE248C
BE248D
E. coli, Mecillinam
BE150G
BE150H
BE151G
BE151H
E. coli, Neomycin
BE247G
BE247H
BE248G
BE248H
Growth rate (per hour)
Concentration (mg/L)

S3A Fig

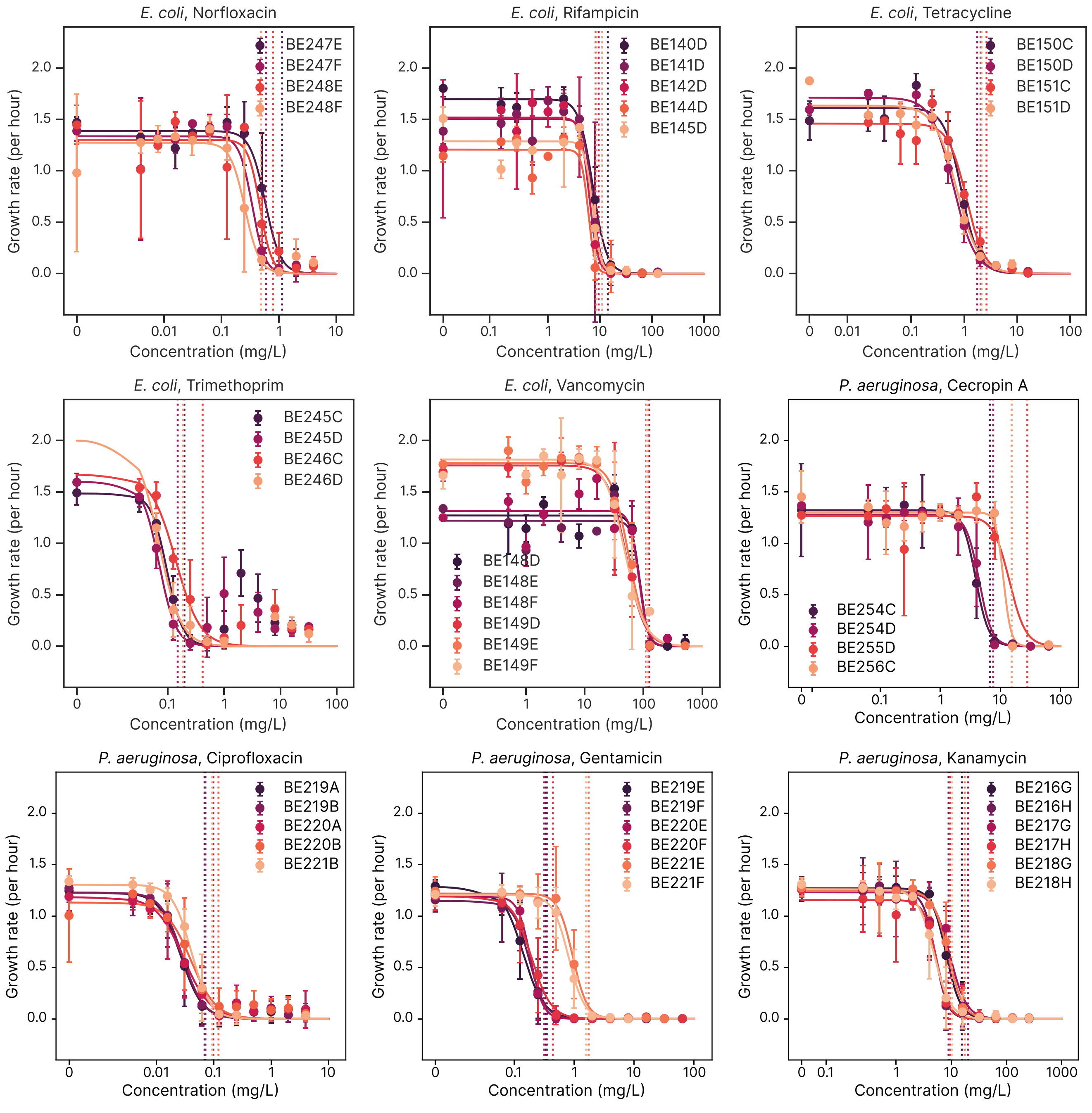
E. coli, Norfloxacin
E. coli, Rifampicin
E. coli, Tetracycline
E. coli, Trimethoprim
E. coli, Vancomycin
P. aeruginosa, Cecropin A
P. aeruginosa, Ciprofloxacin
P. aeruginosa, Gentamicin
P. aeruginosa, Kanamycin
Growth rate (per hour)
Concentration (mg/L)
BE247E
BE247F
BE248E
BE248F
BE140D
BE141D
BE142D
BE144D
BE145D
BE150C
BE150D
BE151C
BE151D
BE245C
BE245D
BE246C
BE246D
BE148D
BE148E
BE148F
BE149D
BE149E
BE149F
BE254C
BE254D
BE255D
BE256C
BE219A
BE219B
BE220A
BE220B
BE221B
BE219E
BE219F
BE220E
BE220F
BE221E
BE221F
BE216G
BE216H
BE217G
BE217H
BE218G
BE218H

S3B Fig

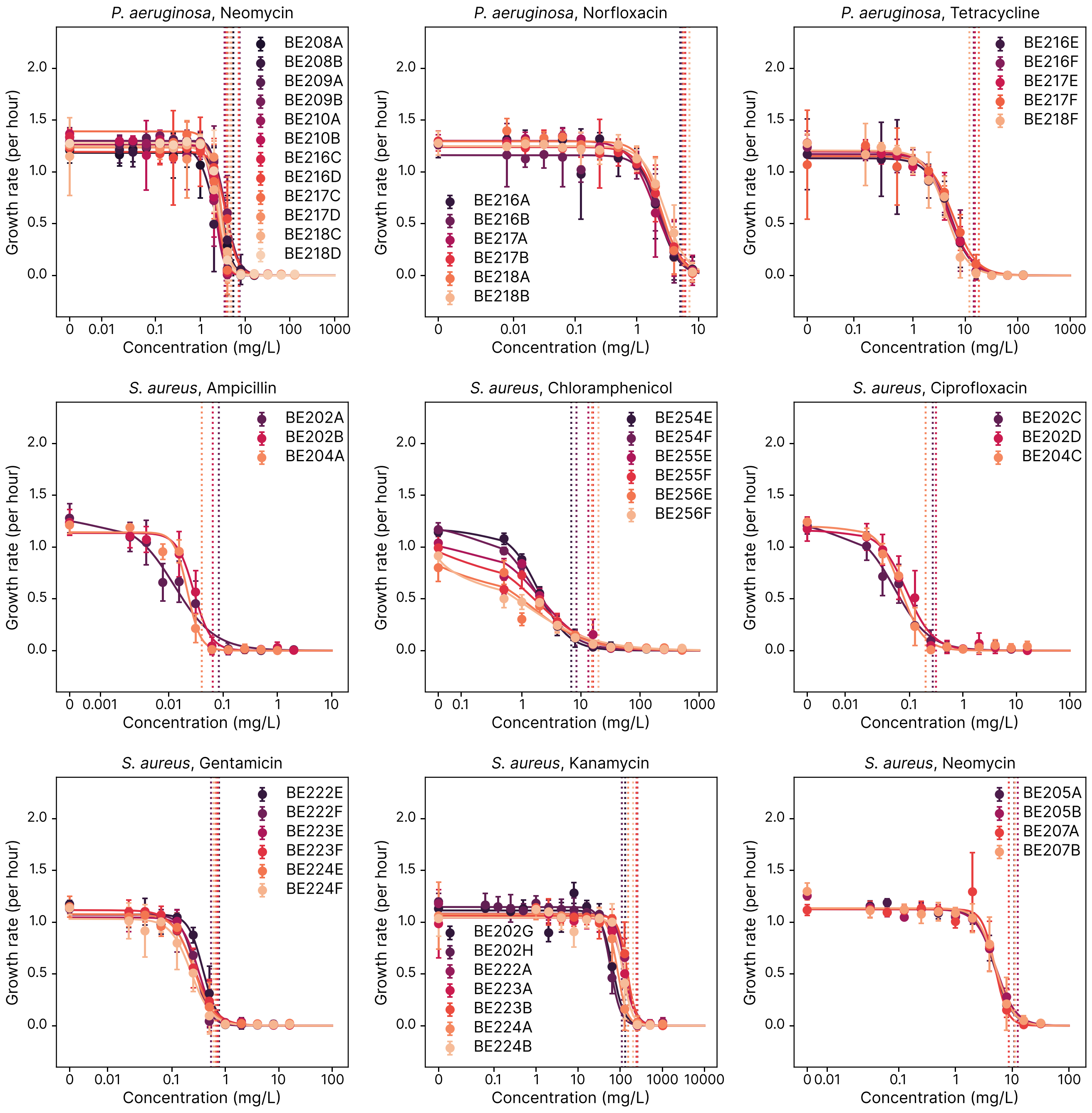
P. aeruginosa, Neomycin
BE208A
BE208B
BE209A
BE209B
BE210A
BE210B
BE216C
BE216D
BE217C
BE217D
BE218C
BE218D
P. aeruginosa, Norfloxacin
BE216A
BE216B
BE217A
BE217B
BE218A
BE218B
P. aeruginosa, Tetracycline
BE216E
BE216F
BE217E
BE217F
BE218F
S. aureus, Ampicillin
BE202A
BE202B
BE204A
S. aureus, Chloramphenicol
BE254E
BE254F
BE255E
BE255F
BE256E
BE256F
S. aureus, Ciprofloxacin
BE202C
BE202D
BE204C
S. aureus, Gentamicin
BE222E
BE222F
BE223E
BE223F
BE224E
BE224F
S. aureus, Kanamycin
BE202G
BE202H
BE222A
BE223A
BE223B
BE224A
BE224B
S. aureus, Neomycin
BE205A
BE205B
BE207A
BE207B
Growth rate (per hour)
Concentration (mg/L)

S3C Fig

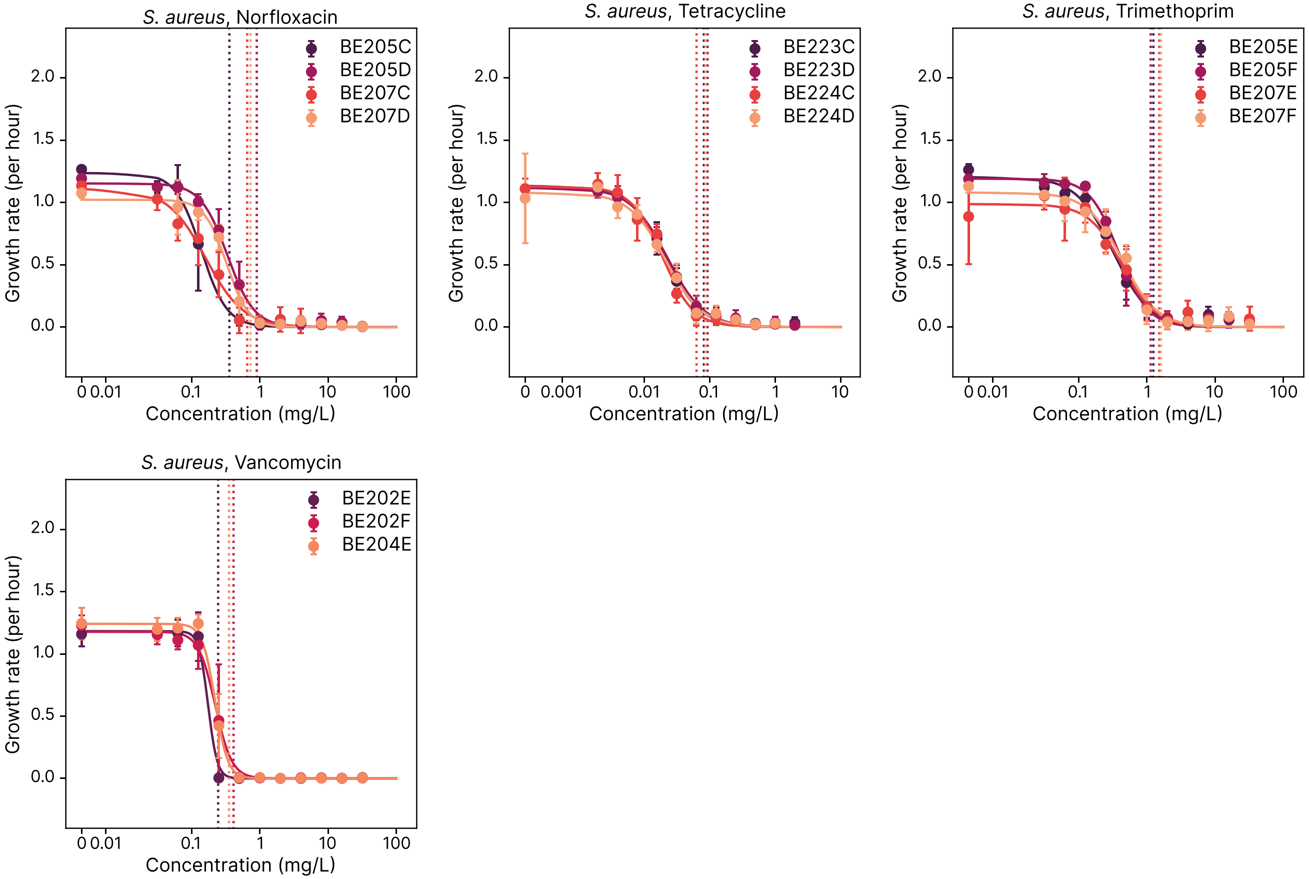
S. aureus, Norfloxacin
BE205C
BE205D
BE207C
BE207D
Growth rate (per hour)
Concentration (mg/L)
S. aureus, Tetracycline
BE223C
BE223D
BE224C
BE224D
S. aureus, Trimethoprim
BE205E
BE205F
BE207E
BE207F
S. aureus, Vancomycin
BE202E
BE202F
BE204E

S3D Fig
