# Supplementary material for: Antibiotics change the population growth rate heterogeneity and morphology of bacteria
Source: PLoS Pathog. 2025 Feb 5;21(2):e1012924. doi: 10.1371/journal.ppat.1012924 (PMC11835381; doi:10.1371/journal.ppat.1012924)
Supplement: S6 Fig — PGRH shows the variation in growth rate between colonies in the same growth environment, which we define as the standard deviation of colony growth rate for one pad at one point in time. This value is averaged between 1 to 2.5 hours of incubation on the MAP. The markers show mean and standard deviation between repeats. There are at least three repeats per antibiotic/species combination. The white marker in each plot shows the concentrations selected for subsequent PGRH analysis. The vertical lines correspond to the IC10 and IC90 concentrations where the growth rate is inhibited by 10% and 90%, respectively. We see that for some antibiotics, high concentrations produce a much higher PGRH and that this change is linked to the MIC. For others, like chloramphenicol, there is a drop in PGRH linked to the use of the antibiotic, also around the MIC. The plots for E.coli with tetracycline, rifampicin, and ampicillin are also presented in Fig 2 and are included here for completeness. (PDF) [file ppat.1012924.s009.pdf]

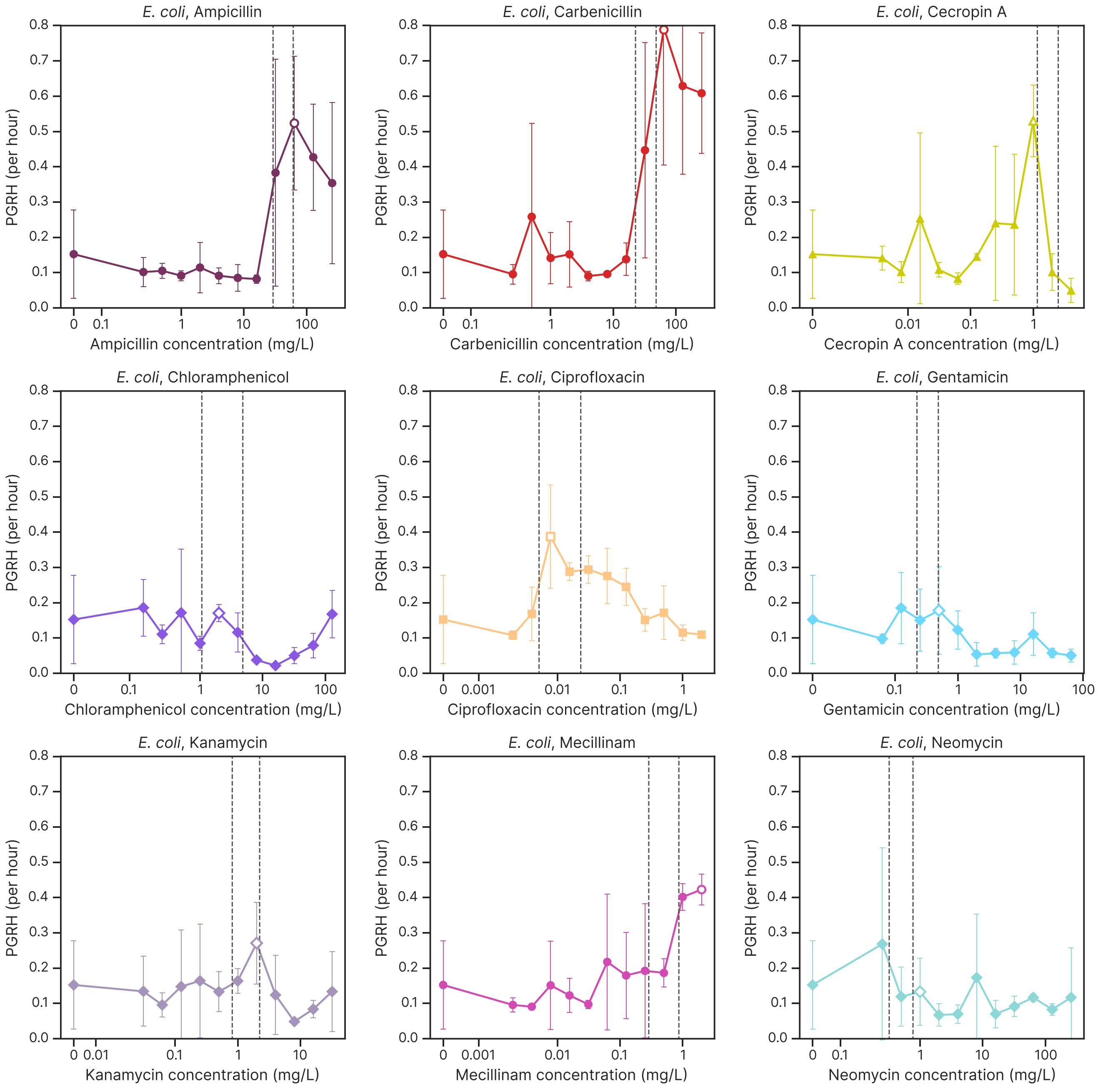

E. coli, Ampicillin
PGRH (per hour)
Ampicillin concentration (mg/L)
E. coli, Carbenicillin
Carbenicillin concentration (mg/L)
E. coli, Cecropin A
Cecropin A concentration (mg/L)
E. coli, Chloramphenicol
Chloramphenicol concentration (mg/L)
E. coli, Ciprofloxacin
Ciprofloxacin concentration (mg/L)
E. coli, Gentamicin
Gentamicin concentration (mg/L)
E. coli, Kanamycin
Kanamycin concentration (mg/L)
E. coli, Mecillinam
Mecillinam concentration (mg/L)
E. coli, Neomycin
Neomycin concentration (mg/L)

S6A Fig

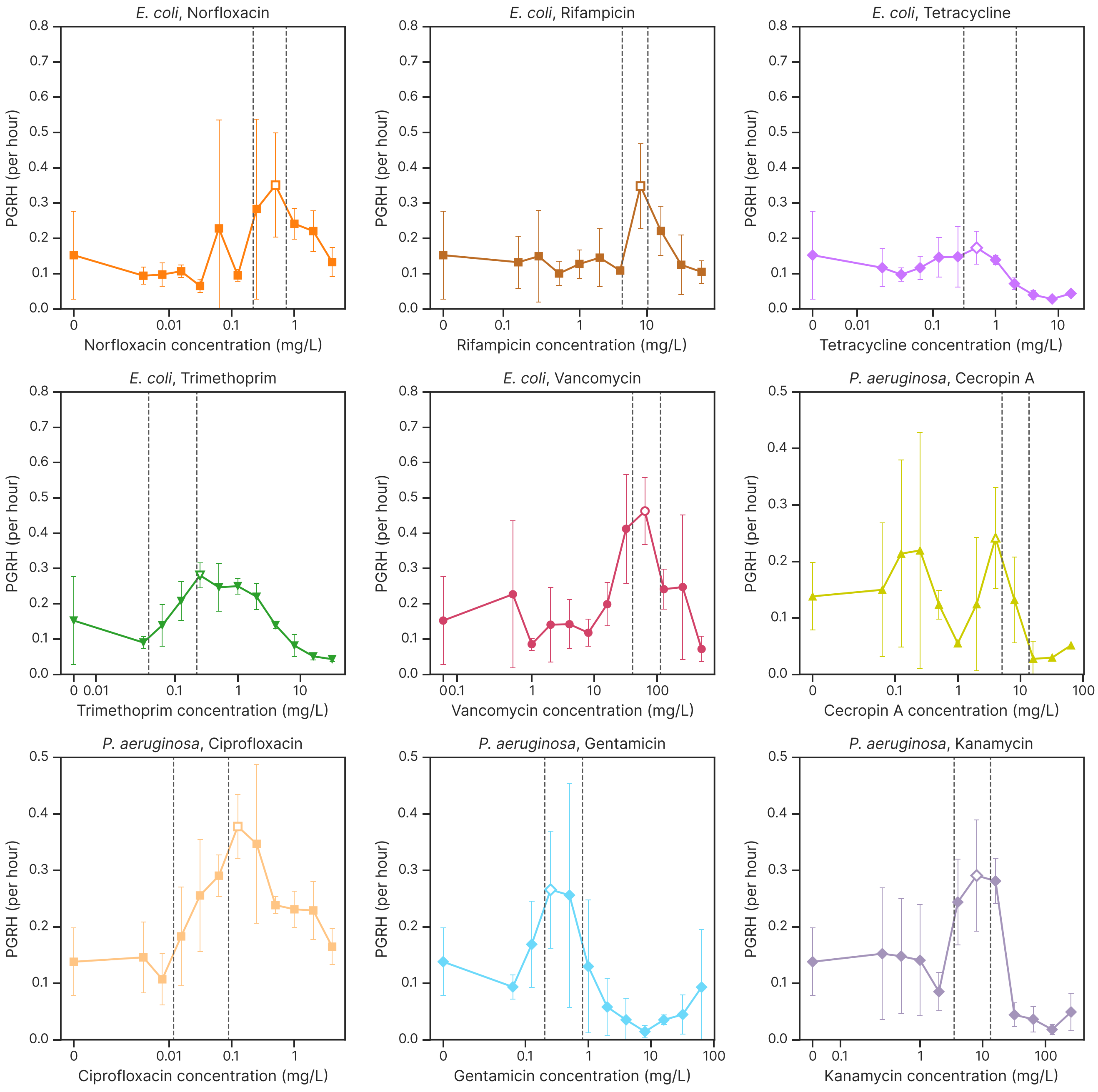

E. coli, Norfloxacin
E. coli, Rifampicin
E. coli, Tetracycline
E. coli, Trimethoprim
E. coli, Vancomycin
P. aeruginosa, Cecropin A
P. aeruginosa, Ciprofloxacin
P. aeruginosa, Gentamicin
P. aeruginosa, Kanamycin
PGRH (per hour)
Norfloxacin concentration (mg/L)
Rifampicin concentration (mg/L)
Tetracycline concentration (mg/L)
Trimethoprim concentration (mg/L)
Vancomycin concentration (mg/L)
Cecropin A concentration (mg/L)
Ciprofloxacin concentration (mg/L)
Gentamicin concentration (mg/L)
Kanamycin concentration (mg/L)

S6B Fig

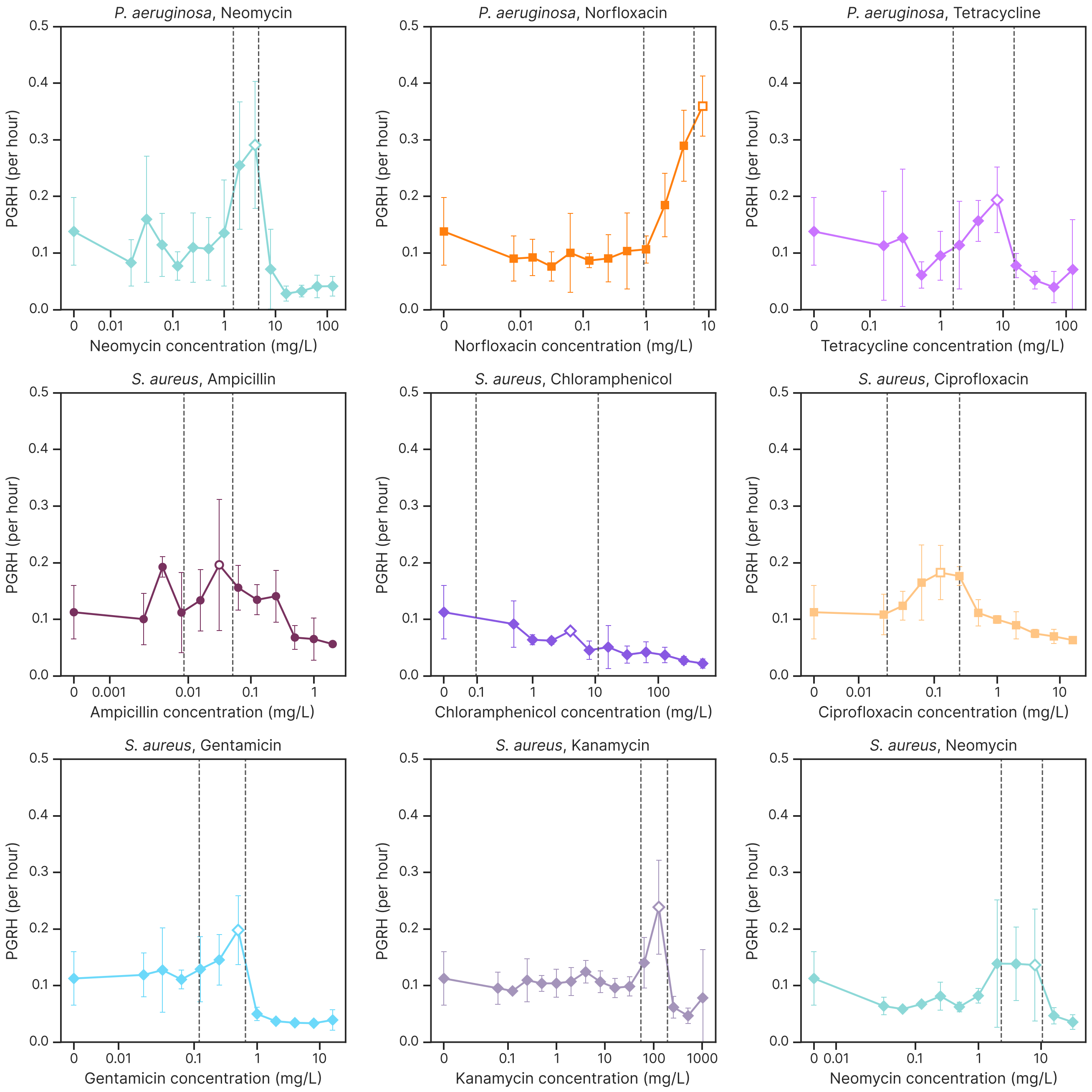

P. aeruginosa, Neomycin
P. aeruginosa, Norfloxacin
P. aeruginosa, Tetracycline
S. aureus, Ampicillin
S. aureus, Chloramphenicol
S. aureus, Ciprofloxacin
S. aureus, Gentamicin
S. aureus, Kanamycin
S. aureus, Neomycin
PGRH (per hour)
Neomycin concentration (mg/L)
Norfloxacin concentration (mg/L)
Tetracycline concentration (mg/L)
Ampicillin concentration (mg/L)
Chloramphenicol concentration (mg/L)
Ciprofloxacin concentration (mg/L)
Gentamicin concentration (mg/L)
Kanamycin concentration (mg/L)

S6C Fig

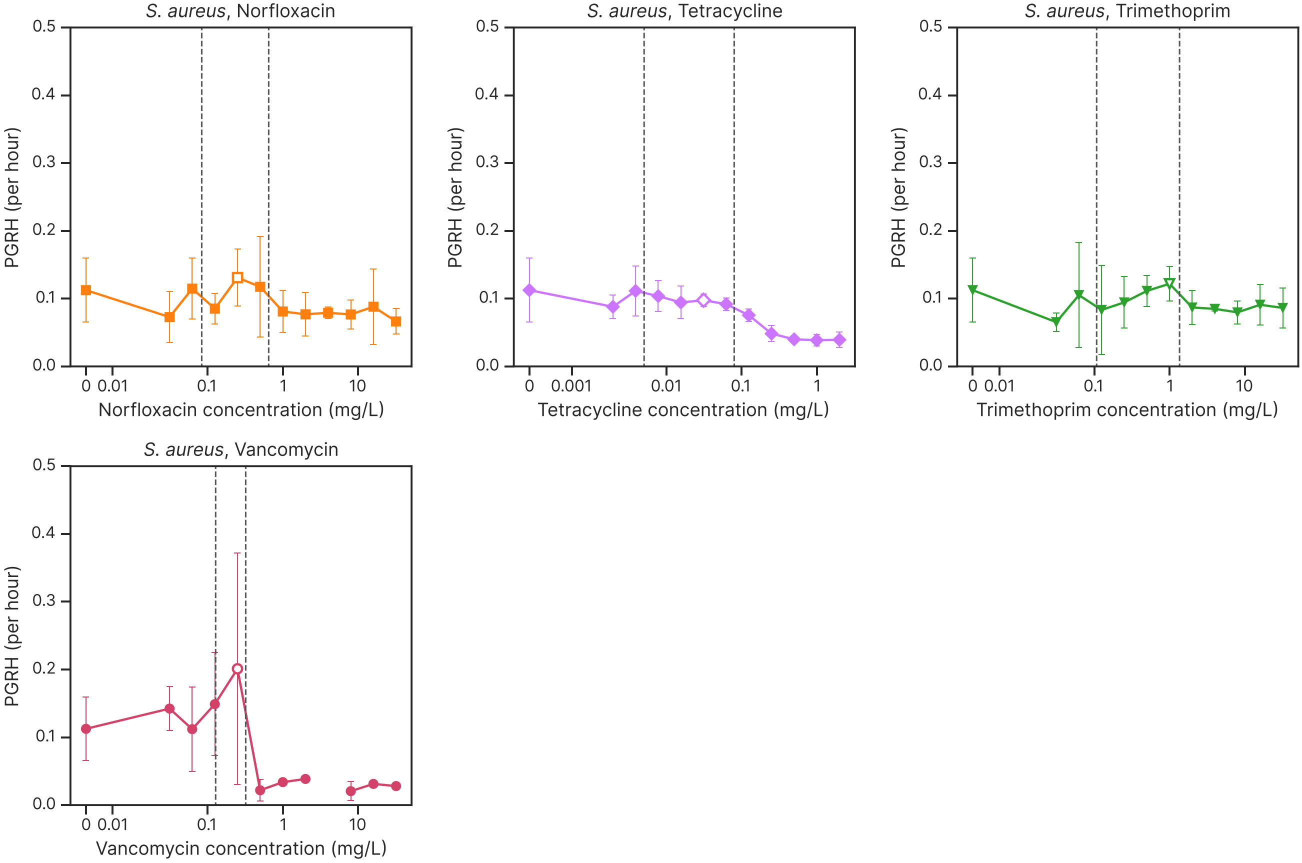
S. aureus, Norfloxacin
PGRH (per hour)
Norfloxacin concentration (mg/L)
S. aureus, Tetracycline
Tetracycline concentration (mg/L)
S. aureus, Trimethoprim
Trimethoprim concentration (mg/L)
S. aureus, Vancomycin
Vancomycin concentration (mg/L)

S6D Fig
