# Supplementary material for: Antibiotics change the population growth rate heterogeneity and morphology of bacteria
Source: PLoS Pathog. 2025 Feb 5;21(2):e1012924. doi: 10.1371/journal.ppat.1012924 (PMC11835381; doi:10.1371/journal.ppat.1012924)
Supplement: S9 Fig — Data is shown for PGRH at the peak in the vicinity of the MIC (left) as well as at the concentration closest to the MIC (right). These two correspond well generally, but notable exceptions exist, including for the DNA synthesis inhibitors where PGRH spikes at concentrations significantly higher than the MIC. Each marker shows the mean and standard deviation between repeats for a given antibiotic/species combination (three or more repeats per condition). Each antibiotic is allocated a separate hue. (PDF) [file ppat.1012924.s012.pdf]

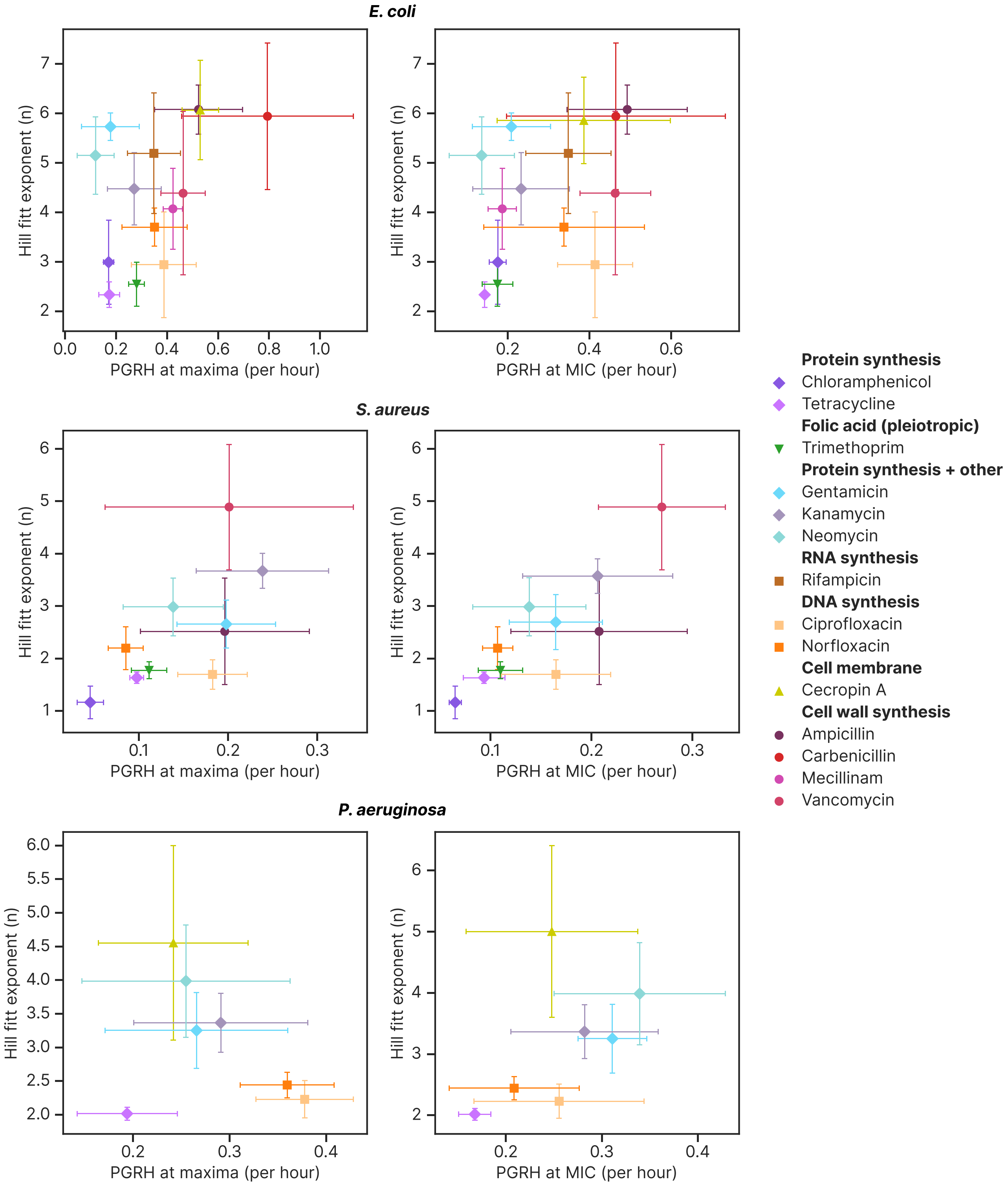
E. coli
S. aureus
P. aeruginosa
Hill fitt exponent (n)
PGRH at maxima (per hour)
PGRH at MIC (per hour)
Protein synthesis
Chloramphenicol
Tetracycline
Folic acid (pleiotropic)
Trimethoprim
Protein synthesis + other
Gentamicin
Kanamycin
Neomycin
RNA synthesis
Rifampicin
DNA synthesis
Ciprofloxacin
Norfloxacin
Cell membrane
Cecropin A
Cell wall synthesis
Ampicillin
Carbenicillin
Mecillinam
Vancomycin

S9 Fig
