# Supplementary material for: Antibiotics change the population growth rate heterogeneity and morphology of bacteria
Source: PLoS Pathog. 2025 Feb 5;21(2):e1012924. doi: 10.1371/journal.ppat.1012924 (PMC11835381; doi:10.1371/journal.ppat.1012924)
Supplement: S12 Fig — The boxplots show the median, quartiles, and whiskers show the 1.5 times the interquartile range. The plots for E.coli with ciprofloxacin, mecillinam and chloramphenicol are also presented in Fig 4 and are included here for completeness. (PDF) [file ppat.1012924.s015.pdf]

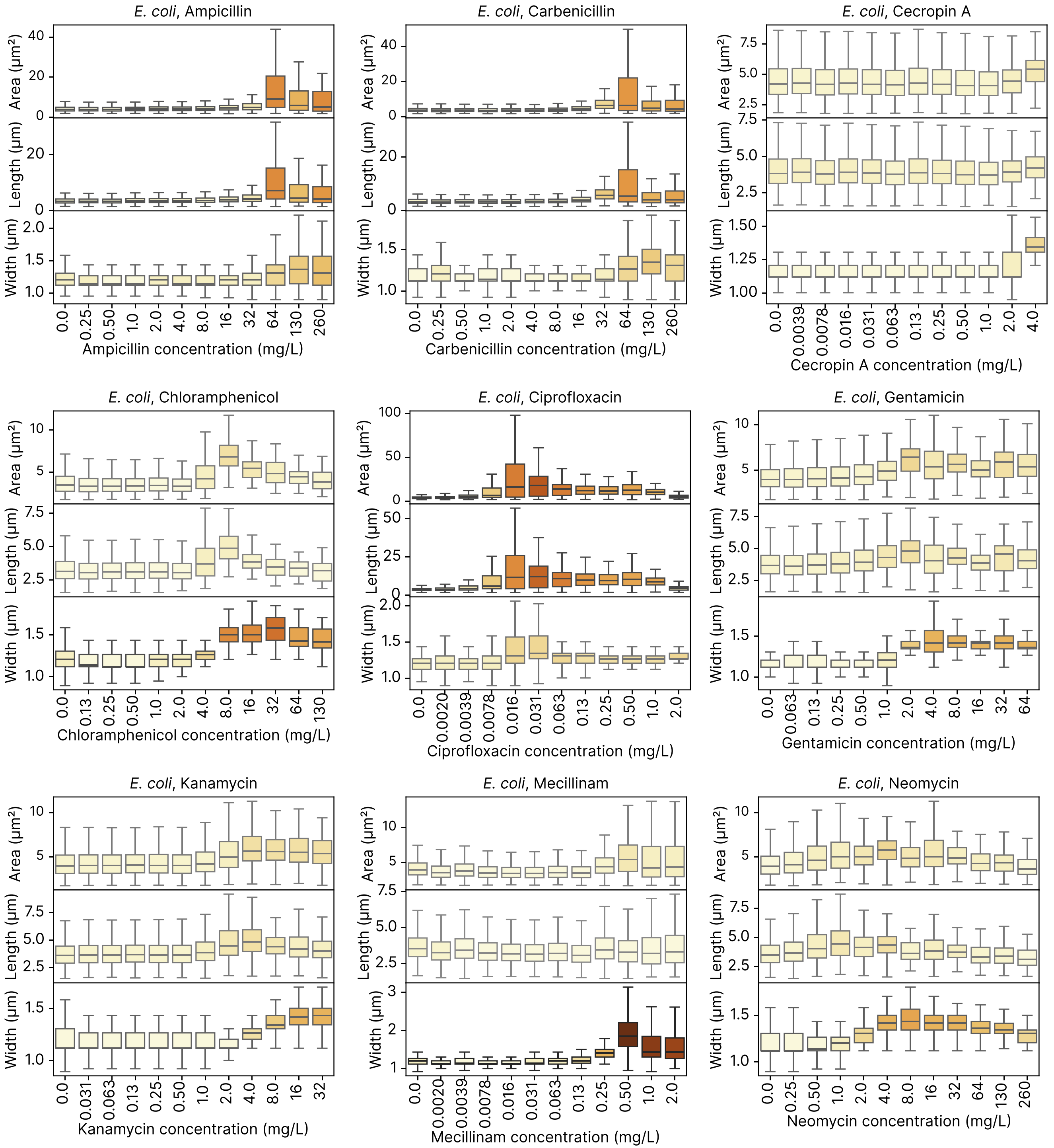
E. coli, Ampicillin
E. coli, Carbenicillin
E. coli, Cecropin A
E. coli, Chloramphenicol
E. coli, Ciprofloxacin
E. coli, Gentamicin
E. coli, Kanamycin
E. coli, Mecillinam
E. coli, Neomycin
Area (µm²)
Length (µm)
Width (µm)
Ampicillin concentration (mg/L)
Carbenicillin concentration (mg/L)
Cecropin A concentration (mg/L)
Chloramphenicol concentration (mg/L)
Ciprofloxacin concentration (mg/L)
Gentamicin concentration (mg/L)
Kanamycin concentration (mg/L)
Mecillinam concentration (mg/L)
Neomycin concentration (mg/L)

S12A Fig

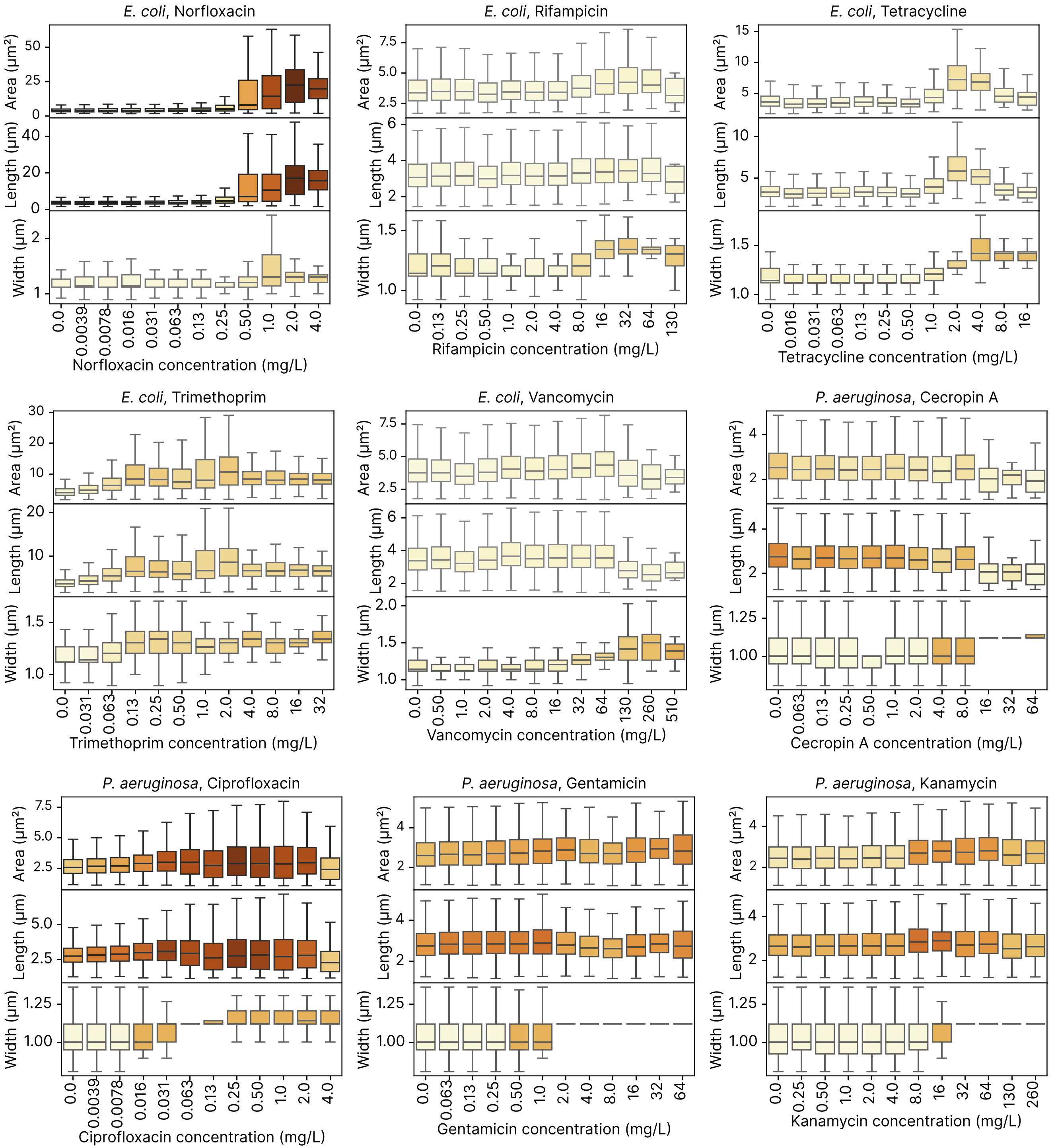
E. coli, Norfloxacin
Area (μm²)
Length (μm)
Width (μm)
0.0 0.0039 0.0078 0.016 0.031 0.063 0.13 0.25 0.50 1.0 2.0 4.0
Norfloxacin concentration (mg/L)
E. coli, Rifampicin
0.0 0.13 0.25 0.50 1.0 2.0 4.0 8.0 16 32 64 130
Rifampicin concentration (mg/L)
E. coli, Tetracycline
0.0 0.016 0.031 0.063 0.13 0.25 0.50 1.0 2.0 4.0 8.0 16
Tetracycline concentration (mg/L)
E. coli, Trimethoprim
0.0 0.031 0.063 0.13 0.25 0.50 1.0 2.0 4.0 8.0 16 32
Trimethoprim concentration (mg/L)
E. coli, Vancomycin
0.0 0.50 1.0 2.0 4.0 8.0 16 32 64 130 260 510
Vancomycin concentration (mg/L)
P. aeruginosa, Cecropin A
0.0 0.063 0.13 0.25 0.50 1.0 2.0 4.0 8.0 16 32 64
Cecropin A concentration (mg/L)
P. aeruginosa, Ciprofloxacin
0.0 0.0039 0.0078 0.016 0.031 0.063 0.13 0.25 0.50 1.0 2.0 4.0
Ciprofloxacin concentration (mg/L)
P. aeruginosa, Gentamicin
0.0 0.063 0.13 0.25 0.50 1.0 2.0 4.0 8.0 16 32 64
Gentamicin concentration (mg/L)
P. aeruginosa, Kanamycin
0.0 0.25 0.50 1.0 2.0 4.0 8.0 16 32 64 130 260
Kanamycin concentration (mg/L)

S12B Fig

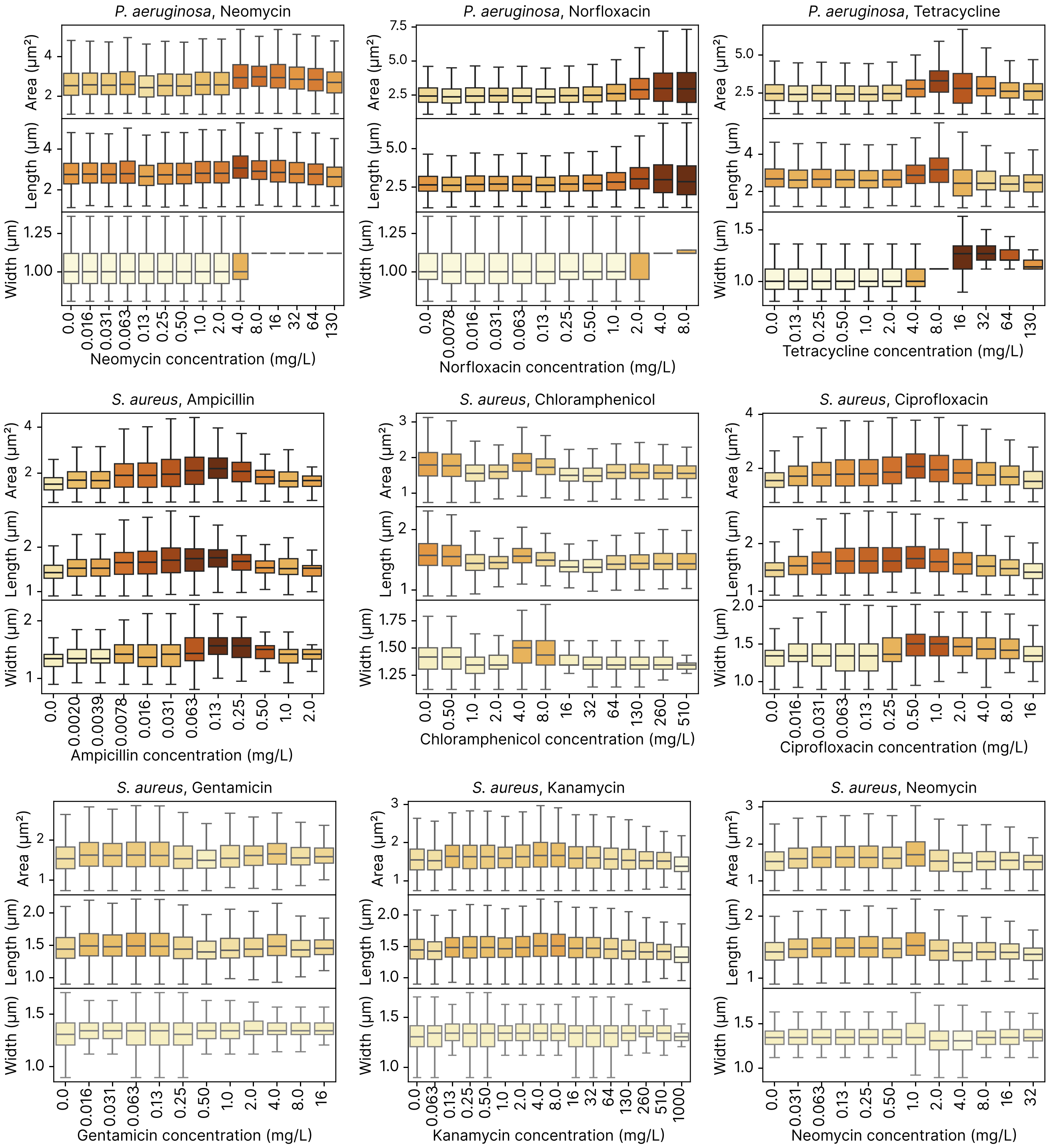
P. aeruginosa, Neomycin
P. aeruginosa, Norfloxacin
P. aeruginosa, Tetracycline
S. aureus, Ampicillin
S. aureus, Chloramphenicol
S. aureus, Ciprofloxacin
S. aureus, Gentamicin
S. aureus, Kanamycin
S. aureus, Neomycin
Area (μm²)
Length (μm)
Width (μm)
Neomycin concentration (mg/L)
Norfloxacin concentration (mg/L)
Tetracycline concentration (mg/L)
Ampicillin concentration (mg/L)
Chloramphenicol concentration (mg/L)
Ciprofloxacin concentration (mg/L)
Gentamicin concentration (mg/L)
Kanamycin concentration (mg/L)
Neomycin concentration (mg/L)

S12C Fig

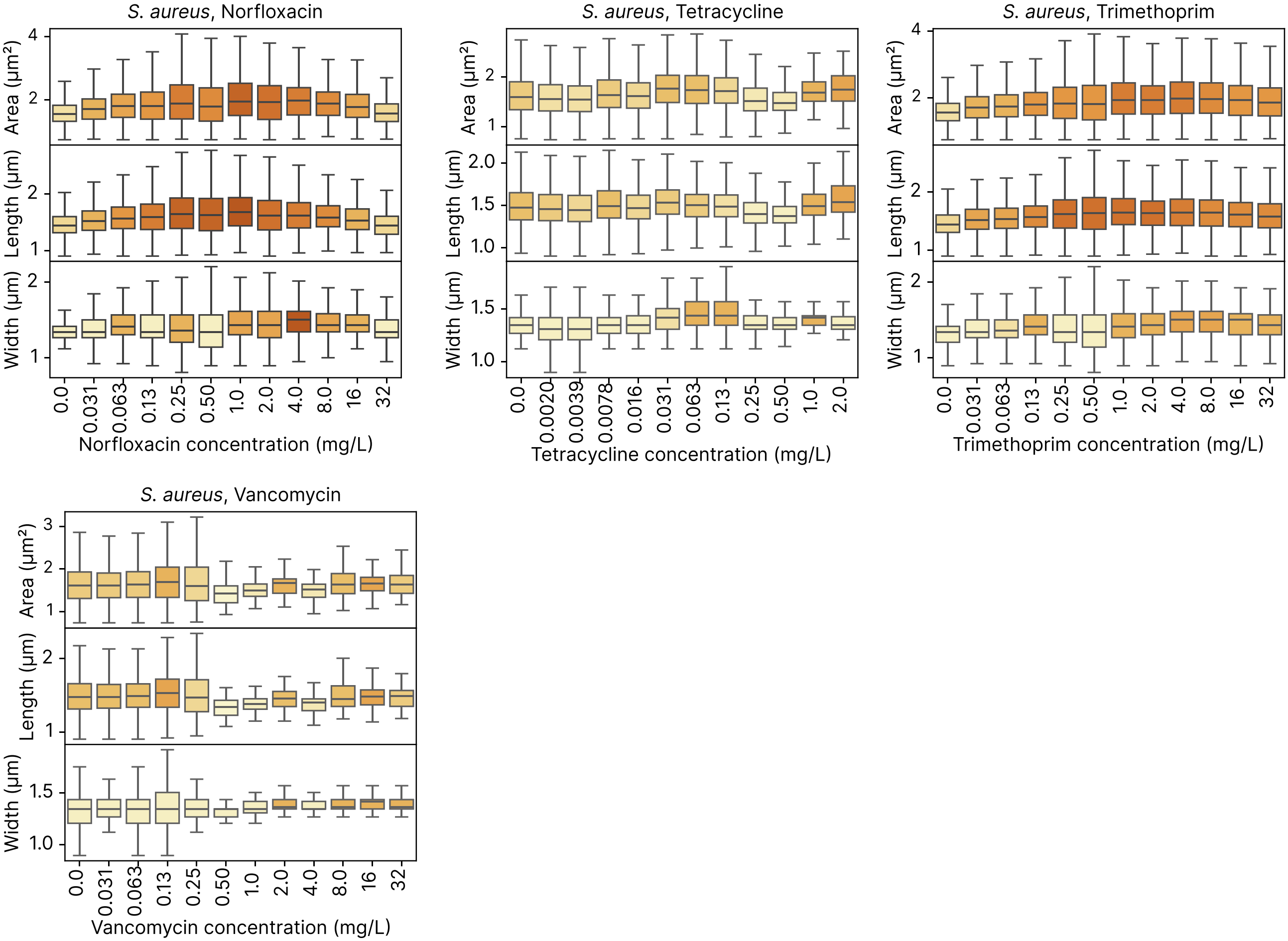
S. aureus, Norfloxacin
Area (μm²)
Length (μm)
Width (μm)
0.0
0.031
0.063
0.13
0.25
0.50
1.0
2.0
4.0
8.0
16
32
Norfloxacin concentration (mg/L)
S. aureus, Tetracycline
0.0
0.0020
0.0039
0.0078
0.016
0.031
0.063
0.13
0.25
0.50
1.0
2.0
Tetracycline concentration (mg/L)
S. aureus, Trimethoprim
Trimethoprim concentration (mg/L)
S. aureus, Vancomycin
Vancomycin concentration (mg/L)

S12D Fig
