# Supplementary material for: Antibiotics change the population growth rate heterogeneity and morphology of bacteria
Source: PLoS Pathog. 2025 Feb 5;21(2):e1012924. doi: 10.1371/journal.ppat.1012924 (PMC11835381; doi:10.1371/journal.ppat.1012924)
Supplement: S13 Fig — The points represent the mean and standard deviation of cell areas between repeats. The vertical lines represent IC10 and IC90, respectively. (PDF) [file ppat.1012924.s016.pdf]

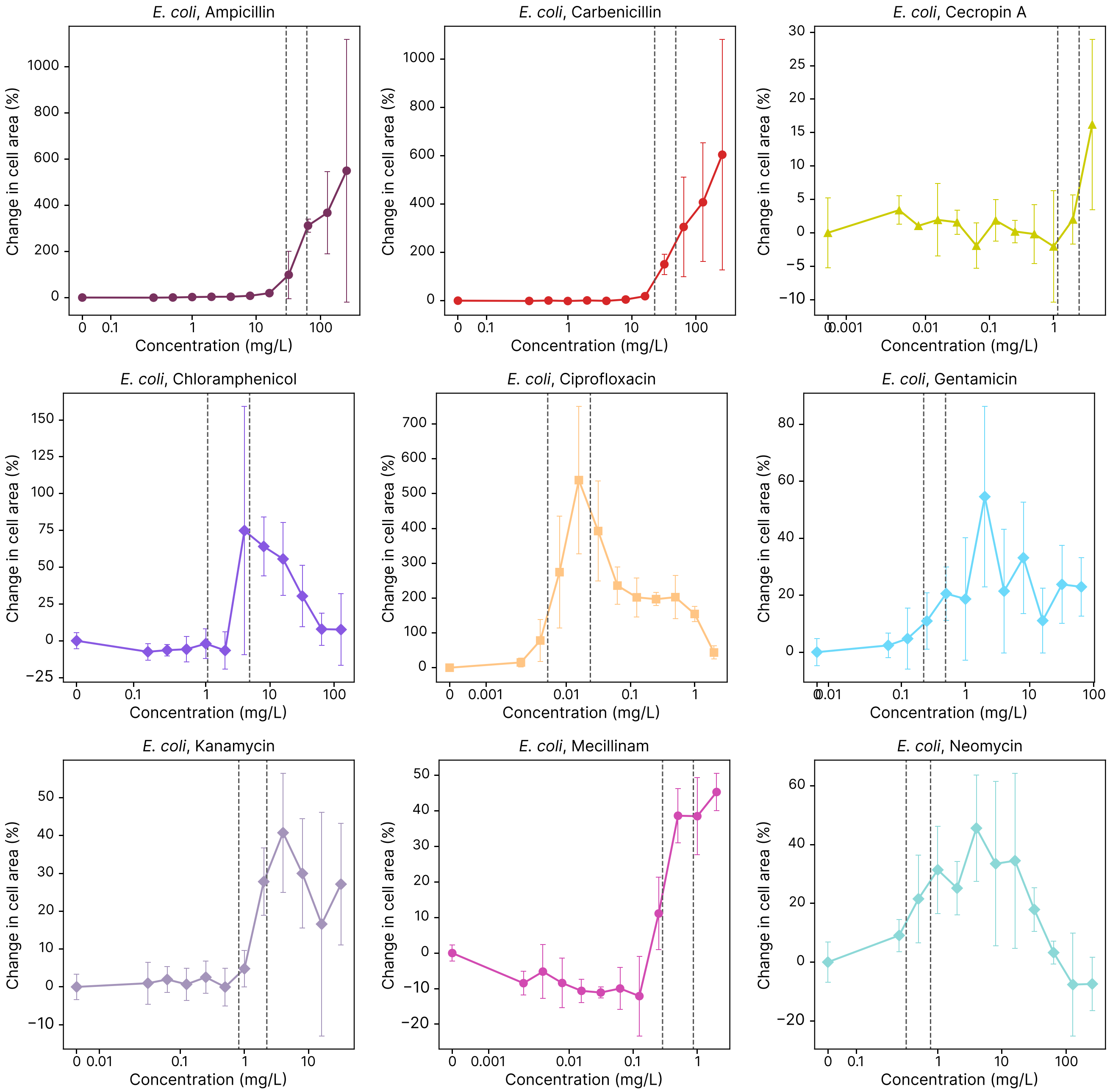
E. coli, Ampicillin
E. coli, Carbenicillin
E. coli, Cecropin A
E. coli, Chloramphenicol
E. coli, Ciprofloxacin
E. coli, Gentamicin
E. coli, Kanamycin
E. coli, Mecillinam
E. coli, Neomycin
Change in cell area (%)
Concentration (mg/L)

**S13A Fig**

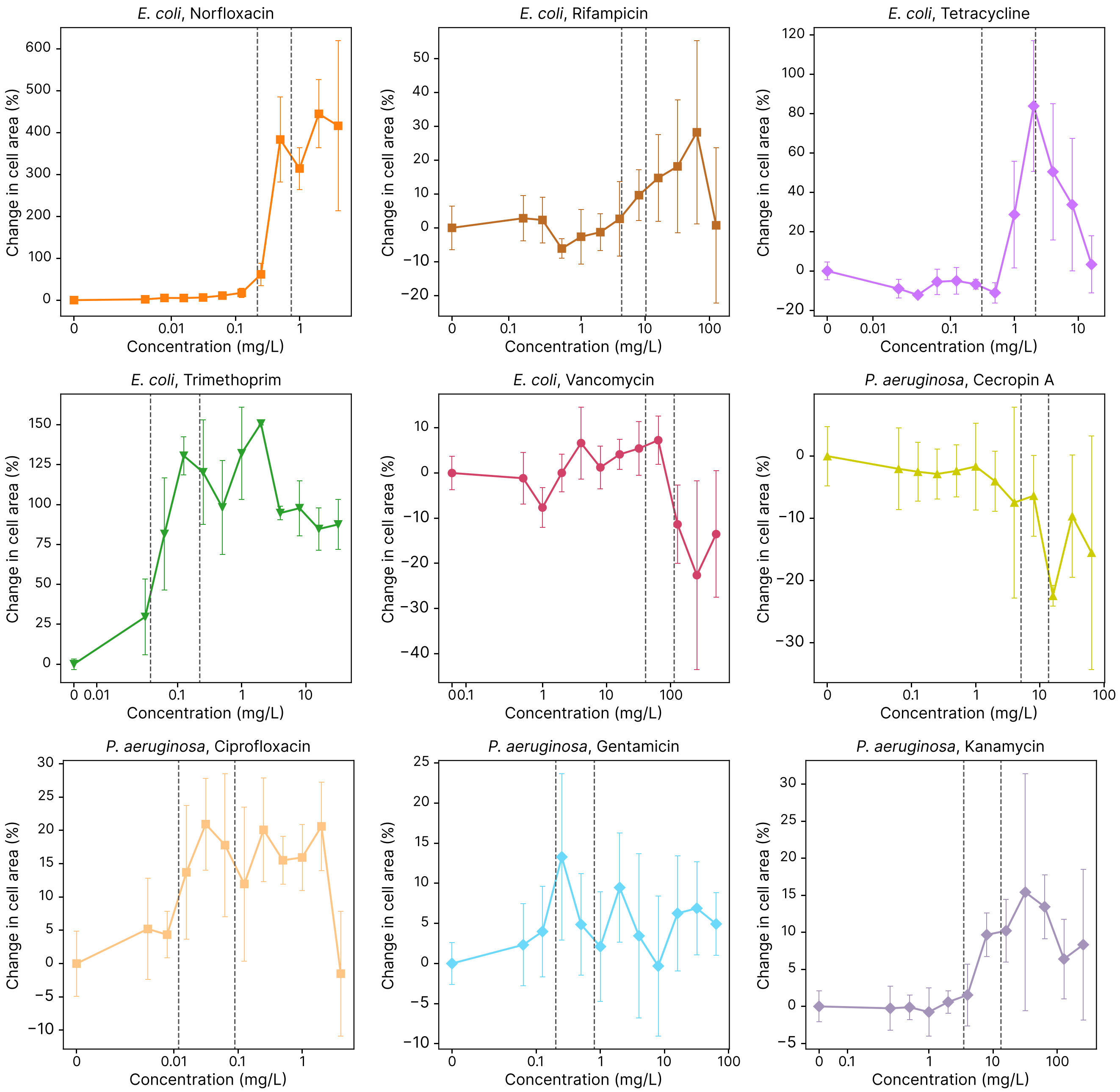

E. coli, Norfloxacin
E. coli, Rifampicin
E. coli, Tetracycline
E. coli, Trimethoprim
E. coli, Vancomycin
P. aeruginosa, Cecropin A
P. aeruginosa, Ciprofloxacin
P. aeruginosa, Gentamicin
P. aeruginosa, Kanamycin
Change in cell area (%)
Concentration (mg/L)


**S13B Fig**

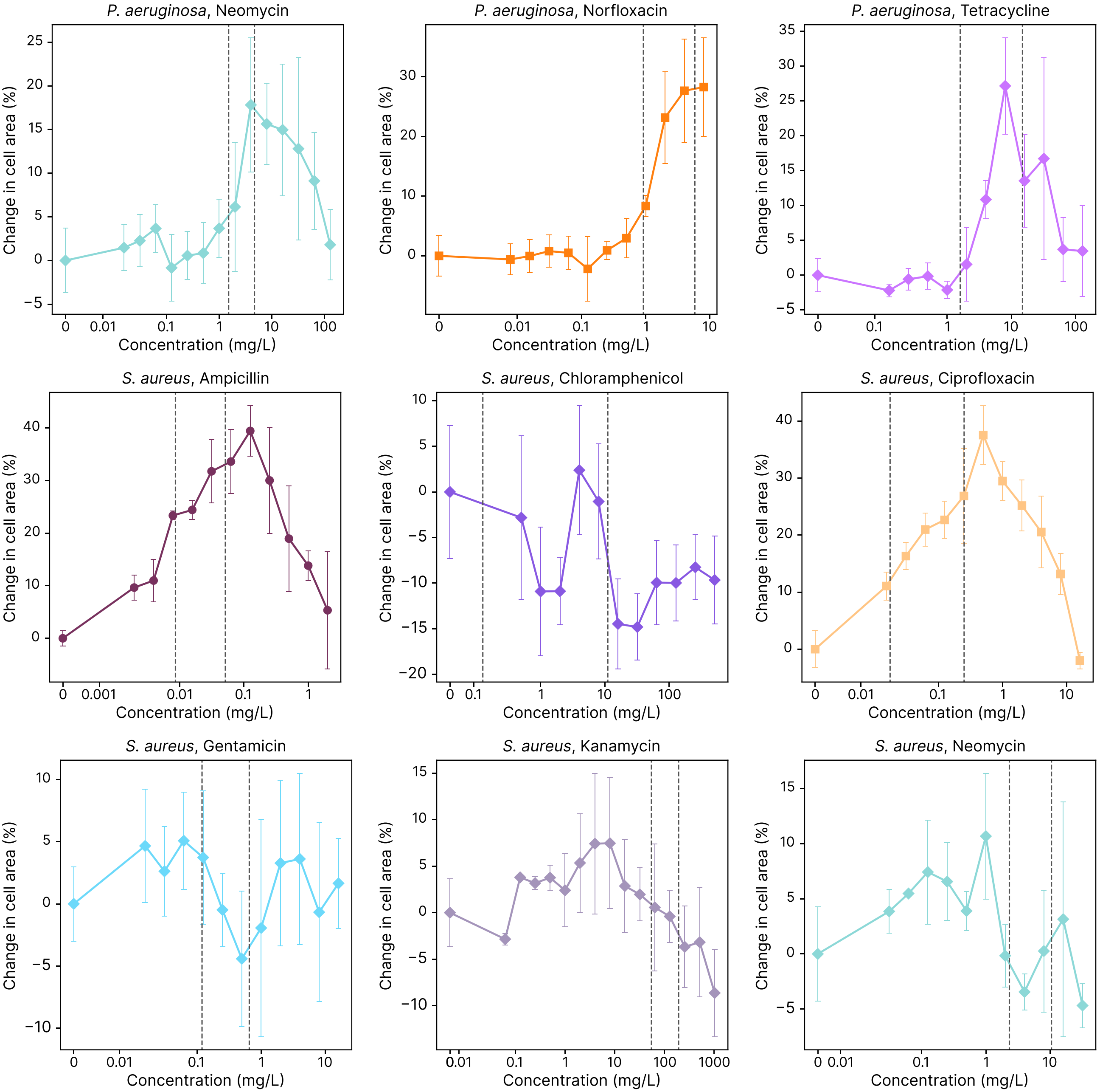

P. aeruginosa, Neomycin
P. aeruginosa, Norfloxacin
P. aeruginosa, Tetracycline
S. aureus, Ampicillin
S. aureus, Chloramphenicol
S. aureus, Ciprofloxacin
S. aureus, Gentamicin
S. aureus, Kanamycin
S. aureus, Neomycin
Change in cell area (%)
Concentration (mg/L)


**S13C Fig**

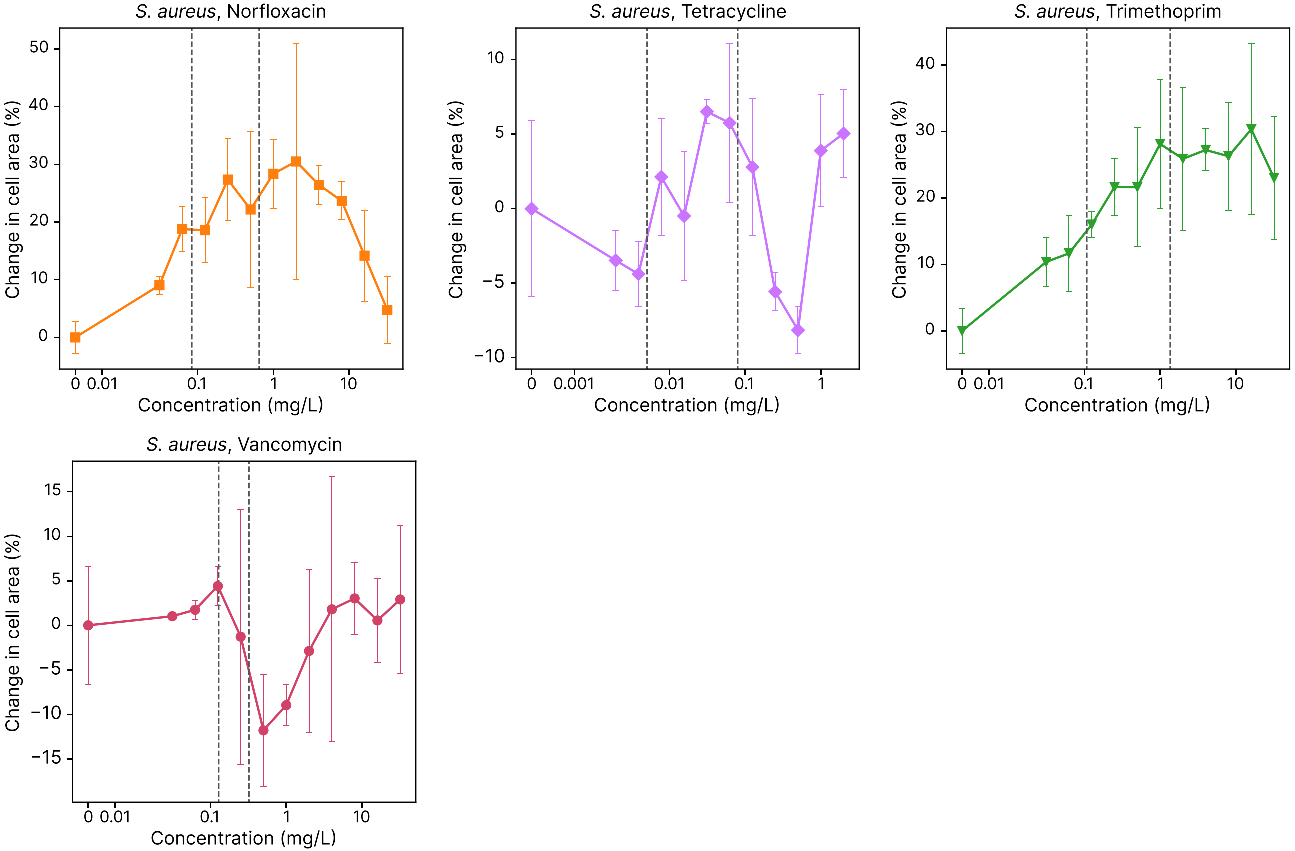
S. aureus, Norfloxacin
S. aureus, Tetracycline
S. aureus, Trimethoprim
S. aureus, Vancomycin
Change in cell area (%)
Concentration (mg/L)

**S13D Fig**
