# Supplementary material for: Antibiotics change the population growth rate heterogeneity and morphology of bacteria
Source: PLoS Pathog. 2025 Feb 5;21(2):e1012924. doi: 10.1371/journal.ppat.1012924 (PMC11835381; doi:10.1371/journal.ppat.1012924)
Supplement: S14 Fig — The points represent the mean and standard deviation in cell area across repeats. The size of the error bars is large, as they capture the variation in cell size caused by the cell cycle. The MOR50 threshold is halfway between the area with no antibiotic present and the maximum area change and is shown as a horizontal line. The cell MIC is where the area curve first crosses this threshold. This corresponds very closely with the IC50 point as determined by the growth rate, represented by the vertical grey line. The plots for E.coli with ciprofloxacin and vancomycin are also presented in Fig 7 and are included here for completeness. (PDF) [file ppat.1012924.s017.pdf]

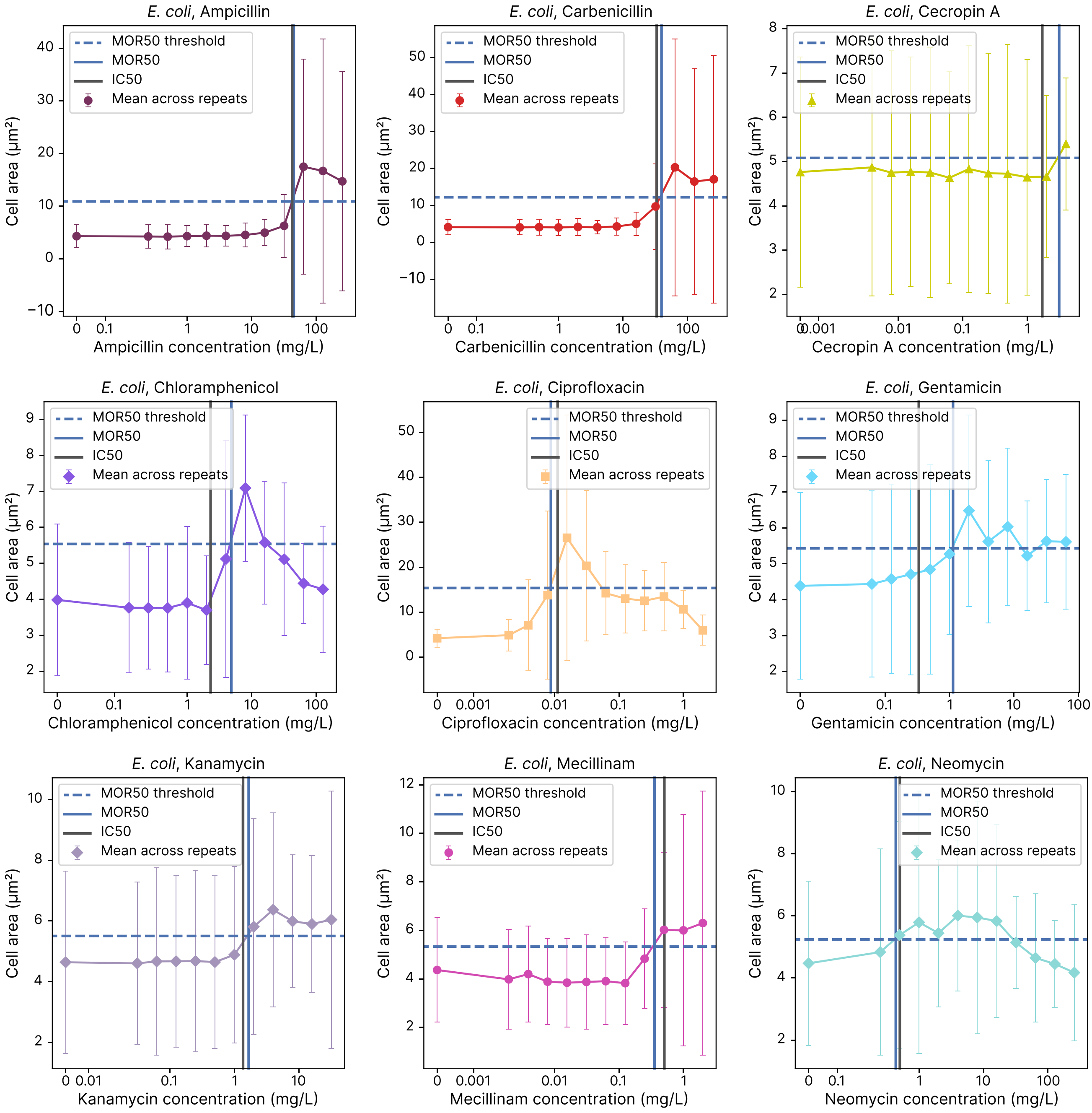
E. coli, Ampicillin
MOR50 threshold
MOR50
IC50
Mean across repeats
Cell area (μm²)
Ampicillin concentration (mg/L)
E. coli, Carbenicillin
Carbenicillin concentration (mg/L)
E. coli, Cecropin A
Cecropin A concentration (mg/L)
E. coli, Chloramphenicol
Chloramphenicol concentration (mg/L)
E. coli, Ciprofloxacin
Ciprofloxacin concentration (mg/L)
E. coli, Gentamicin
Gentamicin concentration (mg/L)
E. coli, Kanamycin
Kanamycin concentration (mg/L)
E. coli, Mecillinam
Mecillinam concentration (mg/L)
E. coli, Neomycin
Neomycin concentration (mg/L)

S14A Fig

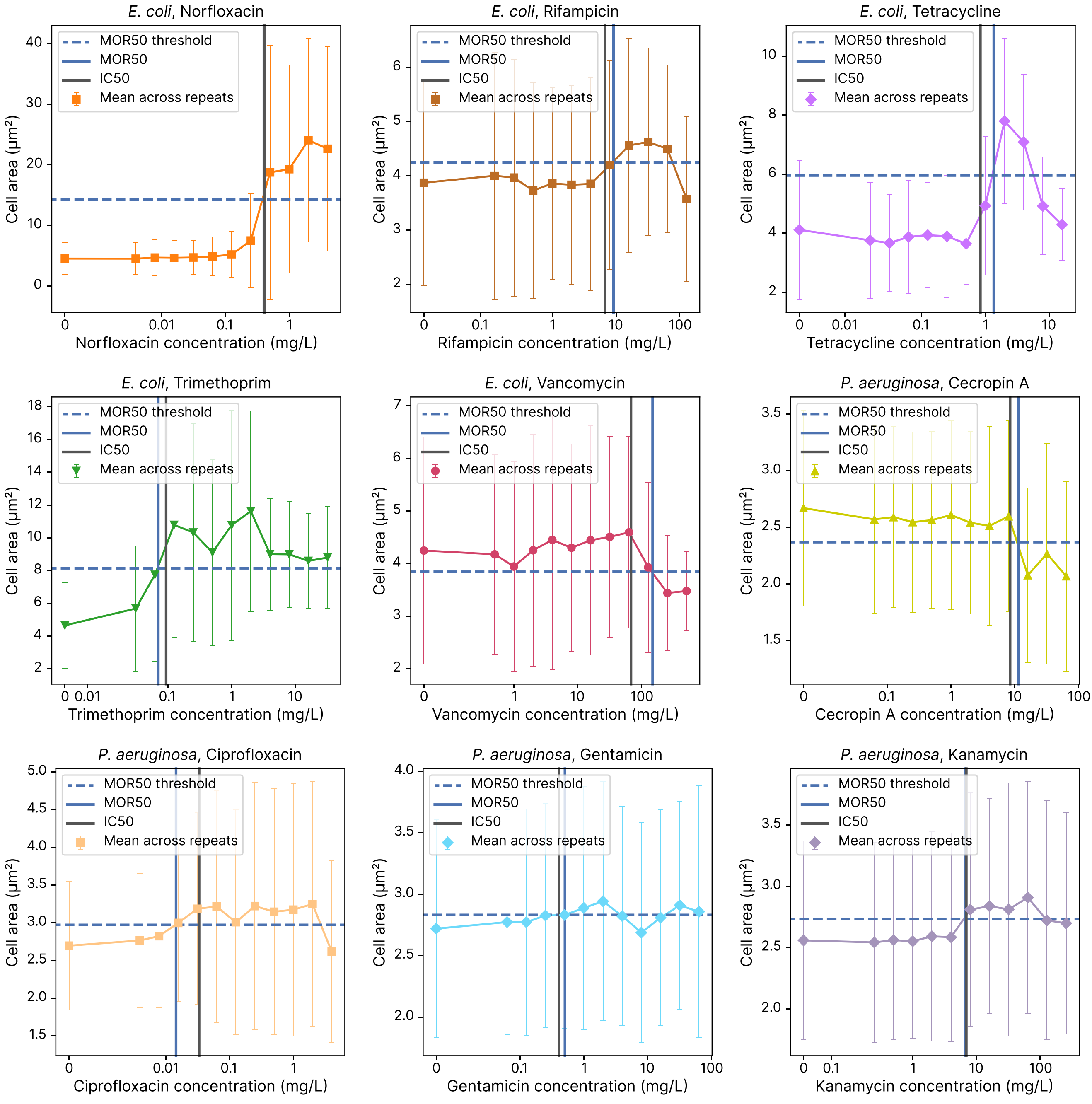
E. coli, Norfloxacin
E. coli, Rifampicin
E. coli, Tetracycline
E. coli, Trimethoprim
E. coli, Vancomycin
P. aeruginosa, Cecropin A
P. aeruginosa, Ciprofloxacin
P. aeruginosa, Gentamicin
P. aeruginosa, Kanamycin
MOR50 threshold
MOR50
IC50
Mean across repeats
Cell area (µm²)
Norfloxacin concentration (mg/L)
Rifampicin concentration (mg/L)
Tetracycline concentration (mg/L)
Trimethoprim concentration (mg/L)
Vancomycin concentration (mg/L)
Cecropin A concentration (mg/L)
Ciprofloxacin concentration (mg/L)
Gentamicin concentration (mg/L)
Kanamycin concentration (mg/L)

S14B Fig

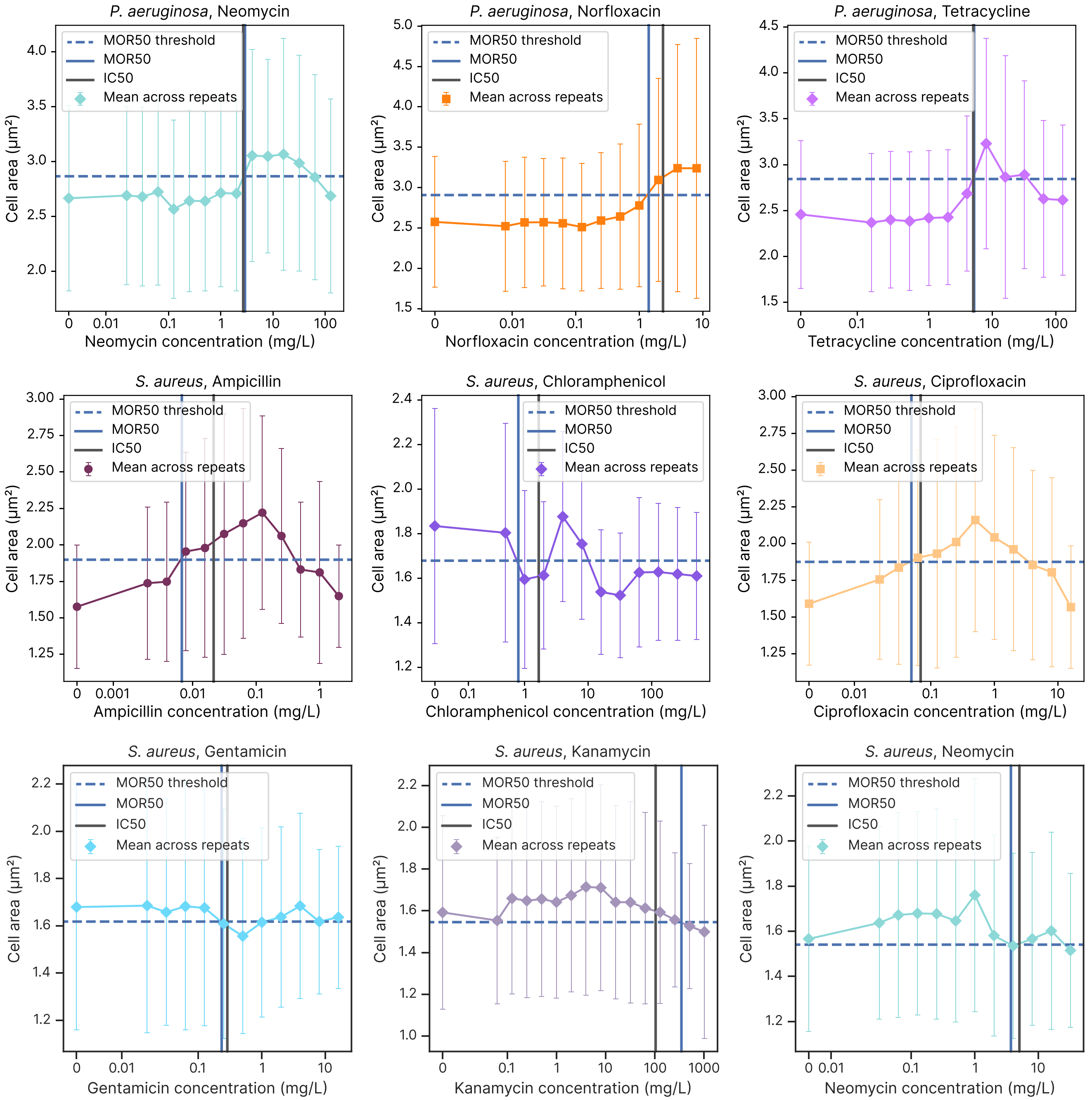

P. aeruginosa, Neomycin
P. aeruginosa, Norfloxacin
P. aeruginosa, Tetracycline
S. aureus, Ampicillin
S. aureus, Chloramphenicol
S. aureus, Ciprofloxacin
S. aureus, Gentamicin
S. aureus, Kanamycin
S. aureus, Neomycin
MOR50 threshold
MOR50
IC50
Mean across repeats
Cell area (μm²)
Neomycin concentration (mg/L)
Norfloxacin concentration (mg/L)
Tetracycline concentration (mg/L)
Ampicillin concentration (mg/L)
Chloramphenicol concentration (mg/L)
Ciprofloxacin concentration (mg/L)
Gentamicin concentration (mg/L)
Kanamycin concentration (mg/L)

S14C Fig

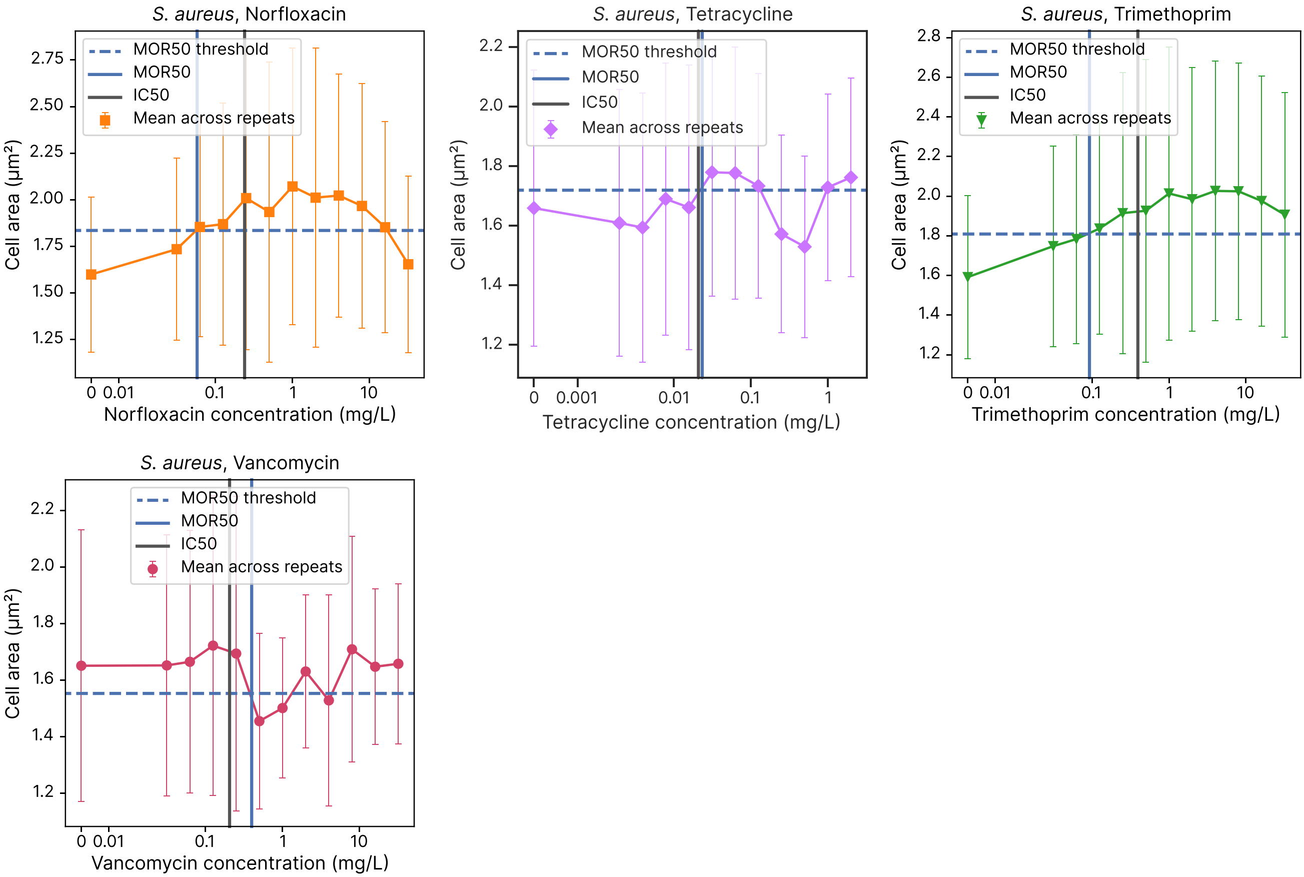
S. aureus, Norfloxacin
MOR50 threshold
MOR50
IC50
Mean across repeats
Cell area (µm²)
2.75
2.50
2.25
2.00
1.75
1.50
1.25
0
0.01
0.1
1
10
Norfloxacin concentration (mg/L)
S. aureus, Tetracycline
MOR50 threshold
MOR50
IC50
Mean across repeats
Cell area (µm²)
2.2
2.0
1.8
1.6
1.4
1.2
0
0.001
0.01
0.1
1
Tetracycline concentration (mg/L)
S. aureus, Trimethoprim
MOR50 threshold
MOR50
IC50
Mean across repeats
Cell area (µm²)
2.8
2.6
2.4
2.2
2.0
1.8
1.6
1.4
1.2
0
0.01
0.1
1
10
Trimethoprim concentration (mg/L)
S. aureus, Vancomycin
MOR50 threshold
MOR50
IC50
Mean across repeats
Cell area (µm²)
2.2
2.0
1.8
1.6
1.4
1.2
0
0.01
0.1
1
10
Vancomycin concentration (mg/L)

S14D Fig
