# Supplementary material for: Antibiotics change the population growth rate heterogeneity and morphology of bacteria
Source: PLoS Pathog. 2025 Feb 5;21(2):e1012924. doi: 10.1371/journal.ppat.1012924 (PMC11835381; doi:10.1371/journal.ppat.1012924)
Supplement: S21 Fig — A sample colony is used to illustrate each step, with a zoomed-in view for each step to highlight the cell details. The colony was captured after two hours of imaging. A First, the z-stack projection of the brightfield image is processed to generate a binary image containing the cell masks. B Then, the masks are converted to contours and manipulated to produce accurate segmentation for the bacteria. (PDF) [file ppat.1012924.s024.pdf]

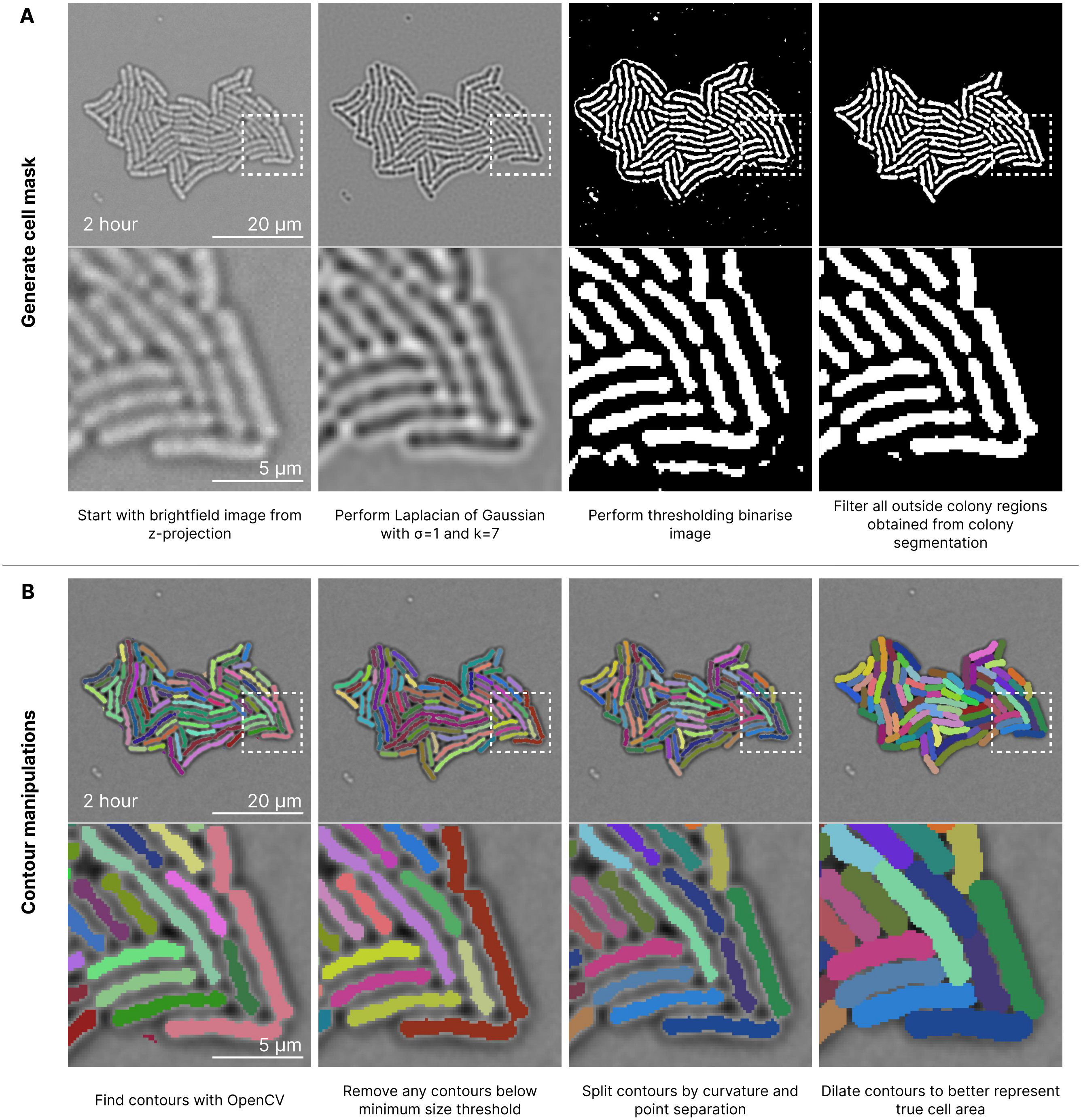
A
Generate cell mask
2 hour
20 µm
5 µm
Start with brightfield image from z-projection
Perform Laplacian of Gaussian with σ=1 and k=7
Perform thresholding binarise image
Filter all outside colony regions obtained from colony segmentation
B
Contour manipulations
2 hour
20 µm
5 µm
Find contours with OpenCV
Remove any contours below minimum size threshold
Split contours by curvature and point separation
Dilate contours to better represent true cell area

**S21 Fig**
