# Supplementary material for: Antibiotics change the population growth rate heterogeneity and morphology of bacteria
Source: PLoS Pathog. 2025 Feb 5;21(2):e1012924. doi: 10.1371/journal.ppat.1012924 (PMC11835381; doi:10.1371/journal.ppat.1012924)
Supplement: S22 Fig — Each curve shows the mean and standard deviation in OD600 over time for each antibiotic concentration over time. The broth microdilution MIC (MICBM) is selected as the lowest concentration of antibiotic that leads to a growth rate that does not exceed the red OD600 threshold before the black time threshold of 15 hours. For some antibiotic/species combinations, this never happens. For this antibiotic, we have marked the species as resistant. (PDF) [file ppat.1012924.s025.pdf]

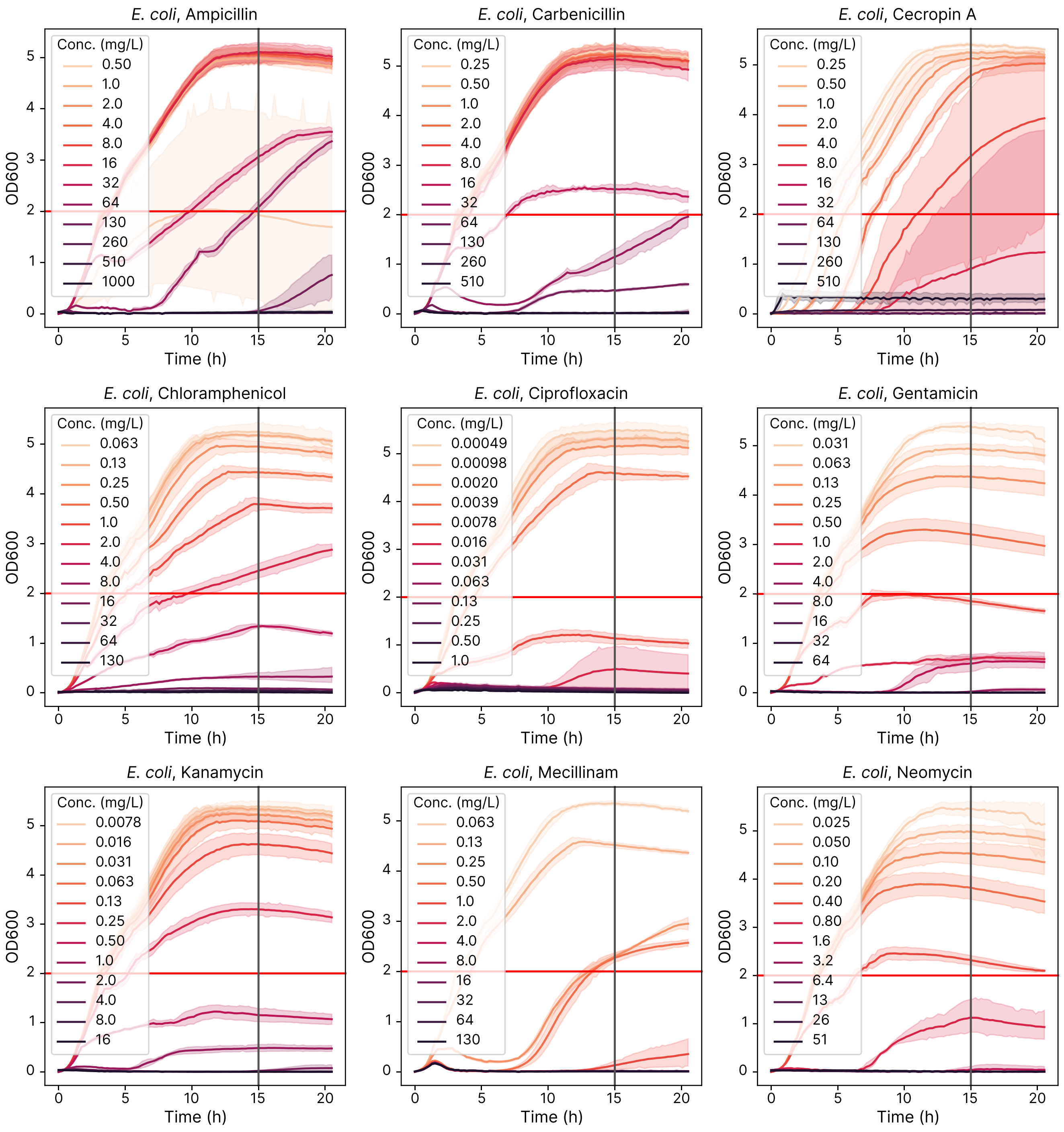
E. coli, Ampicillin
Conc. (mg/L)
0.50
1.0
2.0
4.0
8.0
16
32
64
130
260
510
1000
E. coli, Carbenicillin
Conc. (mg/L)
0.25
0.50
1.0
2.0
4.0
8.0
16
32
64
130
260
510
E. coli, Cecropin A
Conc. (mg/L)
0.25
0.50
1.0
2.0
4.0
8.0
16
32
64
130
260
510
E. coli, Chloramphenicol
Conc. (mg/L)
0.063
0.13
0.25
0.50
1.0
2.0
4.0
8.0
16
32
64
130
E. coli, Ciprofloxacin
Conc. (mg/L)
0.00049
0.00098
0.0020
0.0039
0.0078
0.016
0.031
0.063
0.13
0.25
0.50
1.0
E. coli, Gentamicin
Conc. (mg/L)
0.031
0.063
0.13
0.25
0.50
1.0
2.0
4.0
8.0
16
32
64
E. coli, Kanamycin
Conc. (mg/L)
0.0078
0.016
0.031
0.063
0.13
0.25
0.50
1.0
2.0
4.0
8.0
16
E. coli, Mecillinam
Conc. (mg/L)
0.063
0.13
0.25
0.50
1.0
2.0
4.0
8.0
16
32
64
130
E. coli, Neomycin
Conc. (mg/L)
0.025
0.050
0.10
0.20
0.40
0.80
1.6
3.2
6.4
13
26
51
OD600
Time (h)

S22A Fig

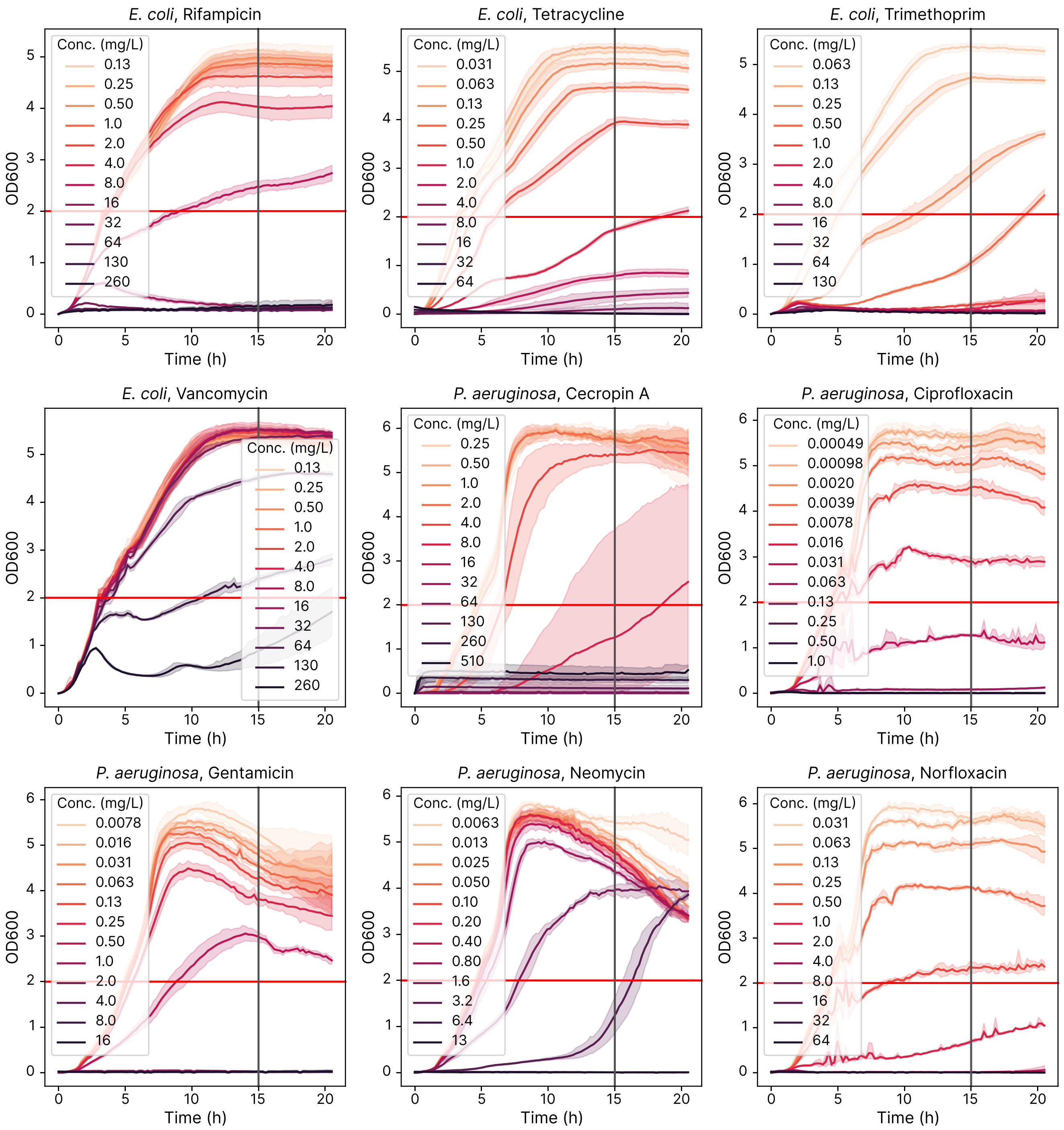
E. coli, Rifampicin
Conc. (mg/L)
0.13
0.25
0.50
1.0
2.0
4.0
8.0
16
32
64
130
260
E. coli, Tetracycline
Conc. (mg/L)
0.031
0.063
0.13
0.25
0.50
1.0
2.0
4.0
8.0
16
32
64
E. coli, Trimethoprim
Conc. (mg/L)
0.063
0.13
0.25
0.50
1.0
2.0
4.0
8.0
16
32
64
130
E. coli, Vancomycin
Conc. (mg/L)
0.13
0.25
0.50
1.0
2.0
4.0
8.0
16
32
64
130
260
P. aeruginosa, Cecropin A
Conc. (mg/L)
0.25
0.50
1.0
2.0
4.0
8.0
16
32
64
130
260
510
P. aeruginosa, Ciprofloxacin
Conc. (mg/L)
0.00049
0.00098
0.0020
0.0039
0.0078
0.016
0.031
0.063
0.13
0.25
0.50
1.0
P. aeruginosa, Gentamicin
Conc. (mg/L)
0.0078
0.016
0.031
0.063
0.13
0.25
0.50
1.0
2.0
4.0
8.0
16
P. aeruginosa, Neomycin
Conc. (mg/L)
0.0063
0.013
0.025
0.050
0.10
0.20
0.40
0.80
1.6
3.2
6.4
13
P. aeruginosa, Norfloxacin
Conc. (mg/L)
0.031
0.063
0.13
0.25
0.50
1.0
2.0
4.0
8.0
16
32
64
OD600
Time (h)

S22B Fig

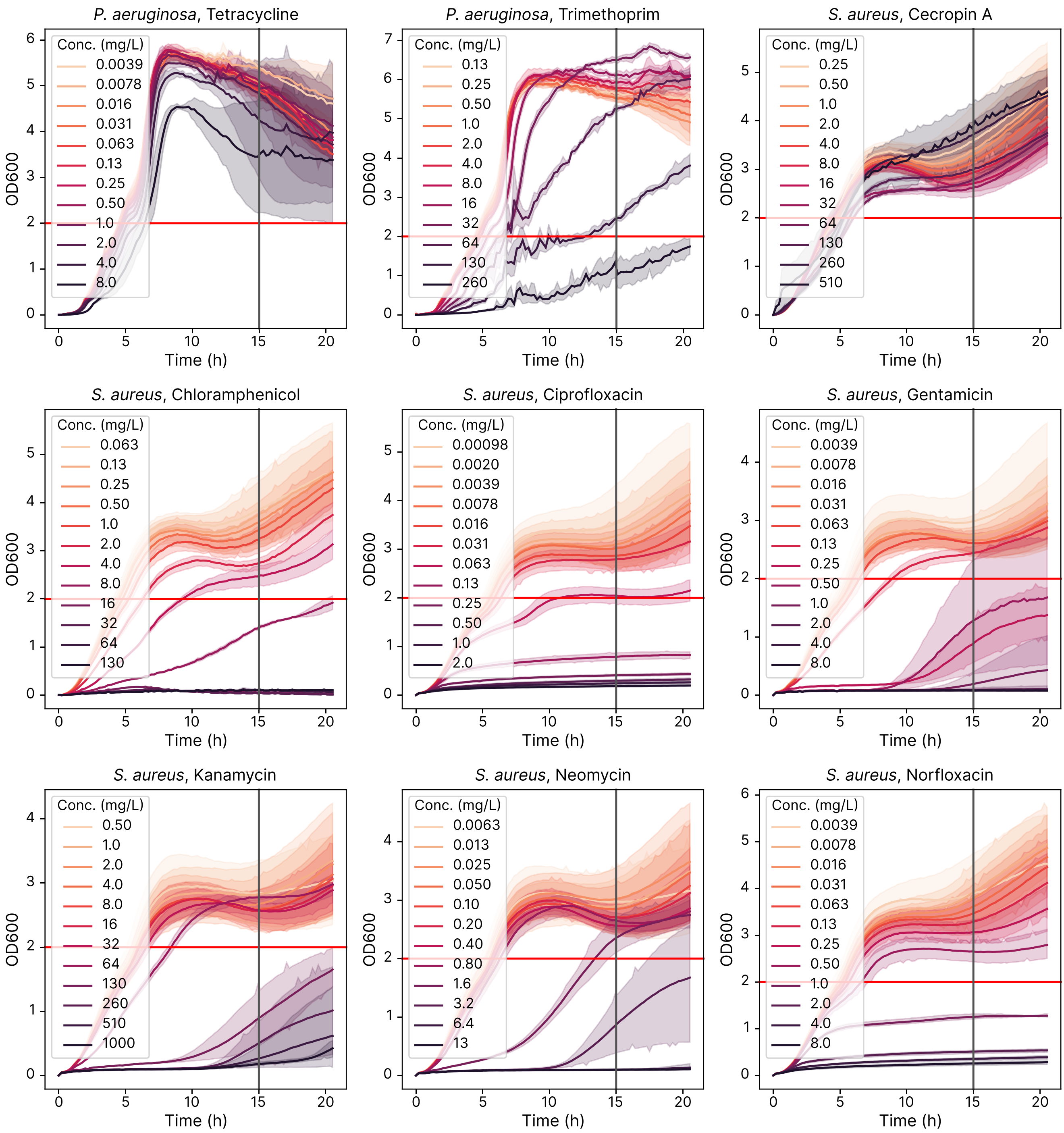
P. aeruginosa, Tetracycline
Conc. (mg/L)
0.0039
0.0078
0.016
0.031
0.063
0.13
0.25
0.50
1.0
2.0
4.0
8.0
P. aeruginosa, Trimethoprim
Conc. (mg/L)
0.13
0.25
0.50
1.0
2.0
4.0
8.0
16
32
64
130
260
S. aureus, Cecropin A
Conc. (mg/L)
0.25
0.50
1.0
2.0
4.0
8.0
16
32
64
130
260
510
S. aureus, Chloramphenicol
Conc. (mg/L)
0.063
0.13
0.25
0.50
1.0
2.0
4.0
8.0
16
32
64
130
S. aureus, Ciprofloxacin
Conc. (mg/L)
0.00098
0.0020
0.0039
0.0078
0.016
0.031
0.063
0.13
0.25
0.50
1.0
2.0
S. aureus, Gentamicin
Conc. (mg/L)
0.0039
0.0078
0.016
0.031
0.063
0.13
0.25
0.50
1.0
2.0
4.0
8.0
S. aureus, Kanamycin
Conc. (mg/L)
0.50
1.0
2.0
4.0
8.0
16
32
64
130
260
510
1000
S. aureus, Neomycin
Conc. (mg/L)
0.0063
0.013
0.025
0.050
0.10
0.20
0.40
0.80
1.6
3.2
6.4
13
S. aureus, Norfloxacin
Conc. (mg/L)
0.0039
0.0078
0.016
0.031
0.063
0.13
0.25
0.50
1.0
2.0
4.0
8.0
OD600
Time (h)

S22C Fig

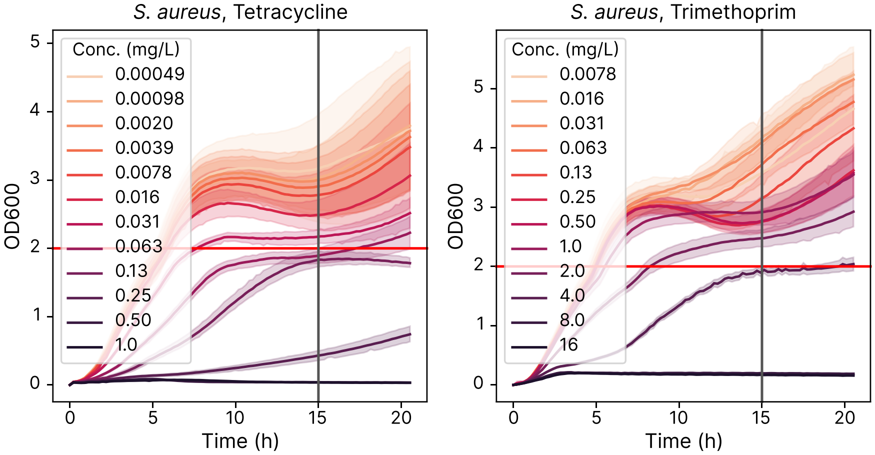
S. aureus, Tetracycline
Conc. (mg/L)
0.00049
0.00098
0.0020
0.0039
0.0078
0.016
0.031
0.063
0.13
0.25
0.50
1.0
OD600
Time (h)
0
5
10
15
20
1
2
3
4
S. aureus, Trimethoprim
Conc. (mg/L)
0.0078
0.016
0.031
0.063
0.13
0.25
0.50
1.0
2.0
4.0
8.0
16

S22D Fig
